# Delineating regions of interest for mass spectrometry imaging by multimodally corroborated spatial segmentation

**DOI:** 10.1093/gigascience/giad021

**Published:** 2023-04-11

**Authors:** Ang Guo, Zhiyu Chen, Fang Li, Qian Luo

**Affiliations:** Institute of Biomedicine and Biotechnology, Shenzhen Institute of Advanced Technology, Chinese Academy of Sciences, Shenzhen 518055, China; Institute of Biomedicine and Biotechnology, Shenzhen Institute of Advanced Technology, Chinese Academy of Sciences, Shenzhen 518055, China; University of Chinese Academy of Sciences, Beijing 100049, China; Institute of Biomedicine and Biotechnology, Shenzhen Institute of Advanced Technology, Chinese Academy of Sciences, Shenzhen 518055, China; Institute of Biomedicine and Biotechnology, Shenzhen Institute of Advanced Technology, Chinese Academy of Sciences, Shenzhen 518055, China; University of Chinese Academy of Sciences, Beijing 100049, China

**Keywords:** mass spectrometry imaging, spatial segmentation, multimodal data fusion

## Abstract

Mass spectrometry imaging (MSI), which localizes molecules in a tag-free, spatially resolved manner, is a powerful tool for the understanding of underlying biochemical mechanisms of biological phenomena. When analyzing MSI data, it is essential to delineate regions of interest (ROIs) that correspond to tissue areas of different anatomical or pathological labels. Spatial segmentation, obtained by clustering MSI pixels according to their mass spectral similarities, is a popular approach to automate ROI definition. However, how to select the number of clusters (#Clusters), which determines the granularity of segmentation, remains to be resolved, and an inappropriate #Clusters may lead to ROIs not biologically real. Here we report a multimodal fusion strategy to enable an objective and trustworthy selection of #Clusters by utilizing additional information from corresponding histology images. A deep learning–based algorithm is proposed to extract “histomorphological feature spectra” across an entire hematoxylin and eosin image. Clustering is then similarly performed to produce histology segmentation. Since ROIs originating from instrumental noise or artifacts would not be reproduced cross-modally, the consistency between histology and MSI segmentation becomes an effective measure of the biological validity of the results. So, #Clusters that maximize the consistency is deemed as most probable. We validated our strategy on mouse kidney and renal tumor specimens by producing multimodally corroborated ROIs that agreed excellently with ground truths. Downstream analysis based on the said ROIs revealed lipid molecules highly specific to tissue anatomy or pathology. Our work will greatly facilitate MSI-mediated spatial lipidomics, metabolomics, and proteomics research by providing intelligent software to automatically and reliably generate ROIs.

## Introduction

The alterations and interactions of biochemical pathways are often spatially heterogeneous in a complex biological system, and thus localizing molecules in a spatially resolved manner is crucial for deciphering underlying biochemical mechanisms of biological phenomena. Mass spectrometry imaging (MSI) is a tag-free, high-throughput molecular mapping technique, which simultaneously acquires the spatial distributions of tens to hundreds of molecules by collecting a full mass spectrum in each pixel of a virtual grid [1, 2]. MSI can cover a wide variety of biomolecular species (including proteins, peptides, lipids, and metabolites) over biological samples with great sensitivity and chemical specificity [1, 2]. Since its emergence in the early 2000s [3, 4], MSI has enabled new biochemical discoveries in a wide range of life sciences, including oncology [5], neurology [6], microbiology [7], and drug development [8]. During the postacquisition analysis of MSI data, an intact tissue section is often virtually segmented into a number of regions of interest (ROIs) that correspond to different anatomical or pathological labels [1, 9]. An accurate definition of ROIs allows the extraction of tissue-type specific molecular abundances, which are essential for statistically discovering molecular alterations between different ROIs of the same specimen (for example, tumor versus normal regions of a tissue section) or between different specimens at the same ROI (for example, differentially expressed molecules at a specific anatomical structure between diseased versus healthy samples).

**Figure 1:**
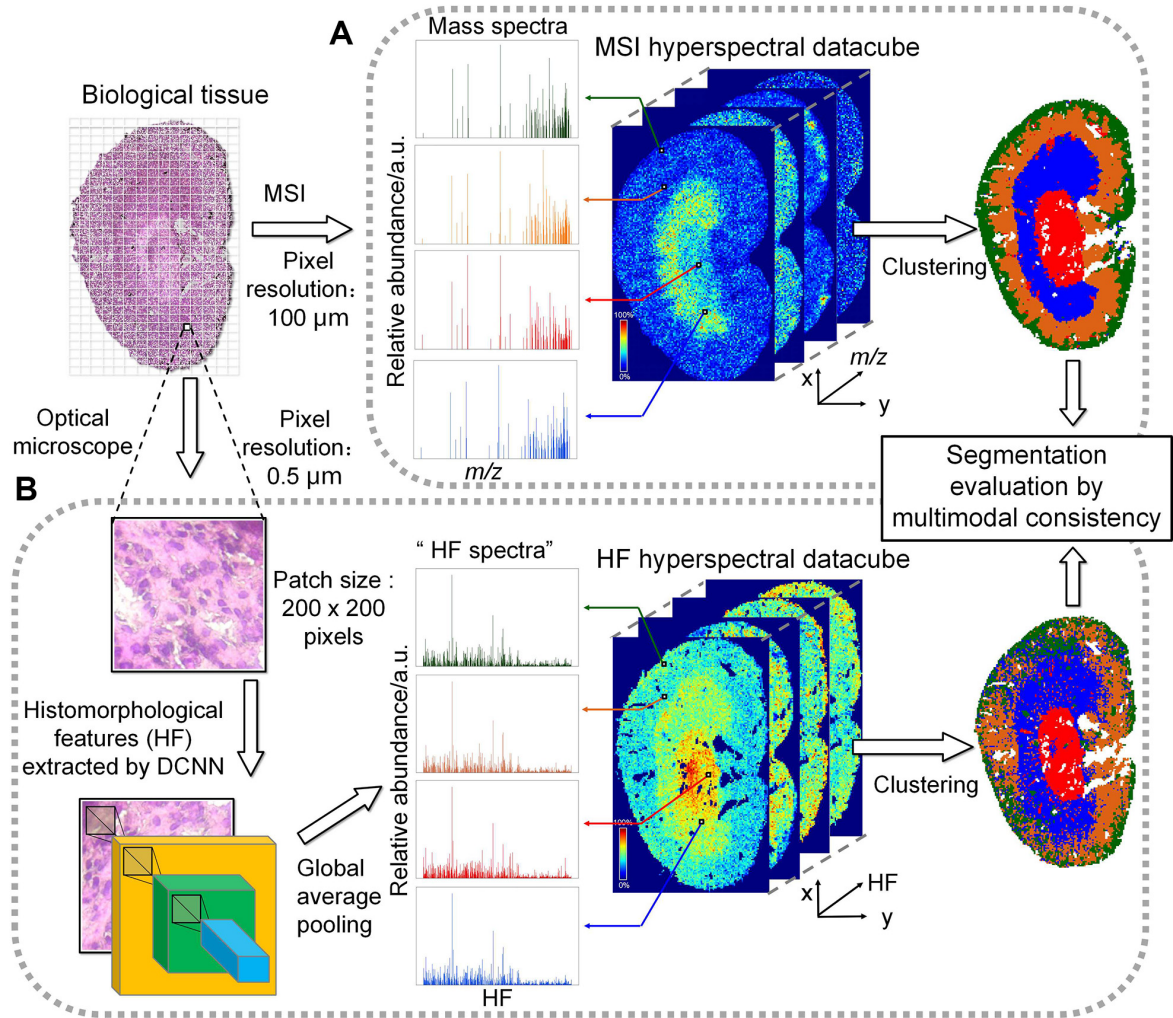
The overview of tissue segmentation based on (A) MSI data and (B) H&E-stained histology image. (A) Mass spectra acquired by MSI can be formatted as a hyperspectral data cube. (B) A high-resolution histology image was divided into an array of small tiles of size 200 × 200 pixels. A set of quantitative histomorphological features (HFs) was then computed from each tile by a DCNN-based feature extractor. So another hyperspectral data cube similar to the MSI data was generated, with the only difference that the depth corresponded to HF rather than *m*/*z*. Clustering analysis in the spectral domain resulted in segmentation in the spatial domain. Segmentation/ROI validation was achieved by comparing the MSI- and histology-based results.

There are 2 commonly used methods for defining ROIs. The first is manual annotation guided by the staining microscopy of a tissue specimen section (or its consecutive slice) [9]. Manual annotation requires solid expert knowledge of histology and is prone to human bias. It can also be rather time-consuming when the sample size and/or the quantity of ROIs are large. Spatial segmentation [10] is the alternative approach for ROI definition, which is data driven in nature and substantially automated. For a set of mass spectral profiles collected at different positions (i.e., pixels), a clustering algorithm separates them into several groups (i.e., clusters) in such a way that spectra in the same group are more “similar” to each other than to those in other groups. Pixels of identical tissue types are expected to be similar in their chemical content and thus mass spectral profiles. So, by labeling each pixel with the color assigned to its cluster, we can obtain spatial segmentation along the image domain, as shown in Fig. 1A. Following the pioneering work of Mccombie et al. [11], the use of clustering analysis to discover geographically separated and molecularly distinct tissue regions from MSI data has been increasingly adopted by the MSI community [12–18]. However, it remains a challenge to properly configure the parameters of clustering algorithms, which have a huge impact on final segmentation results. For instance, the number of clusters (#Clusters) determines the number of regions (i.e., the granularity of spatial segmentation) for algorithms such as k-means and spectral clustering, but it is arbitrarily predefined by users. As #Clusters gets larger, the clustering algorithm would start to segment the specimen on the basis of instrumental noise or acquisition artifacts, producing ROIs that are not biologically real. Such difficulty in setting parameters is largely owing to the lack of a method for rigorously evaluating segmentation results. So far in the MSI community, segmentation has been evaluated either by subjective judgments [12, 13, 16, 17, 19] or by certain internal clustering validation criteria [15, 20, 21]. The internal clustering validation [22] evaluates the “goodness” of clustering based only on the intrinsic structure of the MSI data, making it still susceptible to the influence of noise or artifacts. Also, different internal validation criteria can be built based on completely different rationales and may give rise to inconsistent outcomes [23], which further reduces their reliability. Consequently, there is an urgent need for an objective and biologically reliable approach to evaluating MSI segmentation results and determining the key parameters of clustering algorithms.

Integrating the data acquired by 2 or more biomedical imaging modalities is termed *multimodal fusion*, which holds the promise of enhancing the discrimination between modality-specific instrumental noise or artifacts and biologically relevant chemical signals [24, 25]. Specifically, segmentation based on a modality orthogonal to MSI can provide an external reference to evaluate the results generated by MSI. Of all the different imaging modalities, light microscopy is one of the most ideal candidates because of its good accessibility to the MSI community. For example, by integrating matrix-assisted laser desorption ionization (MALDI)–MSI and microscopy, Rappez et al. [26] have developed a SpaceM method to characterize in situ single-cell metabolomics. In addition, the past decade has seen the phenomenal success of deep learning [27] in the analysis of histology microscopic images [28]. Based on a well-established idea called “transfer learning” [29], deep convolutional neural networks (DCNNs), which pretrained using source datasets ImageNet [30], appear to be excellent off-the-shelf feature extractors to represent histology images with histomorphologically informative features, which have been used to achieve accurate classification of cell or tissue types [31]. Therefore, it becomes technically feasible to calculate histology-based segmentation according to histomorphological features (HFs) extracted from the histology image.

In this article, we report a multimodal fusion strategy between histology microscopy and MSI to enable an objective and trustworthy selection of #Clusters. First, an in-house algorithm was proposed to generate histology segmentation from a hematoxylin and eosin (H&E) histology image. As shown in Fig. 1, due to different lateral resolutions, each grid-scan pixel of MSI corresponds to a small tile of the whole-slide H&E image of the same specimen. By propagating a tile through a DCNN-based feature extractor, we constructed an “HF spectrum” to represent the histomorphology of cellular neighborhoods located within an MSI pixel. Analogous to the MSI segmentation, histology segmentation was obtained by clustering the pixels according to their HF spectral similarities. Second, built upon an assumption that more reliable segmentation would be better replicated by orthogonal imaging modalities, we used the multimodal consistency between histology and MSI segmentation as a quantitative measure to evaluate validity. So, different #Clusters were compared accordingly, and the one that produced maximum consistency was deemed most probable. Last, using the optimal #Clusters, pixels concordantly labeled by the 2 modalities were returned as ROIs of biological relevance supported by both molecular and histomorphological profiling. In the following sections, we used whole mouse kidney and tumor specimens as proof-of-concept examples to validate our strategy: ROIs produced by our strategy were in excellent agreement with ground truths. Based on the said ROIs, downstream data analysis revealed lipids that were highly specific to tissue anatomy or pathology: for the kidney specimen, PC(28:0), SM(d34:1), PC(34:3), and PC(38:5) appeared to be colocalized with the pelvis, inner cortex, outer cortex, and medulla, respectively; for the tumor specimen, cholesterol, PC(34:1), and PC(38:5) were colocalized with the necrosis, viable tumor, and normal tissues, respectively. We expect to facilitate MSI data analysis by offering an intelligent tool for ROI delineation that is biologically reliable and intrinsically immune to subjective bias.

## Materials and Methods

### Sample preparation and data acquisition

The animal experiments were approved by the Animal Care and Use Committee of Shenzhen Institute of Advanced Technology, Chinese Academy of Sciences. A mouse kidney (7-month-old female C57) was frozen-preserved at −80°C before being cut into 2 consecutive slices by cryostat sectioning with a thickness of 20 μm and 10 μm, respectively. MSI was done on the thicker slice with a 100 × 100-μm step size (i.e., pixel resolution) using a desorption spray ionization (DESI) ion source (Prosolia, Indianapolis, IN, USA) coupled to a Synapt G2-Si mass spectrometer (Waters, Manchester, UK). High-performance liquid chromatography–grade methanol (Merck, Darmstadt, Germany) was used in DESI experiments at a flow rate of 1.5 μL/min. In the positive ion mode, the capillary voltage of DESI was 4.2 kV with a sampling cone of 80 V. The nebulizing nitrogen gas pressure of DESI was 0.5 MPa and the ion transfer capillary was at a temperature of 150°C. A mass range of 200 to 1,000 *m*/*z* was covered with a mass resolving power of 10,000. The thinner slice went though a standard H&E staining protocol, followed by digital pathology scanning with 20× magnification (i.e., 0.5 × 0.5-μm pixel resolution). A murine model of human renal adenocarcinoma was established by orthotopically transplanting the ACHN cell line (Zhuhai BesTest Bio-Tech, Zhuhai, China) into NOD/SCID male mice at 4 weeks of age and growing for 5 more weeks before sacrifice. Being capable of mimicking the organ-specific tumor environment in humans, orthotopic models have been routinely used in the MSI community for understanding the biomolecular mechanisms underlying cancers and for investigating therapeutic agents [32]. The same experimental procedures were followed for the tumor specimen, except that the pixel resolution of MSI was set to 50 × 50 μm^2^ in adaptation to a small tumor and surrounding tissue area.

### Data processing and analysis

#### MSI and H&E data preprocessing

Raw MSI data were preprocessed with the R Cardinal 2 package [33] following standard data preprocessing protocols, including total ion count normalization, spectral smoothing, baseline reduction, peak picking, peak alignment, peak binning, and peak filtering. Pixels were classified as “foreground” or “background” (i.e., located inside or outside the tissue section) by applying thresholding on their sums of intensities of selected tissue-specific ions. We excluded ions that had stronger signals in the background than in the foreground because they appeared to be noisy and not biologically informative. Raw H&E images were preprocessed using standard protocols, including tissue detection and color normalization (HistomicsTK 1.2.3 package [34]). Then, for the kidney experiments, the whole-slide image was uniformly split into an array of image tiles, and each tile had a size of 200 × 200 pixels. Notably, the pixel size of MSI was 200 times larger than that of the H&E image (100 μm/0.5 μm), so each image tile had the same physical size as an MSI pixel. For the tumor specimen, the size of an H&E image tile was set to 100 × 100 pixels, in line with the smaller MSI pixel size (50 × 50 μm^2^). Tiles containing a minimum tissue area no less than 90% were classified as “foreground” and others were “background.” For the whole kidney specimen, all foreground MSI pixels and H&E image tiles were used for the following analysis; for the tumor specimen, only the tumor area and its surroundings were used for the following analysis to reduce computational cost. All data processing and analysis programs were implemented using Python 3.7 unless otherwise specified.

#### Encoding an H&E image tile into an HF spectrum

“Features” are a series of measurable properties to fully characterize an object in a data set. For MSI data, it is rather straightforward that *m*/*z* values are the features sufficient to convey molecular information of each spatial pixel. However, how to construct features to encode the histology appearance of a H&E image is not as self-evident. Mormont et al. [31] thoroughly investigated the performance of different DCNN architectures as off-the-shelf feature extractors for a group of histology and cytology object classification tasks. All DCNNs were pretrained using the ImageNet database [30]. An inner layer (conv5-block32-concat) of DenseNet 201 [35] empirically achieved the highest average performance in the tasks and thus stood out as our extractor of choice to encode the stain color and morphology of a 2D H&E image tile into a set of informative HFs. To stay in keeping with ImageNet, our input tiles had to be resized to [224, 224, 3] through bilinear interpolation, and each color channel had to be centered to zero. They were then forward propagated through a sequence of densely connected convolutional layers, where about 8.6 Giga FLOPs of calculation took place. Eventually, the output arrays after the concatenation operation at the conv5_block32 layer of DenseNet201 were 2D globally average-pooled, Min-Max scaled, and reshaped as an HF spectrum of 1,920 variables. The HF spectra of all the tiles were formatted into a hyperspectral data cube like MSI (Fig. 1): each tile became a pixel, and each (foreground) pixel had an HF spectrum. Image processing was implemented by OpenCV 4.2.0.32, and the pretrained DCNN was downloaded from Keras 2.4.3.

#### Spatial registration between MSI and HF data

**Figure 2:**
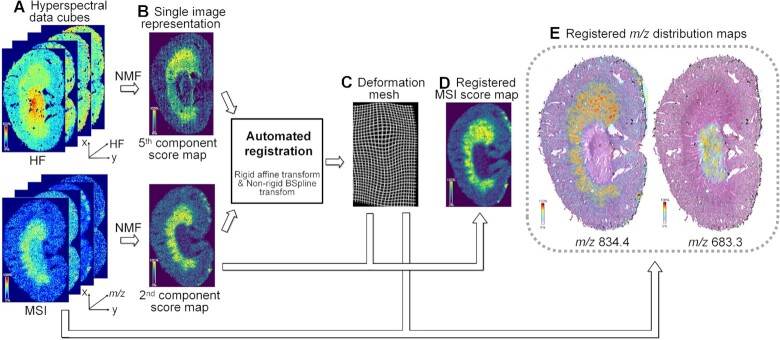
Multimodal registration between HF and MSI data. (A) The hyperspectral data cubes of HF and MSI. (B) The score maps of the fifth and second NMF components of the HF and MSI data, which were seen as their respective single-image representations and used as inputs to registration algorithms. (C) Deformation mesh to visualize the spatial transform output by registration algorithms. (D) The MSI score map aligned to the HF data cube by the above transform. (E) Ion maps of all *m*/*z* that were registered, upsampled, and overlapped on top of the H&E image.

Registration was a prerequisite to fusing multimodal imaging data sets, where the MSI and HF data cubes were spatially aligned. We used a dimension reduction algorithm, NMF, to generate single-image representations for the data cubes (as shown in Fig. 2): (i) NMF reduced the data cubes into 2 sets of score maps, and (ii) we manually selected 2 score maps whose spatial expression best matched each other (see Supplementary Fig. S3) and used them as the inputs of following intensity-based registration algorithms. DESI-MSI preferred a section thickness of >20 μm to produce intense ion signals, which was above the recommended thickness for H&E staining microscopy (<10 μm). So, we had to use serial sections, one for MSI and one for H&E. This meant that there might be nonlinear deformation between the MSI and HF score maps due to intersection anatomical variations or inconsistent sample preparation artifacts. Hence, we combined a linear registration algorithm (affine transform) with a nonlinear one (BSpline-based free form deformation) to both align the score maps globally and correct local deformations. Mutual information was used as the image similarity metric to guide the gradient descent-based optimization of linear and nonlinear transforms. Finally, we applied the optimized transforms to all ion maps (*m*/*z* channels). To evaluate registration quality, 2 representatives of registered ion maps (*m*/*z* 834.4 and 683.3) were upsampled 200 times (spline interpolation of 3 order) and overlaid on top of the H&E image in Fig. 2E. Supplementary Fig. S12 shows a visual assessment of the registration quality: good overlap between the HF-derived and MSI-derived tissue masks, as well as between the distinct anatomical features observed in both HF and MSI images, indicates good registration results. More about the registration process is in the supplementary material. Scikit-learn 0.24.2, Scikit-image 0.18.1, and SimpleITK 2.1.0 libraries were used to implement the above algorithms.

#### Segmentation and multimodal fusion-based evaluation

Spectral clustering is a graph-based clustering algorithm reported to outperform traditional algorithms such as k-means in MSI data-based tissue segmentation [36]. So, we used spectral clustering to group foreground pixels based on their HF and mass spectral similarities, respectively (more details in the supplementary material), and obtained 2 sets of clustering/segmentation results (i.e., 2 sets of labels for each pixel). Then, CKS [37] was employed to compare the 2 sets of results: perfect agreement gave a CKS of 1.0 and random labeling gave a CKS close to 0.0. The mathematical definition of CKS is given in the supplementary material. By taking into account the possibility of the agreement occurring by chance, CKS is not biased toward small #Clusters and hence a better measure than simple percentage agreement calculation. Note that the labeling of the clusters/regions between the 2 modalities was aligned by finding the largest CKS of all possible permutations. So, for a given #Clusters, CKS evaluated the biological validity of MSI segmentation by how much it was reproduced by histology segmentation. Supplementary Fig. S1 provides a step-by-step illustration of the entire methodology.

## Results and Discussion

### Spatial segmentation based on MSI and HF data

The kidney specimen was segmented into different regions along the image domain by clustering the MSI or HF data cubes on the basis of the spectral domain. The results are shown in Supplementary Fig. S4 for different #Clusters. As #Clusters became larger, finer anatomical details were increasingly revealed by both modalities until highly fragmented and scattered regions started to appear at larger #Clusters (≥6), which probably originated from modality-specific and instrumental noise/artifacts. Supplementary Fig. S5A shows the 3-dimensional (3D) embedding of all the 1920D HF spectra extracted from the H&E image tiles (here the embedding was realized by UMAP-based [38] dimensionality reduction). To sanity check whether segmentation based on HF was indeed histomorphology guided, we set #Clusters to 4 and retrieved 4 representative tiles that corresponded to the 4 data points located at the centers of each cluster (Supplementary Fig. S5A). Clearly, the 4 representative tiles had distinct histological appearances, which was in line with our expectations.

### Determining #Clusters

#Clusters is the most important parameter for the spectral clustering algorithm, which directly determines the granularity of ROIs used in downstream statistical analysis and may have a major impact on final scientific findings. To select the most probable #Clusters for the kidney specimen, the MSI segmentation results using different #Clusters had to be compared by certain clustering validation criteria. We first employed 2 internal validation measures proposed previously in literature [15, 21]. In [21], segmentation was evaluated by how closely it resembled the low-dimension overview of the high-dimensional molecular content of MSI data obtained by nonlinear dimension reduction techniques (here we used UMAP, as recommended in [39]). The resemblance between the UMAP image and the segmentation maps was measured by applying a Canny edge detector to both and computing their edge correlation. As shown by the red dashed line in Fig. 3, a higher Pearson correlation (PC) suggested higher resemblance, and the maximum was at #Clusters = 3. It appeared that the PC had relatively large error bars (standard deviation of PC values produced by 5 runs). This was because the specific choices of false color for each region appeared to considerably affect the Canny edges detected from the segmentation maps, and in our experiment, RGB color codes were randomly generated for each region in each run. In [15], the Davies–Bouldin index (DBI) was used to find the optimal segmentation for MSI data. DBI measures the ratio of within-cluster distances (the measure of intracluster compactness) to between-cluster distances (the measure of intercluster separation), so a smaller DBI indicates better-defined clusters and thus supposedly better segmentation. The DBI method suggested #Clusters = 2 as the optimal (the blue dotted line in Fig. 3). Both PC and DBI methods used internal information provided by the MSI data alone, so they could be vulnerable to instrumental noise or biologically irrelevant variations caused by experimental artifacts. It was also observed that different internal criteria might rank the goodness of the same clustering results in distinct ways. Such inconsistency was not surprising since those 2 criteria were designed based on very different rationales, but it might arouse concerns about the reproducibility and reliability of these internal criteria.

**Figure 3:**
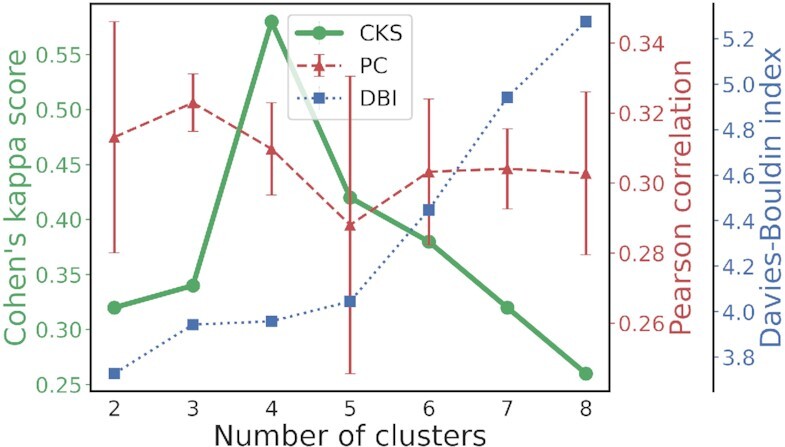
Determining the most probable #Clusters for the mouse kidney MSI data by (red) the PC-based method, (blue) the DBI-based method, and (green) our CKS-based strategy. CKS, PC, and DBI values were calculated for #Clusters = 2 to #Clusters = 8, and the error bars of PC were the standard deviations of 5 runs with random colors to label the regions. Both PC and DBI are so-called internal validation criteria, which suggested contradictory #clusters values: #Clusters = 3 and 2, respectively. Our CKS is an external validation criterion that used the histology segmentation as a reference and chose #Clusters = 4 for the good multimodal consistency it resulted in.

**Figure 4:**
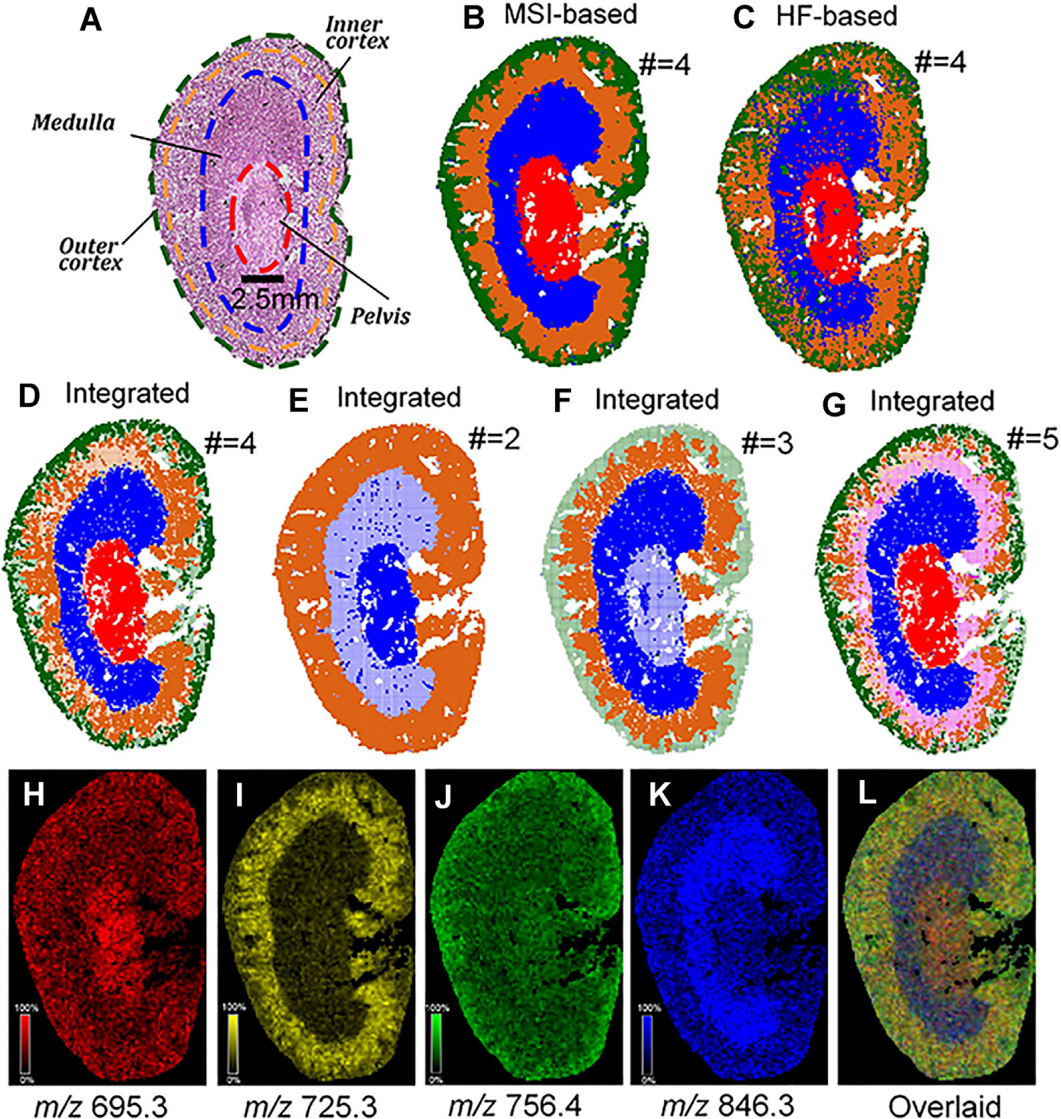
Tissue segmentation maps of a mouse kidney specimen: (A) the H&E image and anatomical structure, (B) MSI segmentation for #Clusters = 4, (C) histology segmentation for #Clusters = 4, and integrated maps for (D) #Clusters = 4, (E) #Clusters = 2, (F) #Clusters = 3, and (G) #Clusters = 5. In the integrated maps, a pixel had a solid color if it was identically labeled by both the MSI and histology segmentation; otherwise, it would be half-transparent. It was clear that when #Clusters = 4, the regions got almost reproduced by the 2 independent bioimaging modalities, which strongly supported their validity. (H–K) The characteristic *m*/*z* maps of each ROI, which are overlaid in (L).

In our multimodal fusion-based strategy, we generated another sequence (#Clusters = 2 to 8) of segmentation results by clustering the HF spectra. Cohen’s kappa score (CKS) between each pair of MSI and histology segmentation/clusterings was calculated and plotted as the green solid line in Fig. 3. More details as to CKS are provided in the Materials and Methods section and supplementary material. The maximum CKS was obtained with #Clusters = 4. In other words, setting #Clusters to 4 gave rise to better consistency between the segmentation independently produced by 2 distinct bioimaging modalities, indicating better multimodally corroborated biological validity. This can be further supported by Supplementary Fig. S5B, where 4 relatively well-segregated groups of data points were observed in the 3D UMAP embedding space of MSI data. Above all, #Clusters = 4 was in good accordance with the ground truth of renal anatomy (as shown in Fig. 4A): the 4 regions corresponded to the inner cortex, outer cortex, medulla, and pelvis, respectively. Our strategy is essentially an external validation criterion [22]: it integrates the bioinformation from both molecular profiles and the histomorphological appearance of a tissue specimen, which makes it immune to the noise and artifacts that are unlikely to exist in both MSI and histology data and thus provides a more objective and reliable approach to clustering/segmentation validation.

### Integrated tissue segmentation

MSI and histology segmentation maps of the kidney specimen (Fig. 4A) are shown in Fig. 4B and C, respectively. They were then integrated (Fig. 4D) to produce a more informative and reliable ROI for the tissue section: (i) each foreground pixel was assigned a false color according to its MSI-based cluster, and (ii) for any pixel whose MSI- and HF-based cluster labels were inconsistent, its transparency was set to 20%. The holes in the middle right portion of the tissue section were the renal vein and artery. By marking those pixels that were labeled differently by different imaging modalities, we could visualize the confidence levels across the tissue segmentation: solid color indicated that the 2 modalities had consensus, so we could be more confident about the labeling, whereas transparent pixels indicated that the 2 modalities had dissensus, so we had to be cautious about the labeling. In Fig. 4, integrated segmentation maps are shown for #Clusters equal to (D) 4, (E) 2, (F) 3, and (G) 5. In Fig. 3B, pixels whose cluster label = 1 were assigned to red, label = 2 to blue, label = 3 to orange, and label = 4 to green. In Fig. 3G, magenta was added to represent cluster label = 5. In line with Fig. 3, #Clusters = 4 resulted in the lowest proportion of inconsistently labeled (i.e., unconfident) pixels, suggesting regions best reproduced multimodally. Eventually, the 4 groups of confident pixels in Fig. 3D were returned as the ROIs of the whole kidney specimen, which related the spatial heterogeneity of chemical composition (detected by MSI) to that of histological morphology (detected by the H&E image).

A closer look at those unconfident pixels in Fig. 4D revealed that they very often appeared at the boundaries between the ROIs. There are 2 possible explanations for this: (i) each MSI pixel or H&E image tile had a size of 100 × 100 μm^2^, which made it sort of a “mixture” of cells from 2 neighboring tissue regions and therefore harder to be segmented correctly, and (ii) the registration between MSI and HF data might fail to fully correct all global and local differences between the serial tissues, so the one-to-one mapping between MSI pixel and H&E tile was not perfectly accurate (for instance, the pelvis of the H&E staining tissue section might be slightly smaller than that of the MSI section, and consequently, the peripheral part of the red region was labeled differently by Fig. 3B and C).

Moreover, as shown in Fig. 4B, a number of red pixels (i.e., pelvis) appeared to be scattered in the blue and orange regions (i.e., medulla and inner cortex). Given the fact that such a layout was anatomically impossible, it was probably due to the so-called “salt and pepper noise” common to MSI data or to certain artifacts incurred by the postacquisition data processing and clustering analysis pipeline. Such incorrectly labeled pixels could be difficult to notice for specimens without well-known histoanatomical structures. But in Fig. 4D, the integrated segmentation map labeled the scattered red pixels as unconfident, which suggested that our multimodal strategy was able to detect such histomorphologically baseless labeling. Conventionally, MSI users resorted to spatial-aware clustering approaches to handle such noise [13, 14]. Our strategy detected those misclassified pixels automatically and eliminated their potential negative influence on the following analysis by excluding them from ROI delineation. Conversely, the spatial-aware approaches would force them to be assigned to a seemingly correct ROI and propagate their noise to the following analysis, such as the calculation of the mean mass spectrum for a histology entity.

According to the integrated segmentation maps in Fig. 4D–G, it was the different orders of merging regions that led to the lower CKS at #Clusters = 2 and 3 in Fig. 3. For the MSI segmentation, the medulla was merged with the pelvis at #Clusters = 2 and 3. This was different from the histology segmentation, which instead merged the medulla to the cortex at #Clusters = 2 and defined it as a separate region at #Clusters = 3. At #Clusters = 4, which coincided with the intrinsic number of major anatomical structures of a mouse kidney, both the MSI and histology segmentation partitioned the tissue in a way matching up to those anatomical structures and therefore produced the highest CKS. When #Clusters = 5, the magenta segment discovered by MSI had no counterpart in the histology segmentation, suggesting that it could not be validated by histomorphology.

ROIs were defined using only the pixels of solid colors (i.e., confidently labeled pixels). Ions colocalized with each ROI were found by calculating the Pearson correlation between their spatial distributions and the binary mask of an ROI. As shown in Fig. 4H–K as well as in Supplementary Fig. S6, 695.3 *m*/*z*, 725.3 *m*/*z*, 756.4 *m*/*z*, and 846.3 *m*/*z* are presented as characteristic ions for each ROI, respectively, which were tentatively identified as [M+NH_4_]^+^ of PC(28:0), [M+Na]^+^ of SM(d34:1), [M+H]^+^ of PC(34:3), and [M+K]^+^ of PC(38:5) according to an organ-specific metabolite database built by Zhu et al. [40]. Figure 4L shows an overlay of H to K: areas dominated by the 4 ions were reasonably well separated and could largely account for the segmentation map (D). In Supplementary Fig. S7, our automated ROI delineation strategy was applied to another adjacent kidney tissue slice of the same specimen, which produced similar results and thus confirmed its reproducibility and robustness.

### Application on kidney tumor tissue

**Figure 5:**
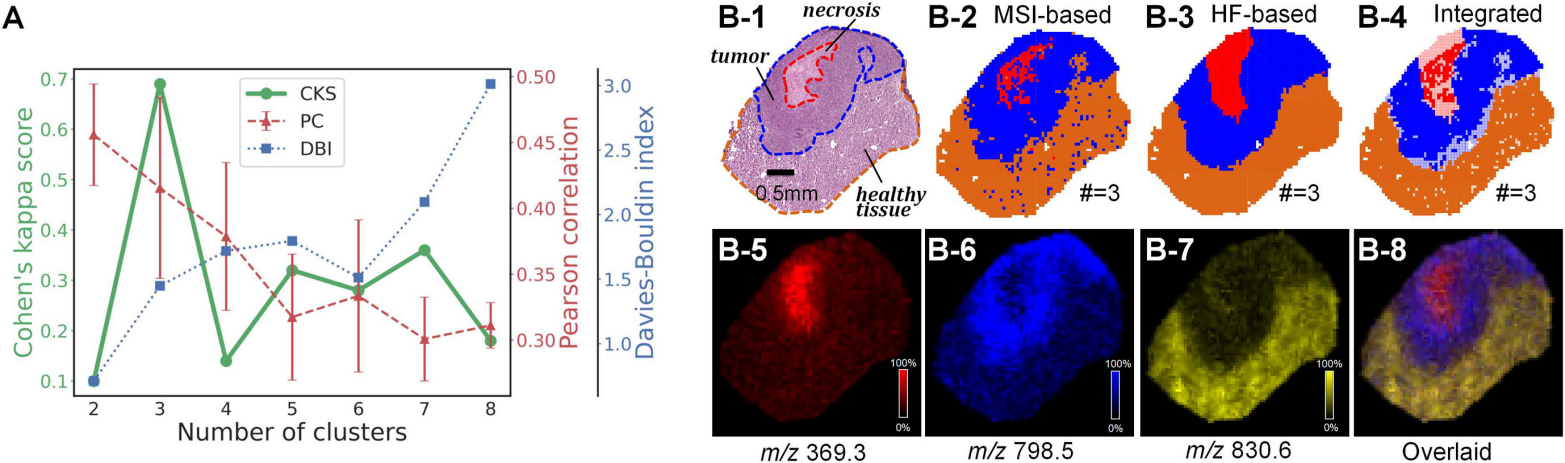
Application of our strategy on a renal adenocarcinoma tissue specimen from an orthotopic murine model: (A) CKS, PC, and DBI curves to determine the most probable #Clusters, (B-1) the H&E image and pathological structures, (B-2) MSI segmentation for #Clusters = 3, (B-3) histology segmentation for #Clusters = 3, and (B-4) their integrated maps. Solid pixels in (B-4) were returned as automatically defined ROIs. (B-5) to (B-7) show the characteristic *m*/*z* maps of each ROI, which are overlaid in (B-8). The tissue section was harvested from the murine model’s renal cortex, which was invaded by kidney cancer cells (ACHN) injected below its renal capsule.

Tumor specimens are central to MSI-based preclinical researches [1]. To assess the suitability of our strategy to tumor specimens, we applied it to the renal cortex tissue of an orthotopic mouse model invaded by human renal adenocarcinomas [41]. Again, the pretrained DCNN was used to produce histology segmentation, and the CKS curve was used to select #Clusters (the solid green line in Fig. 5A suggests #Clusters = 3). Figure 5B sums up the results for the tumor specimen: the MSI (Fig. 5B-2) and histology (Fig. 5B-3) segmentation were integrated into (Fig. 5B-4), where consistently and inconsistently labeled pixels were indicated in the same way as in Fig. 4. In Fig. 5B-5 to -7, 369.3 *m*/*z*, 798.5 *m*/*z*, and 830.6 *m*/*z* were selected through Pearson correlation as the characteristic ions for the red, blue, and orange ROIs, respectively. It can be seen from Fig. 5B-8, which was an overlay of Fig. 5B-5 to -7, that area dominated by each characteristic ion coincided well with its corresponding ROI. In order to reveal the pathological nature of the 3 ROIs, we consulted a histologist: as shown in Fig. 5B-1, the red, blue, and orange areas were recognized as a tumor necrotic zone, viable tumor, and healthy tissue, respectively. Tumor necrosis referred to tumor cell death in the core regions of solid tumors due to an accumulation of toxic waste products and a lack of oxygen and nutrient supply [42]. The 3 discriminant *m*/*z* discovered above were tentatively assigned as [M+H-H_2_O]^+^ of cholesterol, [M+K]^+^ of PC(34:1), and [M+Na]^+^ of PC(38:5), respectively, which might be associated with possible metabolic/lipidomic alterations due to kidney tumor development.

By comparing the MSI and histology segmentation results, we became able to automatically and unbiasedly determine to which extent that molecular variation could be explained by underlying histomorphological variation. So there are 2 remarkable benefits of adopting our strategy during ROI delineation. (i) It can guide histologists’ annotation toward appropriate granularity (i.e., the level of anatomical details). For instance, if the histologist had only partitioned our renal tumor specimen into “tumor” and “non-tumor” (i.e., delineated only 2 ROIs as suggested by PC and DBI in Fig. 5A), then the tumor necrotic zone would have been labeled as “tumor.” Following the conventional histology-guided protocol, we would have lost the opportunity to investigate the molecular difference between viable and necrotic tumors. (ii) It can help lessen the burden of histologists because they only need to visually inspect a few H&E image excerpts from each HF-based region before confirming its pathological state (as in Supplementary Fig. S10). Otherwise, they would have to examine the large 2-dimensional (2D) whole-slide image (10,000 to 100,000 pixels in each dimension) in an exhaustive manner and draw boundaries carefully around each recognized ROI. The tumor is one of the principal application areas of MSI, so confirming the suitability of our method to tumor specimens supports its practical value in future applications.

## Conclusions

The present study is undertaken to develop an intelligent tool for the ROI delineation step of MSI data analysis and demonstrate its practical value with distinct types of tissue specimens. Our multimodal fusion-based strategy provides an objective way to evaluate the validity of segmentation results. So we can compare the results obtained with different #Clusters and choose an optimal one, which ensures that each ROI is orthogonally confirmed by both the molecular imaging modality and histology microscopy modality. Due to the unsupervised nature of the histology segmentation method we proposed, it requires no more training and is applicable to all sorts of tissues without any specimen-specific adaption (no matter the species, organs, or pathological status). Although it may still be necessary to consult with histology experts when interpreting the biological nature of those ROIs, our method goes a long way toward guiding the histologists’ annotation and reducing their workloads.

In addition, we also reported a multimodal registration method between the MSI and microscopy datasets (more in the Materials and Methods section), which implicitly used the HF data as a “virtual intermediate modality.” Although integration with microscopy has been a routine part of MSI data analysis, registration between them can be a dilemma because of their disparate image characteristics, as discussed in [43]: the MSI of the mouse kidney shows narrow, spatially specific images in kidney regions but the H&E shows broad staining across the kidney, and a combination of stain color and morphology is used to delineate regions. The HF data solved this problem by (i) encoding the stain color and morphology information of H&E into the HF data cube, (ii) extracting region-specific images from the HF data cube with nonnegative matrix factorization (NMF), and (iii) performing registration between the 2 manually matched region-specific images (i.e., single-image representations). So, the registration approach introduced in this article has the potential to become a generic method for MSI and microscopy, but more work needs to be done to explore this idea further.

Our strategy is in principle applicable to other MSI ionization technologies such as secondary ion mass spectroscopy (SIMS) or MALDI (note that the software tool we provide is coded in Python, so the data format output by MSI instruments must be Python compatible, e.g., imzML), but follow-up studies are required to test its genericity. In particular, MALDI and SIMS can achieve lateral resolutions greater than 10 μm, in which case we anticipate that a more powerful spatial registration approach compatible with the single cell scale will be required to establish accurate correspondence between the MSI pixel and H&E tile. Combining our method with high spatial resolution MSI data may further improve the granularity and accuracy of the spatial segmentation results. For example, the “unconfident pixels” at the borders between ROIs (Fig. 4D and Fig. 5B-4]) may be subdivided and “deconvolved,” resulting in their assignment to existing clusters or the creation of new clusters.

Finally, in our multimodal fusion strategy, other imaging modalities can be used instead of optical microscopy. As an alternative method to obtain a label-free distribution of molecular species within a sample, vibrational spectroscopic imaging techniques, such as Fourier transform infrared (FTIR) and Raman imaging, capture information about chemical bonds, making them complementary to MSI and great candidates for multimodal corroboration of spatial segmentation. Specifically, we can replace the HF spectra extracted from each tile with corresponding FTIR or Raman spectra, and the rest of our strategy remains essentially the same.

## Availability of Source Code and Requirements

Source codes from this work are freely accessible at https://github.com/guoang4github/ROIforMSI/ (Licence: GPL-3). Our software tool is also registered in the bio.tools (roiformsi) and SciCrunch (RRID:SCR_023275) databases. The computational workflow is also registered in workflowhub.eu [44].

## Data Availability

All the data generated in this study are publicly available. The MSI data have been deposited to the ProteomeXchange Consortium (http://proteomecentral.proteomexchange.org) via the iProX partner repository [45] with the dataset identifier PXD038876. Snapshots of our code and other data further supporting this work are openly available in the *GigaScience* repository, GigaDB [46].

## Additional Files

**Supplementary Fig. S1**. The workflow of multimodal fusion-based region-of-interest (ROI) delineation. (I) Data preprocessing: raw MSI data were preprocessed by standard protocols for mass spectra, including Total Ion Count (TIC) normalization, smoothing, peak picking, peak alignment, peak binning, and peak filtering. A pixel was removed as background if its sum of peak intensities of tissue-specific ions was below a threshold value. Raw H&E image underwent tissue detection and color normalization before being split into an array of small tiles with a physical size equal to that of an MSI pixel. A tile was removed as background if its percentage of tissue area was less than 90%. (II) Encoding H&E image tiles by histomorphological features (HFs): H&E tiles were processed and propagated through a DCNN (DenseNet 201)–based HF extractor. The outputs of a middle layer (conv5-block32-concat) of DenseNet 201 were reduced to HF spectra by global average pooling. HF spectra were formatted as a hyperspectral data cube with x,y positions and HF channels. (III) Spatial registration between MSI and H&E image: nonnegative matrix factorization (NMF) was used to reduce the dimensions of the MSI and HF data to a number of components. HF data had to be processed by feature-wise Min-Max scaling because NMF required positive data values. Representative NMF components were manually chosen for the MSI and HF data by finding the pair of components whose score maps visually matched each other. After being scaled to a similar intensity range, the representative score map of MSI data was spatially aligned to that of HF data using a combination of linear and nonlinear automatic registration algorithms (based on affine and bspline transforms, respectively). We applied the spatial transforms obtained above to every *m/z* channel of the MSI data cube and produced a registered data cube. (IV) Multimodal fusion-based tissue segmentation: spectral clustering algorithm was used to cluster the foreground pixels of the MSI and HF data cubes, respectively. Cross-modal consistencies between MSI- and HF-based clusterings were measured for varying #Clusters using Cohen’s kappa score (CKS) after labeling alignment. We chose the #Clusters that produced the highest CKS and used consistently labeled pixels as the final ROIs.

**Supplementary Fig. S2**. CKS dependence on #Clusters using HF extracted by the block8_3_ac layer of Inception Resnet V2 and integrated segmentation maps at #Clusters = 2 to 5. It appeared that ROI results generated using the Inception Resnet were very similar to the DenseNet, and the rank of cross-modal clustering/segmentation consistencies remained almost the same, suggesting that the specific choice of DCNN extractor had a negligible influence on the outcome of our strategy.

**Supplementary Fig. S3**. The score maps of different components obtained by decomposing the HF and MSI data of the kidney sample with NMF. The score maps of the fifth and second components (red dashed box) of the HF and MSI data were used as the fixed and moving images for the following registration algorithms.

**Supplementary Fig. S4**. The segmentation maps of HF and MSI data with different #Clusters.

**Supplementary Fig. S5**. Visualizing the clusterings of the HF spectra and mass spectra of the kidney sample. (A) The high dimensional HF spectra were embedded in a 3D space using the nonlinear dimension reduction method UMAP and colored according to the clusters they belonged to; 4 characteristic H&E image tiles are displayed, which correspond to the data points at the center of each cluster in the 3D UMAP space. (B) The 3D scatterplot of the UMAP embedding of the mass spectra. The mean spectra of each cluster are displayed.

**Supplementary Fig. S6**. Characteristic *m/z* variables associated with different kidney regions. (A–D) Boxplots for the ion abundances of 695.3 *m/z*, 725.3 *m/z*, 756.4 *m/z*, and 846.3 *m/z*, which colocalized with the red, orange, green, and blue ROIs, respectively.

**Supplementary Fig. S7**. Repeating our ROI delineation method on another adjacent mouse kidney section. CKS dependence on #Clusters suggested that the optimal #Clusters was 4, which was in line with the result described in Figure 4.

**Supplementary Fig. S8**. The score maps of different components obtained by decomposing the HF and MSI data of the tumor sample with NMF. The score maps of the second and seventh components (red dashed box) of the HF and MSI data were used as the fixed and moving images for the following registration algorithms.

**Supplementary Fig. S9**. Multimodal registration between the HF and MSI data of the renal tumor sample. (A) The hyperspectral data cubes of HF and MSI. (B) The score maps of the second and seventh NMF components of the HF and MSI data, which were seen as their respective single-image representations and used as inputs to automated registration algorithms. (C) Deformation mesh to visualize the spatial transform output by registration algorithms. (D) The MSI score map was aligned to the HF data cube by the above transform. (E) Ion maps of all *m/z* were registered, upsampled, and overlapped on top of the H&E image.

**Supplementary Fig. S10**. Visualizing the clusterings of the HF spectra and mass spectra of the tumor sample. (A) The high dimensional HF spectra were embedded in a 3D space using nonlinear dimension reduction method UMAP and colored according to the clusters they belonged to; 3 characteristic H&E image tiles are displayed, which correspond to the data points at the center of each cluster in the 3D UMAP space. (B) The 3D scatterplot of the UMAP embedding of the mass spectra. The mean spectra of each cluster are displayed.

**Supplementary Fig. S11**. Characteristic *m/z* variables associated with different tumor regions. (A–C) Boxplots for the ion abundances of 369.3 *m/z*, 798.5 *m/z*, and 830.6 *m/z*, which colocalized with red, blue, and orange ROIs, respectively.

**Supplementary Fig. S12**. The evaluation of registration quality. (A-1) Good overlap between the HF- and MSI-derived kidney tissue masks after registration. (A-2 to -4) There is good spatial alignment between the distinct anatomical features observable in both the H&E and MSI images of the kidney tissue. (B-1 to -4) The same for the tumor tissue sample.

## Abbreviations

CKS: Cohen’s kappa score; DBI: Davies–Bouldin index; DCNN: deep convolutional neural network; DESI: desorption electrospray ionization; FTIR: Fourier transform infrared; H&E: hemotoxylin and eosin; HF: histomorphological feature; MALDI: matrix-assisted laser desorption ionization; MSI: mass spectrometry imaging; NMF: nonnegative matrix factorization; PC: Pearson correlation; ROI: region of interest; SIMS: secondary ion mass spectroscopy; UMAP: uniform manifold approximation and projection.

## Ethical Approval

The animal experiments were approved by the Animal Care and Use Committee of Shenzhen Institute of Advanced Technology, Chinese Academy of Sciences.

## Competing interests

The authors declare that they have no competing interests.

## Funding

This study was financially supported by the National Natural Science Foundation of China (82127801 and 22076197), National Key R&D Program of China (2022YFF0705003), Shenzhen Science and Technology Innovation Commission (JCYJ20200109115405930, JCYJ20210324115811031), Guangdong Basic and Applied Basic Research Foundation (2020B1515120080, 2021A1515110096), the Innovation Program for Excellent Young Researchers of Shenzhen Institute of Advanced Technology, and the Open Research Program of Guangzhou Life Sciences Facility Center of the Chinese Academy of Sciences (GZQY202004).

## Authors’ Contributions

A.G., F.L., and Q.L. designed research; A.G. and Z.C. performed research; A.G. analyzed data; and A.G. and Q.L. wrote the paper.

## Supplementary Material

giad021_GIGA-D-22-00334_Original_Submission

giad021_GIGA-D-22-00334_Revision_1

giad021_GIGA-D-22-00334_Revision_2

giad021_Response_to_Reviewer_Comments_Original_Submission

giad021_Response_to_Reviewer_Comments_Revision_1

giad021_Reviewer_1_Report_Original_Submission Chris Armit -- 1/20/2023 Reviewed

giad021_Reviewer_1_Report_Revision_1 Chris Armit -- 2/26/2023 Reviewed

giad021_Reviewer_2_Report_Original_Submission Stefania Alexandra Iakab -- 2/5/2023 Reviewed

giad021_Reviewer_3_Report_Original_Submission Nathan Heath Patterson -- 2/6/2023 Reviewed

giad021_Supplemental_File

## References

[1] McDonnell LA, Heeren RMA. Imaging mass spectrometry. Mass Spect Rev. 2007;26(4):606−43. 10.1002/mas.20124 17471576

[2] Norris JL, Caprioli RM. Imaging mass spectrometry: a new tool for pathology in a molecular age. Prot Clin Appl. 2013;7(11–12):733–8. 10.1002/prca.201300055 PMC3919023 24178781

[3] Chaurand P, Schwartz SA, Caprioli RM. Imaging mass spectrometry: a new tool to investigate the spatial organization of peptides and proteins in mammalian tissue sections. Curr Opin Chem Biol. 2002;6(5):676–81. 12413553 10.1016/s1367-5931(02)00370-8

[4] Seeley EH, Caprioli RM. Molecular imaging of proteins in tissues by mass spectrometry. Proc Natl Acad Sci USA. 2008;105(47):18126–31. 18776051 10.1073/pnas.0801374105 PMC2587620

[5] Cole LM, Clench MR. Mass spectrometry imaging tools in oncology. Biom Med. 2015;9(9):863–8. 10.2217/bmm.15.61 26330284

[6] Schnackenberg LK, Thorn DA, Barnette D et al. MALDI imaging mass spectrometry: an emerging tool in neurology. Metabolic Brain Dis. 2021;37(1):105−21. 10.1007/s11011-021-00797-2 34347208

[7] Watrous JD, Dorrestein PC. Imaging mass spectrometry in microbiology. Nat Rev Microbiol. 2011;9(9):683–94. 21822293 10.1038/nrmicro2634 PMC3710447

[8] Nilsson A, Goodwin RJ, Shariatgorji M, et al. Mass spectrometry imaging in drug development. Anal Chem. 2015;87(3):1437–55. 25526173 10.1021/ac504734s

[9] Thomas A, Patterson NH, Marcinkiewicz MM et al. Histology-driven data mining of lipid signatures from multiple imaging mass spectrometry analyses: application to human colorectal cancer liver metastasis biopsies. Anal Chem. 2013;85(5):2860–6. 23347294 10.1021/ac3034294

[10] Verbeeck N, Caprioli RM, de Plas RV. Unsupervised machine learning for exploratory data analysis in imaging mass spectrometry. Mass Spect Rev. 2020;39(3):245−91. 10.1002/mas.21602 PMC7187435 31602691

[11] Mccombie G, Staab D, Stoeckli M et al. Spatial and spectral correlations in MALDI mass spectrometry images by clustering and multivariate analysis. Anal Chem. 2005;77(19):6118–24. 16194068 10.1021/ac051081q

[12] Deininger S, Ebert M, Futterer A, et al. MALDI imaging combined with hierarchical clustering as a new tool for the interpretation of complex human cancers. J Proteome Res. 2008;7(12):5230–6. 19367705 10.1021/pr8005777

[13] Alexandrov T, Becker M, Deininger S, et al. Spatial segmentation of imaging mass spectrometry data with edge-preserving image denoising and clustering. J Proteome Res. 2010;9(12):6535–46. 20954702 10.1021/pr100734z

[14] Kobarg JH . Efficient spatial segmentation of large imaging mass spectrometry datasets with spatially aware clustering. Bioinformatics. 2011;27(13):230–8. 10.1093/bioinformatics/btr246 PMC3117346 21685075

[15] Inglese P, Mckenzie JS, Mroz A et al. Deep learning and 3D-DESI imaging reveal the hidden metabolic heterogeneity of cancer. Chem Sci. 2017;8(5):3500–11. 28507724 10.1039/c6sc03738k PMC5418631

[16] Delcourt V, Franck J, Leblanc E et al. Combined mass spectrometry imaging and top-down microproteomics reveals evidence of a hidden proteome in ovarian cancer. EBioMedicine. 2017;21:55–64. 28629911 10.1016/j.ebiom.2017.06.001 PMC5514399

[17] Song X, He J, Pang X, et al. Virtual calibration quantitative mass spectrometry imaging for accurately mapping analytes across heterogenous biotissue. Anal Chem. 2019;91(4):2838–46. 30636407 10.1021/acs.analchem.8b04762

[18] Jones MA, Cho SH, Patterson NH, et al. Discovering new lipidomic features using cell type specific fluorophore expression to provide spatial and biological specificity in a multimodal workflow with MALDI imaging mass spectrometry. Anal Chem. 2020;92(10):7079–86. 32298091 10.1021/acs.analchem.0c00446 PMC7456589

[19] Taylor AJ, Dexter A, Bunch J. Exploring ion suppression in mass spectrometry imaging of a heterogeneous tissue. Anal Chem. 2018;90(9):5637–45. 29461803 10.1021/acs.analchem.7b05005

[20] Bemis KD, Harry A, Eberlin LS et al. Probabilistic segmentation of mass spectrometry images helps select important ions and characterize confidence in the resulting segments. Mol Cell Proteomics Mcp. 2016;15(5):mcp.O115.053918. 10.1074/mcp.O115.053918 PMC4858953 26796117

[21] Abdelmoula WM, Balluff B, Englert S et al. Data-driven identification of prognostic tumor subpopulations using spatially mapped t-SNE of mass spectrometry imaging data. Proc Natl Acad Sci USA. 2016;113(43):12244–9. 27791011 10.1073/pnas.1510227113 PMC5087072

[22] Liu Y, Li Z, Xiong H et al. Understanding of internal clustering validation measures. In: 2010 IEEE International Conference on Data Mining. Webb G, Liu B, Zhang C, Gunopulos D, Wu X, Sydney, Australia: IEEE; 2010. p. 911–6.

[23] Moulavi D, Jaskowiak PA, Campello RJ, et al. Density-based clustering validation. In: Proceedings of the 2014 SIAM international conference on data mining. Zaki M, Obradovic Z, Tan P, Banerjee A, Kamath C, Parthasarathy S, Philadelphia, Pennsylvania, USA: SIAM; 2014. p. 839–47.

[24] Neumann EK, Djambazova KV, Caprioli RM, et al. Multimodal imaging mass spectrometry: next generation molecular mapping in biology and medicine. J Am Soc Mass Spect. 2020;31(12):2401–15. 10.1021/jasms.0c00232 PMC9278956 32886506

[25] Van de Plas R, Yang J, Spraggins J, et al. Image fusion of mass spectrometry and microscopy: a multimodality paradigm for molecular tissue mapping. Nat Methods. 2015;12(4):366–72. 25707028 10.1038/nmeth.3296 PMC4382398

[26] Rappez L, Stadler M, Triana S et al. SpaceM reveals metabolic states of single cells. Nat Methods. 2021;18(7):799–805. 34226721 10.1038/s41592-021-01198-0 PMC7611214

[27] LeCun Y, Bengio Y, Hinton G. Deep learning. Nature. 2015;521(7553):436–44. 26017442 10.1038/nature14539

[28] Coudray N, Ocampo PS, Sakellaropoulos T, et al. Classification and mutation prediction from non–small cell lung cancer histopathology images using deep learning. Nat Med. 2018;24(10):1559–67. 30224757 10.1038/s41591-018-0177-5 PMC9847512

[29] Weiss K, Khoshgoftaar TM, Wang DD. A survey of transfer learning. J Big Data. 2016;3(1):9.

[30] Russakovsky O, Deng J, Su H, et al. ImageNet large scale visual recognition challenge. Int J Comput Vision. 2015;115:211–52.

[31] Mormont R, Geurts P, Maree R. Comparison of deep transfer learning strategies for digital pathology. In: 2018 IEEE/CVF Conference on Computer Vision and Pattern Recognition Workshops (CVPRW). Salt Lake City, Utath, USA: IEEE, 2018.

[32] Goodwin R, Bunch J, McGinnity D. Mass spectrometry imaging in oncology drug discovery. Adv Cancer Res. 2017;134:133–71. 28110649 10.1016/bs.acr.2016.11.005

[33] Bemis KD, Harry A, Eberlin LS et al. Cardinal: an R package for statistical analysis of mass spectrometry-based imaging experiments. Bioinformatics. 2015;31(14):2418−20. 25777525 10.1093/bioinformatics/btv146 PMC4495298

[34] Cooper L . HistomicsTK. *GitHub*. 2016. https://github.com/DigitalSlideArchive/HistomicsTK.

[35] Huang G, Liu Z, Van Der Maaten L, et al. Densely connected convolutional networks. In: Proceedings of the IEEE Conference on Computer Vision and Pattern Recognition. Honolulu, Hawaii, USA: IEEE, 2017, p. 4700–8.

[36] Dexter A, Race AM, Steven RT, et al. Two-phase and graph-based clustering methods for accurate and efficient segmentation of large mass spectrometry images. Anal Chem. 2017;89(21):11293–300. 28849641 10.1021/acs.analchem.7b01758

[37] McHugh ML . Interrater reliability: the kappa statistic. Biochem Medica. 2012.22(3):276−82. PMC3900052 23092060

[38] McInnes L, Healy J, Melville J. UMAP: uniform manifold approximation and projection for dimension reduction. arXiv preprint 2018; arXiv: 180203426. 10.48550/arXiv.1802.03426.

[39] Smets T, Verbeeck N, Claesen M et al. Evaluation of distance metrics and spatial autocorrelation in uniform manifold approximation and projection applied to mass spectrometry imaging data. Anal Chem. 2019;91(9):5706–14. 30986042 10.1021/acs.analchem.8b05827

[40] Zhu Y, Zang Q, Luo Z, et al. An organ-specific metabolite annotation approach for ambient mass spectrometry imaging reveals spatial metabolic alterations of a whole mouse body. Anal Chem. 2022;94(20):7286–94. 35548855 10.1021/acs.analchem.2c00557

[41] Motzer RJ, Bander NH Nanus DM. Renal-cell carcinoma. N Engl J Med. 1996;335:865–75. 8778606 10.1056/NEJM199609193351207

[42] Vakkila J, Lotze MT. Inflammation and necrosis promote tumour growth. Nat Rev Immun. 2004;4(8):641–8. 10.1038/nri1415 15286730

[43] Tuck M, Blanc L, Touti R, et al. Multimodal imaging based on vibrational spectroscopies and mass spectrometry imaging applied to biological tissue: a multiscale and multiomics review. Anal Chem. 2020;93(1):445–77. 33253546 10.1021/acs.analchem.0c04595

[44] Guo A, Chen Z, Li F, et al. Delineating regions-of-interest for mass spectrometry imaging by multimodally corroborated spatial segmentation. *WorkflowHub*. 2023. 10.48546/WORKFLOWHUB.WORKFLOW.437.1.

[45] Ma J, Chen T, Wu S et al. iProX: An integrated proteome resource. Nucleic Acids Res. 2019;47(D1):D1211–7. 30252093 10.1093/nar/gky869 PMC6323926

[46] Guo A, Chen Z, Li F et al. Supporting data for “Delineating Regions-of-Interest for Mass Spectrometry Imaging by Multimodally Corroborated Spatial Segmentation.”. GigaScience Database. 2023. 10.5524/102374. PMC10087011 37039115

