# Supplementary material for: Delineating regions of interest for mass spectrometry imaging by multimodally corroborated spatial segmentation
Source: Gigascience. 2023 Apr 11;12:giad021. doi: 10.1093/gigascience/giad021 (PMC10087011; doi:10.1093/gigascience/giad021)
Supplement: giad021_GIGA-D-22-00334_Revision_1 [file giad021_giga-d-22-00334_revision_1.pdf]

GigaScience

Delineating Regions-of-interest for Mass Spectrometry Imaging by Multimodally Corroborated Spatial Segmentation

--Manuscript Draft--

**Manuscript Number:**	GIGA-D-22-00334R1	
**Full Title:**	Delineating Regions-of-interest for Mass Spectrometry Imaging by Multimodally Corroborated Spatial Segmentation	
**Article Type:**	Research	
**Funding Information:**	the National Natural Science Foundation of China (82127801)	Prof qian luo
	the National Natural Science Foundation of China (22076197)	Prof qian luo
	the Scientific Instrument Developing Project of the Chinese Academy of Sciences (YJKYYQ20200034)	Prof qian luo
	Shenzhen Engineering Laboratory of Single-molecule Detection and Instrument Development (XMHT20190204002)	Prof qian luo
	Shenzhen Science and Technology Innovation Commission (JCYJ20200109115405930)	Prof qian luo
	Basic and Applied Basic Research Foundation of Guangdong Province (JCYJ20210324115811031)	Prof qian luo
	Basic and Applied Basic Research Foundation of Guangdong Province (JCYJ20200109115405930)	Prof qian luo
	Basic and Applied Basic Research Foundation of Guangdong Province (2021A1515110096)	Dr Ang Guo
	Shenzhen Institutes of Advanced Technology Innovation Program for Excellent Young Researchers	Dr Ang Guo
	Guangzhou Life Sciences Facility Center of the Chinese Academy of Sciences Open Research Program (GZQY202004)	Dr Ang Guo

Abstract:

Mass spectrometry imaging, which localizes molecules in a tag-free, spatially-resolved manner, is a powerful tool for the understanding of underlying biochemical mechanisms of biological phenomena. When analyzing MSI data, it is essential to delineate Regions-of-Interest (ROIs) that correspond to tissue areas of different anatomical or pathological labels. Spatial segmentation, obtained by clustering MSI pixels according to their mass spectral similarities, is a popular approach to automate ROI definition. However, how to select the number of clusters (#Clusters), which determines the granularity of segmentation, remains to be resolved, and an inappropriate #Clusters may lead to ROIs not biologically real. Here we report a multimodal fusion strategy to enable an objective and trustworthy selection of #Clusters by utilizing additional information from corresponding histology images. A Deep Learning-based algorithm is proposed to extract "histomorphological feature spectra" across an entire H\&E image. Clustering is then similarly performed to produce Histology-segmentation. Since ROIs originating from instrumental noise or artifacts wouldn't be reproduced cross-modally, the consistency between histology- and MSI-segmentation becomes an effective measure of the biological validity of the results. So, #Clusters that maximizes the consistency is deemed as most probable. We validated our strategy on mouse kidney and renal tumor specimens by producing multimodally corroborated ROIs that agreed excellently with ground truths. Downstream analysis based on the said ROIs revealed lipid molecules highly specific to tissue anatomy or pathology. Our work will greatly facilitate MSI-mediated spatial lipidomics,

Powered by Editorial Manager® and ProduXion Manager® from Aries Systems Corporation

	metabolomics, and proteomics research by providing intelligent software to automatically and reliably generate ROIs.
**Corresponding Author:**	qian luo, Ph.D.<br>Shenzhen Institute of Advanced Technology, Chinese Academy of Sciences<br>Shenzhen, CHINA
**Corresponding Author Secondary Information:**	
**Corresponding Author's Institution:**	Shenzhen Institute of Advanced Technology, Chinese Academy of Sciences
**Corresponding Author's Secondary Institution:**	
**First Author:**	Ang Guo
**First Author Secondary Information:**	
**Order of Authors:**	Ang Guo
	Zhiyu Chen
	Fang Li
	qian luo, Ph.D.
**Order of Authors Secondary Information:**	
**Response to Reviewers:**	///In addition, please register any new software application in the bio.tools and SciCrunch.org databases to receive RRID (Research Resource Identification Initiative ID) and biotoolsID identifiers, and include these in your manuscript. Computational workflows should be registered in workflowhub.eu and the DOIs cited in the relevant places in the manuscript. These will facilitate tracking, reproducibility and re-use of your tool.///<br><br>Response:<br>RRID, biotoolsID identifiers, and workflowhub.eu DOI added (revised manuscript, lines 538-541).<br><br>///Please include a point-by-point within the 'Response to Reviewers' box in the submission system. Please ensure you describe additional experiments that were carried out and include a detailed rebuttal of any criticisms or requested revisions that you disagreed with. Please also ensure that your revised manuscript conforms to the journal style, which can be found in the Instructions for Authors on the journal homepage.<br><br>If the data and code has been modified in the revision process please be sure to update the public versions of this too.///<br><br>Response:<br>The public versions of the data and code have been updated.<br><br>///Reviewer reports:<br>Reviewer #1: In this interesting Research article, a Deep Learning-based algorithm is used to extract histomorphological DESI mass spectrometry imaging (MSI) feature spectra from H&E images. A key aspect of this approach is the use of a deep convolutional neural network (DCNN)-based feature extractor to generate spectra representative of microanatomical histomorphology in MSI pixels. Clustering is then used for spatial segmentation of regions of interest. As use case scenarios, the authors explored mouse kidney, and a mouse model of orthotopic human renal adenocarcinoma. Lipid dysregulation is a known pathological correlate in human renal adenocarcinoma, and the use of DESI mass spectrometry imaging enables spatial lipidomic analysis to be accomplished on H&E sections. The deep convolutional neural network (DCNN) approach utilised in this Research article is an interesting alternative to manual annotation, and the authors are to be commended for exploring automated alternatives to labour-intensive manual annotation.<br><br>Source code from this work are freely accessible from GitHub

(https://github.com/guoang4github/ROIforMSI/) where they have been ascribed an Open Source Initiative-approved GPL-3.0 Licence.

The MSI data include raw and processed MSI data in imzML format, and are available from the ProteomeXchange with accession identifier PXD03887. The TIF image files of mouse kidney and orthotopic human renal adenocarcinoma are additionally included in PXD03887.

Source code, MSI data, and H&E stained histological images used in this analysis have also been uploaded to GigaDB.

Major comment
In support of this manuscript, I will also require the following:
Regions of Interest (ROIs) generated by spatial segmentation, for example as binary masks associated with the H&E stained histological images. This invaluable output file would allow a researcher to overlay the ROIs, generated by spatial clustering, over the original H&E images. This is especially useful, and it will additionally allow researchers to assess pixelation in ROIs.///

Response:
We appreciate the reviewer's supportive comments, which are very helpful in improving the manuscript. Following the reviewer's suggestion, the ExampleWorkflow.ipyb files of our shared source code (both the files on the github and ftp servers) have been modified: the functionalities to output the ROI/label map generated by our strategy and to overlay it on the H&E image have been added. The ROI maps are stored as a 2D matrix where each element (i.e. pixel) records an integer corresponding to its assigned tissue category. The ROI maps of the kidney and tumour samples are uploaded to the FTP server ("kidneyMapOfROIs.npy" and "tumorMapOfROIs.npy").

///In addition, I have been informed that the mass spectrometry annotations are available from the DOME-ML Registry (https://registry.dome-ml.org/) with the following identifier:
d8887555-ed67-474f-a85d-798712d9ffa9
I cannot find this annotation on DOME-ML. Can you please send a link to the DOME-ML annotation?///

Response:
According to the DOME-ML Registry policy ("Submitted annotations will go through a manual revision process and will eventually be published. Published annotations will be made publicly available and will no longer be editable. Instead, unpublished annotations can only be viewed, edited or deleted by you."), our submission is probably still in the review process. I've tried to contact them to speed up the process, but haven't heard back yet. So I've uploaded a document ("DOME-Registry-Annotations.docx") of the annotations to the FTP server.

///Reviewer #2: The authors have presented in the current manuscript alternative data analysis tools which will benefit the mass spectrometry imaging community. Their focus was on determining the optimal number of regions of biological interest within tissues by multimodal registration and clustering. The authors underline the importance of unbiased segmentation of spectral data which also fits with biologically significant results.

The work carried out in the presented manuscript is highly useful within the context of mass spectrometry imaging data analysis. However, recent (MALDI) MS imaging datasets are nowadays collected with 20, 10 or even 5 um lateral resolutions, resulting in significantly increased number of pixels per tissue, and processing time, respectively. The authors claim that their tool can be used on any type of MSI data, independent of instrument or ionization techniques. This statement would be more convincing if the authors considered adding an example for their tool on one such dataset, or if the authors mentioned what is the required data format for using their tool (I assume imzML?). ///

Response:
We appreciate the reviewer's supportive comments and suggestions, which we found

very helpful. We acknowledge that we may have been overly optimistic about the generic nature of our strategy. So we toned down the statement and the required data format (imzML) has been mentioned.

Please see below reworded sentences.
“Our strategy is in principle applicable to other MSI ionization technologies such as Secondary Ion Mass Spectroscopy (SIMS) or MALDI (note that the software tool we provide is coded in Python, so the data format output by MSI instruments must be Python compatible, e.g. imzML), but follow-up studies are required to test its genericity.” (revised manuscript lines 373-378)

///Additionally, the authors should consider testing their tool on data collected with higher lateral resolution for MSI (e.g 10 um), because it would increase the possibility of associating "unconfident pixels" which appeared "at the boundaries between ROIs" to either existing clusters, or to generate new clusters. The resolution disparity between imaging modalities is too high in my opinion; H&E images enable distinguishing between cell types, but this information is lost because the image is down-sized to tiles which match the size of the MSI raster, thus containing information from several cells (but with no spatial differentiation) or even from morphological structures as glomeruli (~50um). Similarly, in the example of the tumor tissue, the inconclusive pixels, represented by transparent clusters in Figure 4 (b-4), might describe specific tissue types between healthy and cancer, and necrotic and tumor, respectively. I consider this a limitation that should be addressed by the authors.///

Response:
We agree with the reviewer that testing our method on higher resolution MSI data would strongly support the versatility of our strategy and may further improve the accuracy and granularity of the spatial segmentation results. However, there are three reasons why we cannot do this:
1.We currently don't have access to an MSI instrument that can achieve the lateral resolution of 10 um. The physical resolution limit of our common commercial DESI MSI instrument is about 35-50 um due to the size of the plume impact region (Analytical and bioanalytical chemistry, 2012, 404: 389-398.).
2.We've searched the common MSI data repositories (ProteomeXchange and PRIDE) and tried to find publicly available datasets with a spatial resolution of at least 10 um. However, it turned out that none of them included a high-resolution (>20X) H&E microscopy image, which is essential for the strategy proposed in our manuscript.
3.We anticipate that if we want to apply our method to MSI datasets with a resolution of 10 um, we would need a more powerful spatial registration approach capable of dealing with such high spatial resolution. Otherwise, the mismatch between the MSI pixel and the H&E tile due to misalignment could reduce the cross-modal consistency of the spatial segmentation, making our method less reliable. Spatial registration at the 10um (single cell) scale is still an active research area in the MSI community (Nature methods 2021, 18, 7 799. Analytical chemistry 2018, 90, 21 12395. Analytical chemistry 2018, 90, 21 12404) and is beyond the scope of this manuscript.

Of all the MSI datasets in the ProteomeXchange repository, more than 70% (17 out of 23) were acquired with a lateral resolution of worse than 50 um. The majority of current MSI studies are therefore dealing with data with a lateral resolution of worse than 50 um. In other words, even in the worst case that our method is not applicable to high lateral resolution MSI data, it is still useful for the majority of MSI studies, justifying its broad interest to the MSI community. Nevertheless, the reviewer's comment is very insightful and should certainly be taken into account in follow-up studies.

Please see below added sentences.
“In particular, MALDI and SIMS can achieve lateral resolutions greater than 10 um, in which case we anticipate that a more powerful spatial registration approach compatible with the single cell scale will be required to establish accurate correspondence between MSI pixel and H&E tile. Combining our strategy with high spatial resolution MSI data may further improve the granularity and accuracy of the spatial segmentation results. For example, the "unconfident pixels" at the borders between ROIs may be subdivided and "deconvolved", resulting in their assignment to existing clusters or the creation of new clusters.”(revised manuscript lines 378-387)

Powered by Editorial Manager® and ProduXion Manager® from Aries Systems Corporation

///Data presented in the manuscript are clearly described and the authors provide sufficient context for the reader to understand their potential uses. The authors also mention previous work based on the showcased data. I highly appreciate that the authors offer their tool freely, and that they provide the necessary information for all the data pre-processing as well as offer the readers example data for testing. For their tool to be widely used and appreciated by the community, I would suggest creating a user friendly interface for their software tool.///

Response:
Our group is currently working on a project to develop a more general software with non-programmer-friendly interfaces for multimodal data fusion of MSI, and the strategy proposed in this manuscript will be integrated into this tool.

///The authors have presented appropriate references to the literature, but they have omitted an important publication in the field [https://doi.org/10.1038/s41592-021-01198-0], which uses a similar concept for multimodal registration. ///

Response:
We thank the reviewer for the information on the paper, which is indeed very relevant to our study. A brief description of the paper has been added to the Introduction section.

Please see below the added sentence.
"For example, by integrating MALDI-MSI and microscopy, Rappez et al.[26] have developed a SpaceM method to characterize in situ single cell metabolomics."
(revised manuscript, lines 77-80).

///The methods section was well rounded, but one missing element was the information, tool, database or library they used for annotating several m/z values: e.g. lines 275-278, and lines 307-309.///

Response:
In original manuscript, lines 275-278 and lines 310-311 we selected 7 ions and tentatively identified them according to their mass-to-charge ratio (m/z). Searches of general metabolite/lipid databases, such as lipidmap, returned several possible matches. Unfortunately, without additional analytical information such as MS/MS, we couldn't determine which one was correct. To overcome this problem, we found a more specialised metabolite annotation database (Analytical Chemistry, 2022, 94(20): 7286-7294.) and used it as a reference. The database was built for DESI-MSI using mouse models in an organ-specific way. So, once we had chosen a suitable organ (kidney in our case), we could simply find the identified molecules in the database that might produce ions with an m/z comparable to the ions we had selected. We are now more confident about the identification results because the specialized database uses a similar ion source (i.e. DESI), the same animal models (i.e. mice), and the same organ (i.e. kidney), and it very often returns only a single match for an input m/z. The identification results (revised manuscript, lines 278-281 and lines 313-314) have been revised accordingly. A description of the database is added in the revised manuscript, line 282:
"...according to an organ-specific metabolite database built by Zhu et al [34].".

///The authors have strengthened their tool by using an orthogonal imaging method for reducing instrumental noise or acquisition artifacts, which I found very valuable as many research teams in the field already use staining protocols in their studies. The information obtained from stained tissues (H&E, IHC) is highly valuable for targeted studies, but it is limited for untargeted ones. Have the authors considered testing their tool on vibrational spectroscopy imaging data like FTIR or Raman? In this case the imaging can be done on the same section with increased ease for multimodal registration and minimal deformation between imaging modalities. Also, these techniques are providing additional, and orthogonal, molecular signatures.///

Response:
As the reviewer points out, other imaging modalities can be used instead of optical microscopy. As an alternative method to label-freely achieve the distribution of

Powered by Editorial Manager® and ProduXion Manager® from Aries Systems Corporation

molecular species within a sample, vibrational spectroscopic imaging techniques provide molecular information (signals due to vibrations from chemical bonds) complementary to MSI, making them great candidates for multimodal corroboration of spatial segmentation results. In principle, we can partition the tissue by clustering the FTIR or Raman spectra collected at each pixel and compare the spatial segmentation results obtained by vibrational spectroscopic imaging and MSI, respectively. The optical number of clusters can then be determined, just as we have done with the histomorphological feature spectra. But again, we do not currently have access to FTIR or Raman imaging instruments and cannot find suitable publicly available datasets. So we cannot test our method on the said datasets. Nevertheless, this comment is very instructive and we include it as a possible future direction in the conclusion section.

Please see below added sentences.
"Finally, in our multimodal fusion strategy, other imaging modalities can be used instead of optical microscopy. As an alternative method to obtain a label-free distribution of molecular species within a sample, vibrational spectroscopic imaging techniques, such as Fourier Transform Infrared (FTIR) and Raman imaging, capture information about chemical bonds, making them complementary to MSI and great candidates for multimodal corroboration of spatial segmentation. Specifically, we can replace the HF spectra extracted from each tile with corresponding FTIR or Raman spectra, and the rest of our strategy remains essentially the same." (revised manuscript, lines 388-397)

///The writing is clear, easy to follow, with minimal editing required: e.g. lines 182 "section and SI", and line 354 "more in section": there is a missing pointer to the section in each case.///

Response:
Pointers have been added.

///The data are presented in an appropriate manner, figures are representative of the described results, and almost ready to be published: Figure 2 should be prepared with the same font size as the other figures, and Figure 4 has a closed parenthesis ")" in the bottom right corner which should be removed.///

Response:
The font size has been enlarged and closed parenthesis has been removed.

///Some considerations:
I am missing "clean" optical or H&E images before multimodal registration (without the applied deformation mesh) to assess the quality of the tissue sections and the multimodal registration result. ///

Response:
H&E images have been added to Figure S12.

///Figure 5 (b) contains the only representation of the two tissue sections, where the MSI NMF score map seems to showcase a more intact tissue section, compared to the H&E score map, where the holes and tears in the tissue are visible. Could these missing pixels from the MSI data after registration omit some valuable information from the tissue? Figure S4 illustrates that the regions of the holes and tears are present in the MSI imaging data only after multimodal registration. In this context, how do you validate the registration of the two images?///

Response:
The original Figure 3(h-i) and 5(b) may be misleading. In fact, there are holes in the MSI tissue section (as shown in the tissue mask in Figure S12). The holes are not expected to be exactly the same, as serial tissue sections may be slightly different. When performing the spatial segmentation, only pixels/tiles marked as "tissue" by both the MSI and HF masks are used for the subsequent clustering analysis. In other words, the final tissue mask is the Hadamard product of the two binary masks. A large proportion of the holes correspond to the veins present in both tissue sections. The MSI signal in these areas therefore comes from the background glass slide and is

Powered by Editorial Manager® and ProduXion Manager® from Aries Systems Corporation

	therefore not informative. Indeed, there are MSI 'tissue' pixels that are mistakenly tagged as 'background' by the final mask, and the potentially valuable information they carry is omitted. However, compared to the total tissue, the proportion of mislabelled pixels is relatively small, so we don't expect that the information they carry would significantly change the results of the spatial segmentation and its evaluation based on cross-modal consistency.<br><br>Figure 3 (h-i) and 5 (b) are changed: the MSI tissue mask is applied to the ion maps.<br><br>Figure S12 is added to demonstrate the validation of the registration. The registration quality is visually inspected by the two approaches: (1) the overlap between the HF-derived and MSI-derived tissue masks after registration; (2) the spatial alignment between the distinguishable anatomical features observed in the H\&E and MSI images.<br><br>Please see below an added sentence.<br>“Figure.S12 shows a visual assessment of the registration quality: good overlap between the HF-derived and MSI-derived tissue masks, and between the distinct anatomical features observed in both HF and MSI images, indicate good registration results.” (revised manuscript, lines 509-513)<br><br>///On page 5 lines 312-315, the statement "By comparing the MSI- and histology-segmentation results, we became able to automatically and quantitatively determine to which extent that molecular variation could be explained by underlying histomorphological variation." gives the impression that obtaining the #clusters was also done in a quantitative manner, e.g. by multiple adjacent sections' analysis. Please consider rephrasing.///<br><br>Response:<br>“unbiasedly” is used instead of “quantitatively”. (revised manuscript, lines 317)<br><br><br>///Reviewer #3: GigaScience review<br><br>This is a well explained and demonstrated article for which I only have minor suggestions for improvement.///<br><br>Response:<br>We really appreciate the reviewer’s positive comments.<br><br>///Introduction comments<br>Authors state "…thus quantifying molecules in a spatially-resolved manner…". I think "localizing" molecules is a better very than quantifying since quantifying implies absolute quantitation in the field of mass spectrometry and quantifying endogenous proteins and metabolites by imaging MS is not routine. The authors are discussing the analysis of data through mathematical methods.///<br><br>Response:<br>“quantifying” is replaced with “localizing” (revised manuscript, first line in abstract and line 4)<br><br>///"homebrew algorithm" -> in-house algorithm///<br><br>Response:<br>"homebrew algorithm" is replaced with “in-house” (revised manuscript, first line in abstract and line 93)
**Additional Information:**	
**Question**	**Response**
Are you submitting this manuscript to a special series or article collection?	No
**Experimental design and statistics**	Yes

Full details of the experimental design and statistical methods used should be given in the Methods section, as detailed in our Minimum Standards Reporting Checklist. Information essential to interpreting the data presented should be made available in the figure legends.<br><br>Have you included all the information requested in your manuscript?	
**Resources**<br><br>A description of all resources used, including antibodies, cell lines, animals and software tools, with enough information to allow them to be uniquely identified, should be included in the Methods section. Authors are strongly encouraged to cite Research Resource Identifiers (RRIDs) for antibodies, model organisms and tools, where possible.<br><br>Have you included the information requested as detailed in our Minimum Standards Reporting Checklist?	Yes
**Availability of data and materials**<br><br>All datasets and code on which the conclusions of the paper rely must be either included in your submission or deposited in publicly available repositories (where available and ethically appropriate), referencing such data using a unique identifier in the references and in the "Availability of Data and Materials" section of your manuscript.<br><br>Have you have met the above requirement as detailed in our Minimum Standards Reporting Checklist?	Yes

Powered by Editorial Manager® and ProduXion Manager® from Aries Systems Corporation

Click here to access/download;LaTeX - Main Document (TeX file);main.tex

```
This is pdfTeX, Version 3.141592653-2.6-1.40.24 (TeX Live 2022)
(preloaded format=pdflatex 2022.11.21)  17 FEB 2023 04:21
entering extended mode
 restricted \write18 enabled.
 %&-line parsing enabled.
**main.tex
(./main.tex
LaTeX2e <2022-11-01>
L3 programming layer <2022-11-02>

! LaTeX Error: File `oup-contemporary.cls' not found.

Type X to quit or <RETURN> to proceed,
or enter new name. (Default extension: cls)

Enter file name:
! Emergency stop.
<read *>

l.11 ^^M

*** (cannot \read from terminal in nonstop modes)


Here is how much of TeX's memory you used:
 23 strings out of 474971
 503 string characters out of 5780120
 342064 words of memory out of 5000000
 21624 multiletter control sequences out of 15000+600000
 469259 words of font info for 28 fonts, out of 8000000 for 9000
 1141 hyphenation exceptions out of 8191
 19i,0n,29p,95b,17s stack positions out of
10000i,1000n,20000p,200000b,200000s
!  ==> Fatal error occurred, no output PDF file produced!
```

Manuscript

Click here to access/download;Manuscript;GigaScience_Journal_Manuscrip

GigaScience, 2017, 1–9

doi: xx.xxxx/xxxx
Manuscript in Preparation
Paper

PAPER

Delineating Regions-of-interest for Mass Spectrometry Imaging by Multimodally Corroborated Spatial Segmentation

Ang Guo[1], Zhiyu Chen[1,2], Fang Li[1] and Qian Luo[1,2,*]

[1]Institute of Biomedicine and Biotechnology, Shenzhen Institute of Advanced Technology, Chinese Academy of Sciences, Shenzhen, China. and [2]University of Chinese Academy of Sciences, Beijing, China.

[*]qian.luo@siat.ac.cn

Abstract

Mass spectrometry imaging, which localizes molecules in a tag-free, spatially-resolved manner, is a powerful tool for the understanding of underlying biochemical mechanisms of biological phenomena. When analyzing MSI data, it is essential to delineate Regions-of-Interest (ROIs) that correspond to tissue areas of different anatomical or pathological labels. Spatial segmentation, obtained by clustering MSI pixels according to their mass spectral similarities, is a popular approach to automate ROI definition. However, how to select the number of clusters (#Clusters), which determines the granularity of segmentation, remains to be resolved, and an inappropriate #Clusters may lead to ROIs not biologically real. Here we report a multimodal fusion strategy to enable an objective and trustworthy selection of #Clusters by utilizing additional information from corresponding histology images. A Deep Learning-based algorithm is proposed to extract "histomorphological feature spectra" across an entire H&E image. Clustering is then similarly performed to produce Histology-segmentation. Since ROIs originating from instrumental noise or artifacts wouldn't be reproduced cross-modally, the consistency between histology- and MSI-segmentation becomes an effective measure of the biological validity of the results. So, #Clusters that maximizes the consistency is deemed as most probable. We validated our strategy on mouse kidney and renal tumor specimens by producing multimodally corroborated ROIs that agreed excellently with ground truths. Downstream analysis based on the said ROIs revealed lipid molecules highly specific to tissue anatomy or pathology. Our work will greatly facilitate MSI-mediated spatial lipidomics, metabolomics, and proteomics research by providing intelligent software to automatically and reliably generate ROIs.

Key words: Mass spectrometry imaging; Spatial segmentation; Multimodal data fusion

Introduction

The alterations and interactions of biochemical pathways are often spatially-heterogeneous in a complex biological system, thus localizing molecules in a spatially-resolved manner is crucial for deciphering underlying biochemical mechanisms of biological phenomena. Mass Spectrometry Imaging (MSI) is a tag-free, high-throughput molecular mapping technique, which simultaneously acquires the spatial distributions of tens to hundreds of molecules by collecting a full mass spectrum in each pixel of a virtual grid[1, 2]. MSI can cover a wide variety of biomolecular species (including proteins, peptides, lipids, and metabolites) over biological samples with great sensitivity and chemical specificity [1, 2]. Since its emergence in the early 2000s[3, 4], MSI has enabled new biochemical discoveries in a wide range of life sciences including oncology[5], neurology[6], microbiology[7], and drug development[8]. During the post-acquisition analysis of MSI data, an intact tissue section is often virtually segmented into a number of Regions-of-Interest (ROIs) that correspond to different anatomical or pathological labels[1, 9]. An accurate definition of ROIs allows the extraction of tissue-type specific molecular abundances, which are essential for statistically discovering molecular alterations between different ROIs of the same specimen (for example, tumor versus

Compiled on: February 17, 2023.
Draft manuscript prepared by the author.

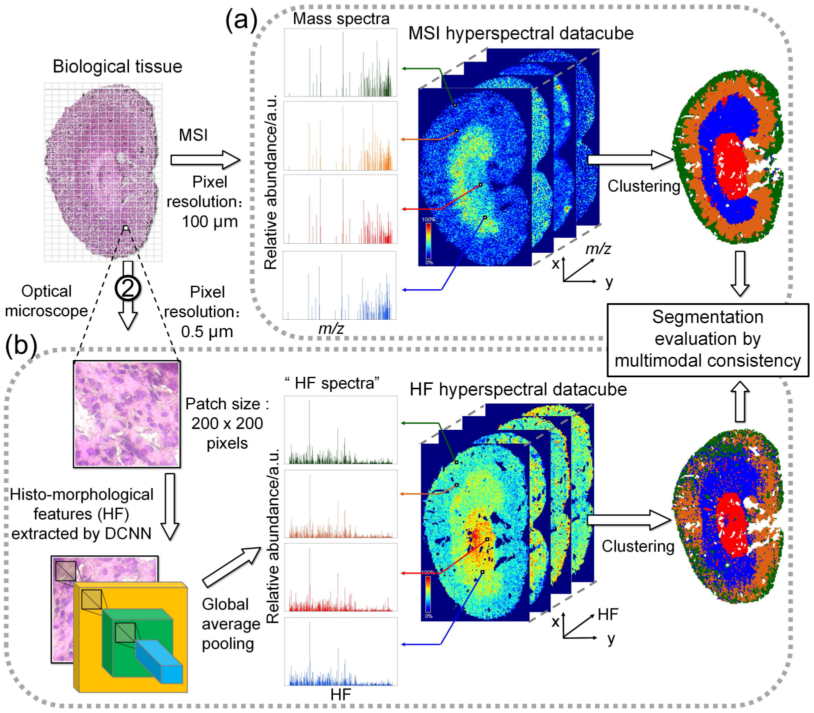

Figure 1. The overview of tissue segmentation based on (a) MSI data and (b) H&E stained histology image. In (a), mass spectra acquired by MSI can be formatted as a hyperspectral data cube. In (b), a high-resolution histology image was divided into an array of small tiles of size 200 $\times$ 200 pixels. A set of quantitative histomorphological features (HF) was then computed from each tile by a DCNN-based feature extractor. So another hyperspectral data cube similar to the MSI data was generated, with the only difference that the depth corresponded to HF rather than m/z. Clustering analysis in the spectral domain resulted in segmentation in the spatial domain. Segmentation/ROI validation was achieved by comparing the MSI- and histology-based results.

normal regions of a tissue section), or between different specimens at the same ROI (for example, differentially expressed molecules at a specific anatomical structure between diseased versus healthy samples).

There are two commonly-used methods for defining ROIs. The first is manual annotation guided by the staining microscopy of a tissue specimen section (or its consecutive slice)[9]. Manual annotation requires solid expert knowledge of histology and is prone to human bias. It can also be rather time-consuming when the sample size and/or the quantity of ROIs are large. Spatial segmentation [10] is the alternative approach for ROI definition, which is data-driven in nature and substantially automated. For a set of mass spectral profiles collected at different positions (i.e. pixels), a clustering algorithm separates them into several groups (i.e. clusters) in such a way that spectra in the same group are "similar" to each other than to those in other groups. Pixels of identical tissue types are expected to be similar in their chemical content and thus mass spectral profiles. So, by labeling each pixel with the color assigned to its cluster, we can obtain spatial segmentation along the image domain as shown in Figure 1(a). Following the pioneering work of Mccombie et al., the use of clustering analysis to discover geographically separated and molecularly distinct tissue regions from MSI data has been increasingly adopted by MSI community [12, 13, 14, 15, 16, 17, 18]. However, it remains a challenge to properly configure the parameters of clustering algorithms, which have a huge impact on final segmentation results. For instance, the number of clusters (#Clusters) determines the number of regions (i.e. the granularity of spatial segmentation) for algorithms such as K-Means and spectral clustering, but it is arbitrarily predefined by users. As #Clusters gets larger, the clustering algorithm would start to segment the specimen on the basis of instrumental noise or acquisition artifacts, producing ROIs that are not biologically real. Such difficulty in setting parameters is largely owing to the lack of a method for rigorously evaluating segmentation results. So far in the MSI community, segmentation has been evaluated either by subjective judgments [12, 13, 16, 17, 19] or by certain internal clustering validation criteria [20, 21, 15]. The internal clustering validation [22] evaluates the "goodness" of clustering based only on the intrinsic structure of the MSI data, making it still susceptible to the influence of noise or artifacts. Also, different internal validation criteria can be built based on completely different rationales and may give rise to inconsistent outcomes [23], which further reduces their reliability. Consequently, there is an urgent need for an objective and biologically-reliable approach to evaluating MSI-segmentation results and determining the key parameters of clustering algorithms.

Integrating the data acquired by two or more biomedical imaging modalities is termed multimodal fusion, which holds the promise of enhancing the discrimination between modality-specific instrumental noise or artifacts and biologically relevant chemical signals [24, 25]. Specifically, segmentation based on a modality orthogonal to MSI can provide an external reference to evaluate the results generated by MSI. Of all the different imaging modalities, light microscopy is one of the most ideal candidates because of its good accessibility to the MSI community. For example, by integrating MALDI-MSI and microscopy, Rappez et al.[26] have developed a SpaceM method to characterize in situ single cell metabolomics. In addition, the last decade has seen a phenomenal success of Deep Learning [27] in the analysis of histology microscopic images[28]. Based on a well-established idea called "transfer learning" [29], deep convolutional neural networks (DCNN) that pre-trained using source datasets ImageNet [30], appear to be excellent off-the-shelf feature extractors to represent histology images with histomorphologically informative features, which has been used to achieve accurate classification of cell or tissue types [31]. Therefore, it becomes technically feasible to calculate histology-based segmentation according to histomorphological features (HF) extracted from the histology image.

In this article, we report a multimodal fusion strategy between histology microscopy and MSI to enable an objective and trust-

worthy selection of #Clusters. First, an in-house algorithm was proposed to generate histology-segmentation from a H&E histology image. As shown in Figure 1, due to different lateral resolutions, each grid-scan pixel of MSI corresponds to a small tile of the whole slide H&E image of the same specimen. By propagating a tile through a DCNN-based feature extractor, we constructed an "HF spectrum" to represent the histomorphology of cellular neighborhoods located within an MSI pixel. Analogous to the MSI-segmentation, histology-segmentation was obtained by clustering the pixels according to their HF spectral similarities. Second, built upon an assumption that more reliable segmentation would be better replicated by orthogonal imaging modalities, we used the multi-modal consistency between histology- and MSI- segmentation as a quantitative measure to evaluate validity. So, different #Clusters were compared accordingly and the one that produced maximum consistency was deemed most probable. Last, using the optimal #Clusters, pixels concordantly labeled by the two modalities were returned as ROIs of biological relevance supported by both molecular and histomorphological profiling. In the following sections, we used whole mouse kidney and tumor specimens as proof-of-concept examples to validate our strategy: ROIs produced by our strategy were in excellent agreement with ground truths. Based on the said ROIs, downstream data analysis revealed lipids that were highly specific to tissue anatomy or pathology: for the kidney specimen, DG(42:4), SM(d34:1), PC(34:3), and PC(40:0) appeared to be colocalized with the pelvis, inner cortex, outer cortex, and medulla, respectively; for the tumor specimen, MG(16:0), PC(34:1), and PC(38:5) were colocalized with the necrosis, viable tumor, and normal tissues. We expect to facilitate MSI data analysis by offering an intelligent tool for ROI delineation that is biologically reliable and intrinsically immune to subjective bias.

Results and discussion

Spatial segmentation based on MSI and HF data

The kidney specimen was segmented into different regions along the image domain by clustering the MSI or HF data cubes on the basis of the spectral domain. The results are shown in Figure S4 for different #Clusters. As #Clusters became larger, finer anatomical details were increasingly revealed by both modalities until highly fragmented and scattered regions started to appear at larger #Clusters (>=6), which probably originated from modality-specific and instrumental noise/artifacts. Figure S5(a) shows the 3D embedding of all the 1920D HF spectra extracted from the H&E image tiles (here the embedding was realized by UMAP-based [32] dimensionality reduction). To sanity check whether segmentation based on HF was indeed histomorphology-guided, we set #Clusters to 4 and retrieved 4 representative tiles that corresponded to the 4 data points located at the centers of each cluster (Figure S5(a)). Clearly, the 4 representative tiles had distinct histological appearances, which was in line with our expectations.

Determining #Clusters

#Clusters is the most important parameter for the spectral clustering algorithm, which directly determines the granularity of ROIs used in downstream statistical analysis and may have a major impact on final scientific findings. To select the most probable #Clusters for the kidney specimen, the MSI-segmentation results using different #Clusters had to be compared by certain clustering validation criteria. We first employed two internal validation measures proposed previously in literature [21, 15]. In 21, segmentation was evaluated by how closely it resembled the low-dimension overview of the high-dimensional molecular content of MSI data obtained by nonlinear dimension reduction techniques (here we used UMAP as recommended in [33]). The resemblance between the UMAP image and the segmentation maps was measured by applying a Canny edge detector to both and computing their edge correlation. As shown by the red dashed line in Figure 2, a higher Pearson correlation (PC) suggested higher resemblance and the maximum was at #Clusters=3. It appeared that the PC had relatively large error bars (standard deviation of PC values produced by five runs). This was because the specific choices of false color for each region appeared to considerably affect the Canny edges detected from the segmentation maps and in our experiment RGB color codes were randomly generated for each region in each run. In 15, the Davies–Bouldin index (DBI) was used to find the optimal segmentation for MSI data. DBI measures the ratio of within-cluster distances (the measure of intra-cluster compactness) to between-cluster distances (the measure of inter-cluster separation), so a smaller DBI indicates better-defined clusters and thus supposedly better segmentation. The DBI method suggested #Clusters=2 as the optimal (the blue dotted line in Figure 2). Both PC and DBI methods used internal information provided by the MSI data alone, so they could be vulnerable to instrumental noise or biologically irrelevant variations caused by experimental artifacts. It was also observed that different internal criteria might rank the goodness of the same clustering results in distinct ways. Such inconsistency was not surprising since those two criteria were designed based on very different rationales, but it might arouse concerns about the reproducibility and reliability of these internal criteria.

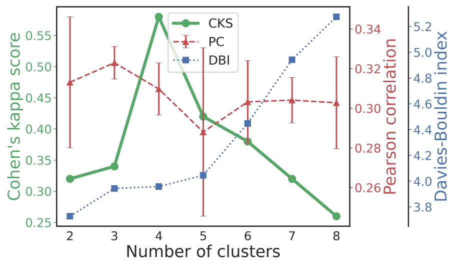

Figure 2. Determining the most probable #Clusters for the mouse kidney MSI data by (red) the PC-based method, (blue) the DBI-based method, and (green) our CKS-based strategy. CKS, PC, and DBI values were calculated for #Clusters=2 to #Clusters=8, and the error bars of PC were the standard deviations of five runs with random colors to label the regions. Both PC and DBI are so-called internal validation criteria, which suggested contradictory #clusters values: #Clusters=3 and 2 respectively. Our CKS is an external validation criterion that used the histology-segmentation as a reference and chose #Clusters=4 for the good multimodal consistency it resulted in.

In our multimodal fusion-based strategy, we generated another sequence (#Clusters=2 to 8) of segmentation results by clustering the HF spectra. Cohen's kappa score (CKS) between each pair of MSI- and histology-segmentation/clusterings were calculated and plotted as the green solid line in Figure 2. More details as to CKS in the Materials and Methods section and SI. The maximum CKS was obtained with #Clusters=4. In other words, setting #Clusters to 4 gave rise to better consistency between the segmentation independently produced by two distinct bio-imaging modalities, indicating better multimodally corroborated biological validity. This can be further supported by Figure S5(b), where four relatively well-segregated groups of data points were observed in the 3D UMAP embedding space of MSI data. Above all, #Clusters=4 was in good accordance with the ground truth of renal anatomy (as shown in Figure 3(a)): the four regions corresponded to the inner cortex, outer cortex, medulla, and pelvis, respectively. Our strategy is essentially an external validation criterion [22]: it integrates the bio-information from both molecular profiles and the histomorphological appearance of a tissue specimen, which makes it immune to the noise and artifacts that are unlikely to exist in both MSI and histology data, and thus provides a more objective and reliable approach to clustering/segmentation validation.

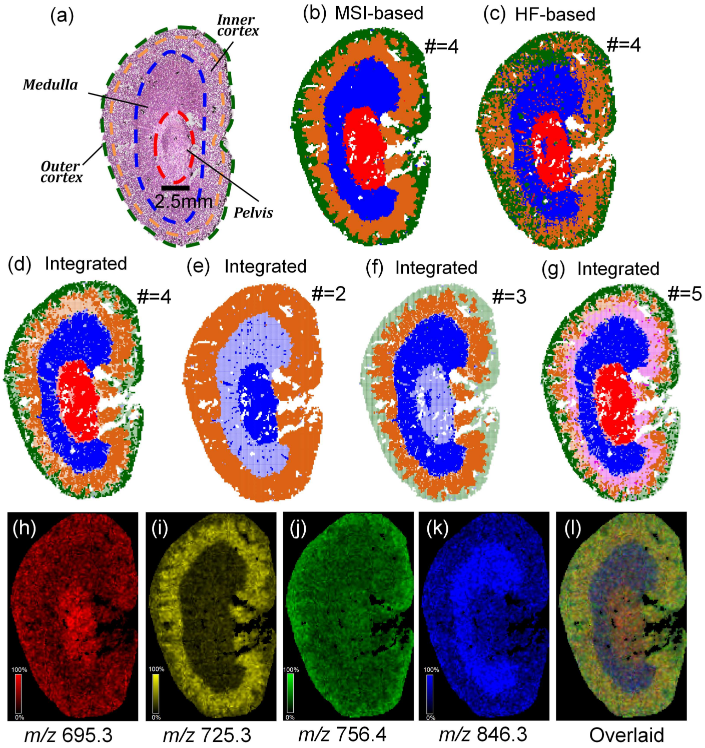

Figure 3. Tissue segmentation maps of a mouse kidney specimen: (a) the H&E image and anatomical structure, (b) MSI-segmentation for #Clusters=4, (c) histology-segmentation for #Clusters=4, and integrated maps for (d) #Clusters=4, (e) #Clusters=2, (f) #Clusters=3, (g) #Clusters=5. In the integrated maps, a pixel had a solid color if it was identically labeled by both the MSI- and histology-segmentation, otherwise, it would be half-transparent. It was clear that when #Clusters = 4, the regions got almost reproduced by the two independent bio-imaging modalities, which strongly supported their validity. (h)-(j) show the characteristic *m/z* maps of each ROI, which are overlaid in (k).

Integrated tissue segmentation

MSI- and histology-segmentation maps of the kidney specimen (Figure 3(a)) are shown in Figure 3(b) and (c) respectively. They were then integrated (Figure 3(d)) to produce a more informative and reliable ROI for the tissue section: (1) each foreground pixel was assigned a false color according to its MSI-based cluster; (2) for any pixel whose MSI- and HF-based cluster labels were inconsistent, its transparency was set to 20%. The holes in the middle right portion of the tissue section were the renal vein and artery. By marking those pixels that were labeled differently by different imaging modalities, we could visualize the confidence levels across the tissue segmentation: solid color indicated that the two modalities had consensus, so we could be more confident about the labeling, whereas transparent pixels indicated that the two modalities had dissensus, so we had to be cautious about the labeling. In Figure 3, integrated segmentation maps are shown for #Clusters equal to (d) 4, (e) 2, (f) 3, and (g) 5. In (b), pixels whose cluster label = 1 were assigned to red, label = 2 to blue, label = 3 to orange, and label = 4 to green. In (g), magenta was added to represent cluster label = 5. In line with Figure.2, #Clusters=4 resulted in the lowest proportion of inconsistently labeled (i.e. unconfident) pixels, suggesting regions best reproduced multimodally. Eventually, the 4 groups of confident pixels in (d) were returned as the ROIs of the whole kidney specimen, which related the spatial heterogeneity of chemical composition (detected by MSI) to that of histological morphology (detected by H&E image).

A closer look at those unconfident pixels in Figure 3(d) revealed that they very often appeared at the boundaries between the ROIs. There are two possible explanations for this: (1) each MSI pixel or H&E image tile had a size of 100×100 μm^2, which made it sort of a "mixture" of cells from two neighboring tissue regions and therefore harder to be segmented correctly; (2) the registration between MSI and HF data might fail to fully correct all global and local differences between the serial tissues, so the one-to-one mapping between MSI pixel and H&E tile was not perfectly accurate (for instance, the pelvis of the H&E staining tissue section might be slightly smaller than that of the MSI section and consequently the peripheral part of the red region was labeled differently by (b) and (c)).

Moreover, as shown in Figure 3(b), a number of red pixels (i.e. pelvis) appeared to be scattered in the blue and orange regions (i.e. medulla and inner cortex). Given the fact that such a layout was anatomically impossible, it was probably due to the so-called "salt and pepper noise" common to MSI data or due to certain artefacts incurred by the post-acquisition data processing and clustering analysis pipeline. Such incorrectly labeled pixels could be difficult to notice for specimens without well-known histoanatomical structures. But in Figure 3(d), the integrated segmentation map labeled the scattered red pixels as unconfident, which suggested that our multi-modal strategy was able to detect such histomorphologically-baseless labeling. Conventionally, MSI users resorted to spatial aware clustering approaches to handle such noise [13, 14]. Our strategy detected those misclassified pixels automatically and eliminated their potential negative influence on the following analysis by excluding them from ROI delineation. Conversely, the spatial aware approaches would force them to be assigned to a seemingly correct ROI and propagate their noise to the following analysis such as the calculation of the mean mass spectrum for a histology entity.

According to the integrated segmentation maps in Figure 3 (d-g), it was the different orders of merging regions that led to the lower CKS at #Clusters = 2 and 3 in Figure 2. For the MSI-segmentation, the medulla was merged with the pelvis at #Clusters = 2 and 3. This was different to the histology-segmentation which instead merged the medulla to the cortex at #Clusters = 2 and defined it as a separate region at #Clusters = 3. At #Clusters = 4, which coincided with the intrinsic number of major anatomical structures of a mouse kidney, both the MSI- and histology-segmentation par-

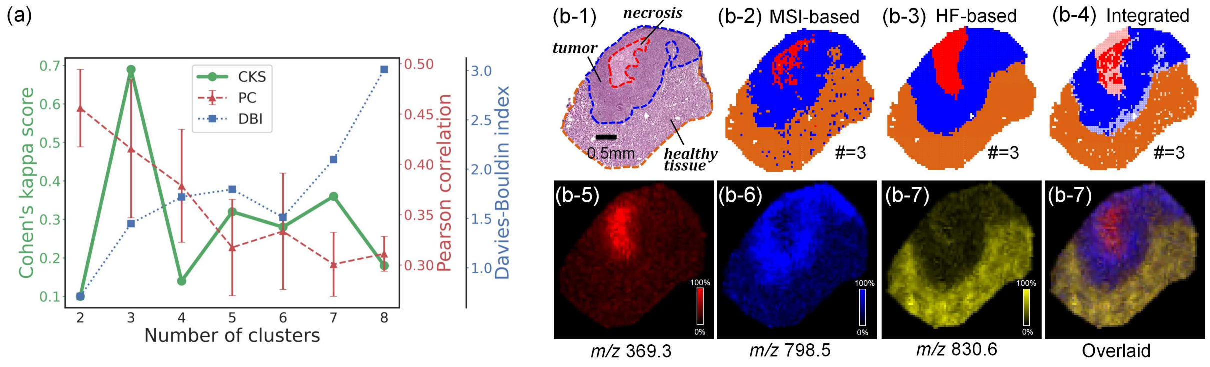

Figure 4. Application of our strategy on a renal adenocarcinoma tissue specimen from an orthotopic murine model: (a) CKS, PC, and PC curves to determine the most probable #Clusters, (b-1) the H&E image and pathological structures, (b-2) MSI-segmentation for #Clusters=3, (b-3) histology-segmentation for #Clusters=3, and (b-4) their integrated maps. Solid pixels in (b-4) were returned as automatically defined ROIs. (b-5)-(b-7) show the characteristic *m/z* maps of each ROI, which are overlaid in (b-8). The tissue section was harvested from the murine model's renal cortex, which was invaded by kidney cancer cells (ACHN) injected below its renal capsule.

titioned the tissue in a way matching up to those anatomical structures and therefore produced the highest CKS. When #Clusters=5, the magenta segment discovered by MSI had no counterpart in the histology-segmentation, suggesting that it could not be validated by histomorphology.

ROIs were defined using only the pixels of solid colors (i.e. confidently labeled pixels). Ions colocalized with each ROI were found by calculating the Pearson correlation between their spatial distributions and the binary mask of an ROI. As shown in Figure 3 (h) to (k) as well as in Figure S6, 695.3 *m/z*, 725.3 *m/z*, 756.4 *m/z*, and 846.3 *m/z* are presented as characteristic ions for each ROI respectively, which were tentatively identified as $[M+NH_4]^+$ of PC(28:0), $[M+Na]^+$ of SM(d34:1), $[M+H]^+$ of PC(34:3), and $[M+K]^+$ of PC(38:5) according to an organ-specific metabolite database built by Zhu et al[34]. Figure 3 (k) shows an overlay of (h) to (j): areas dominated by the four ions were reasonably well separated and could largely account for the segmentation map (d). In Figure S7, our automated ROI delineation strategy was applied to another adjacent kidney tissue slice of the same specimen, which produced similar results and thus confirmed its reproducibility and robustness.

Application on kidney tumor tissue

Tumor specimens are central to MSI-based preclinical researches[1]. To assess the suitability of our strategy to tumor specimens, we applied it to the renal cortex tissue of an orthotopic mouse model invaded by human renal adenocarcinomas[35]. Again, the pretrained DCNN was used to produce histology-segmentation and the CKS curve was used to select #Clusters (the solid green line in Figure 4 (a) suggests #Clusters=3). Figure 4 (b) sums up the results for the tumor specimen: the MSI- (b-2) and histology- (b-3) segmentation were integrated into (b-4), where consistently and inconsistently labeled pixels were indicated in the same way as Figure 3. In Figure 4 (b-5 to b-7), 369.3 *m/z*, 798.5 *m/z*, and 830.6 *m/z* were selected through Pearson correlation as the characteristic ions for the red, blue, and orange ROIs respectively. It can be seen from Figure 4 (b-8), which was an overlay of (b-5) to (b-7), that area dominated by each characteristic ion coincided well with its corresponding ROI. In order to reveal the pathological nature of the three ROIs, we consulted a histologist: as shown in Figure 4(b-1), the red, blue, and orange areas were recognized as tumor necrotic zone, viable tumor, and healthy tissue, respectively. Tumor necrosis referred to tumor cell death in the core regions of solid tumors due to an accumulation of toxic waste products and a lack of oxygen and nutrient supply[36]. The three discriminant *m/z* discovered above were tentatively assigned as $[M+H-H_2O]^+$ of Cholesterol, $[M+K]^+$ of PC(34:1), and $[M+Na]^+$ of PC(38:5) respectively, which might be associated with possible metabolic/lipidomic alterations due to kidney tumor development.

By comparing the MSI- and histology-segmentation results, we became able to automatically and unbiasedly determine to which extent that molecular variation could be explained by underlying histomorphological variation. So there are two remarkable benefits of adopting our strategy during ROI delineation. (1) It can guide histologists' annotation towards appropriate granularity (i.e. the level of anatomical details). For instance, if the histologist had only partitioned our renal tumor specimen into 'tumor' and 'non-tumor' (i.e. delineated only two ROIs as suggested by PC and DBI in Figure 4(a)), then the tumor necrotic zone would have been labeled as 'tumor'. Following the conventional histology-guided protocol, we would have lost the opportunity to investigate the molecular difference between viable and necrotic tumors. (2) It can help lessen the burden of histologists because they only need to visually inspect a few H&E image excerpts from each HF-based region before confirming its pathological state (as in Figure S10). Otherwise, they would have to examine the large 2D whole slide image (10,000 to 100,000 pixels in each dimension) in an exhaustive manner and draw boundaries carefully around each recognized ROI. The tumor is one of the principal application areas of MSI, so confirming the suitability of our method to tumor specimens supports its practical value in future applications.

Conclusions

The present study is undertaken to develop an intelligent tool for the ROI delineation step of MSI data analysis and demonstrate its practical value with distinct types of tissue specimens. Our multimodal fusion-based strategy provides an objective way to evaluate the validity of segmentation results. So we can compare the results obtained with different #Clusters and choose an optimal one, which assures that each ROI is orthogonally confirmed by both the molecular imaging modality and histology microscopy modality. Due to the unsupervised nature of the histology-segmentation method we proposed, it requires no more training and is applicable to all sorts of tissues without any specimen-specific adaption (no matter the species, organs, or pathological status). Although it may still be necessary to consult with histology experts when interpreting the biological nature of those ROIs, our method goes a long way towards guiding the histologists' annotation and reducing their workloads.

In addition, we also reported a multimodal registration method between the MSI and microscopy datasets (more in the Materials and Methods section), which implicitly used the HF data as a "virtual intermediate modality". Although integration with microscopy has been a routine part of MSI data analysis, registration between them can be a dilemma because of their disparate image

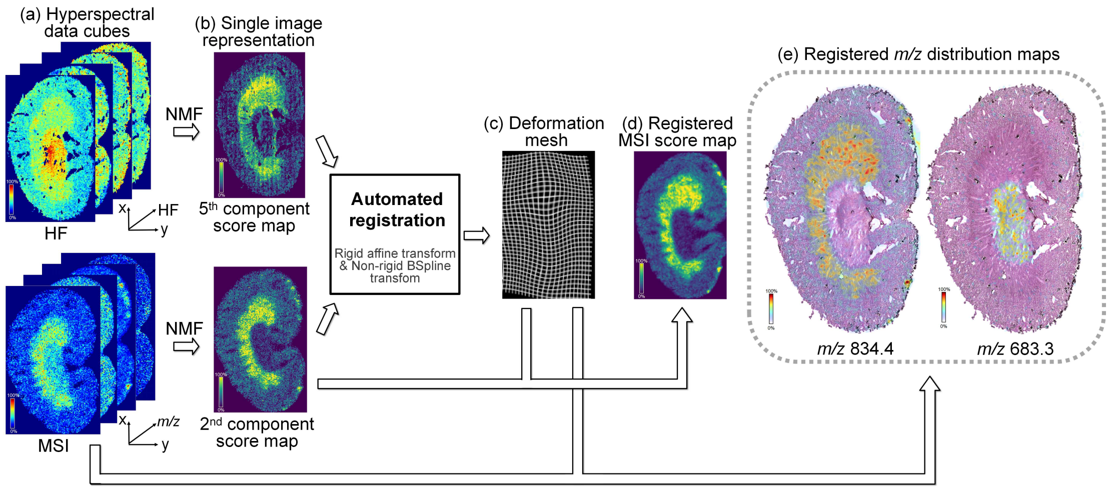

Figure 5. multimodal registration between HF and MSI data. (a) the hyperspectral data cubes of HF and MSI, (b) the score maps of the 5th and 2nd NMF components of the HF and MSI data, which were seen as their respective single image representations and used as inputs to registration algorithms, (c) deformation mesh to visualize the spatial transform output by registration algorithms, (d) the MSI score map was aligned to the HF data cube by the above transform, (e) ion maps of all *m*/*z* were registered, upsampled, and overlapped on top of the H&E image.

characteristics as discussed in [37]: the MSI of the mouse kidney shows narrow, spatially specific images in kidney regions but the H&E shows broad staining across the kidney and a combination of stain color and morphology is used to delineate regions. The HF data solved this problem by (i) encoding the stain color and morphology information of H&E into the HF data cube, (ii) extracting region-specific images from the HF data cube with NMF, and (iii) performing registration between the two manually-matched region-specific images (i.e. single image representations). So, the registration approach introduced in this paper has the potential to become a generic method for MSI and microscopy. But more work needs to be done to explore this idea further.

Our strategy is in principle applicable to other MSI ionization technologies such as Secondary Ion Mass Spectroscopy (SIMS) or MALDI (note that the software tool we provide is coded in Python, so the data format output by MSI instruments must be Python compatible, e.g. imzML), but follow-up studies are required to test its genericity. In particular, MALDI and SIMS can achieve lateral resolutions greater than 10μm, in which case we anticipate that a more powerful spatial registration approach compatible with the single cell scale will be required to establish accurate correspondence between MSI pixel and H&E tile. Combining our method with high spatial resolution MSI data may further improve the granularity and accuracy of the spatial segmentation results. For example, the "unconfident pixels" at the borders between ROIs (Figure.3(d) and Figure.4(b-4)) may be subdivided and "deconvolved", resulting in their assignment to existing clusters or the creation of new clusters.

Finally, in our multimodal fusion strategy, other imaging modalities can be used instead of optical microscopy. As an alternative method to obtain a label-free distribution of molecular species within a sample, vibrational spectroscopic imaging techniques, such as Fourier Transform Infrared (FTIR) and Raman imaging, capture information about chemical bonds, making them complementary to MSI and great candidates for multimodal corroboration of spatial segmentation. Specifically, we can replace the HF spectra extracted from each tile with corresponding FTIR or Raman spectra, and the rest of our strategy remains essentially the same.

Materials and Methods

Sample Preparation and Data Acquisition

The animal experiments were approved by the Animal Care and Use Committee of Shenzhen Institute of Advanced Technology, Chinese Academy of Sciences. A mouse kidney (7-month age female C57) was frozen-preserved at -80° before being cut into two consecutive slices by cryostat sectioning with a thickness of 20 μm and 10 μm respectively. MSI was done on the thicker slice with a 100 × 100 μm step size (i.e. pixel resolution) using a Desorption Spray Ionization (DESI) ion source (Prosolia, US) coupled to a Synapt G2-Si mass spectrometer (Waters, UK). HPLC grade methanol (Merck, Germany) was used in DESI experiments at a flow rate of 1.5 μl/min. In the positive ion mode, the capillary voltage of DESI was 4.2 kV with a sampling cone of 80 V. The nebulizing nitrogen gas pressure of DESI was 0.5 MPa and the ion transfer capillary was at a temperature of 150 °*C*. A mass range of 200 - 1,000 *m*/*z* was covered with a mass resolving power of 10,000. The thinner slice went though standard Hematoxylin and Eosin (H&E) staining protocol, followed by digital pathology scanning with 20× magnification (i.e. 0.5 × 0.5 μm pixel resolution). A murine model of human renal adenocarcinoma was established by orthotopically transplanting the ACHN cell line (Zhuhai BesTest Bio-Tech, China) into NOD/SCID male mice at 4 weeks of age and growing for 5 more weeks before sacrifice. Being capable of mimicking the organ-specific tumor environment in humans, orthotopic models have been routinely used in the MSI community for understanding the biomolecular mechanisms underlying cancers and for investigating therapeutic agents [38]. The same experimental procedures were followed for the tumor specimen, except that the pixel resolution of MSI was set to 50 × 50 μm^2 in adaptation to a small tumor and surrounding tissue area.

Data processing and analysis

MSI and H&E data preprocessing

Raw MSI data were preprocessed with R Cardinal 2 package[39] following standard data preprocessing protocols, including total-ion-count normalization, spectral smoothing, baseline reduction, peak picking, peak alignment, peak binning, and peak filtering. Pixels were classified as "foreground" or "background" (i.e. located inside or outside the tissue section) by applying thresholding on their sums of intensities of selected tissue-specific ions. We excluded ions that had stronger signals in the background than in the foreground because they appeared to be noisy and not biologically

informative. Raw H&E images were preprocessed using standard protocols including tissue detection and color normalization (HistomicsTK 1.2.3 package [40]). Then, for the kidney experiments, the whole slide image was uniformly split into an array of image tiles and each tile had a size of 200 × 200 pixels. Notably, the pixel size of MSI was 200 times larger than that of the H&E image (100 μm/0.5 μm), so each image tile had the same physical size as an MSI pixel. For the tumor specimen, the size of a H&E image tile was set to 100 × 100 pixels, in line with the smaller MSI pixel size (50 × 50 μm^2). Tiles containing a minimum tissue area no less than 90% were classified as "foreground" and others were "background". For the whole kidney specimen, all foreground MSI pixels and H&E image tiles were used for following analysis; for the tumor specimen, only the tumor area and its surroundings were used for following analysis to reduce computational cost. All data processing and analysis programs were implemented using Python 3.7 unless otherwise specified.

Encoding an H&E image tile into a HF spectrum

"Features" are a series of measurable properties to fully characterize an object in a data set. For MSI data, it is rather straightforward that *m*/*z* values are the features sufficient to convey molecular information of each spatial pixel. However, how to construct features to encode the histology appearance of a H&E image isn't as self-evident. In 31, Mormont et al. thoroughly investigated the performance of different DCNN architectures as off-the-shelf feature extractors for a group of histology and cytology object classification tasks. All DCNN were pre-trained using the ImageNet database[30]. An inner layer (conv5-block32-concat) of DenseNet 201 [41] empirically achieved the highest average performance in the tasks and thus stood out as our extractor of choice to encode the stain color and morphology of a 2D H&E image tile into a set of informative HF. To stay in keeping with ImageNet, our input tiles had to be resized to [224, 224, 3] through bilinear interpolation and each color channel had to be centered to zero. They were then forward propagated through a sequence of densely connected convolutional layers, where about 8.6 Giga FLOPs of calculation took place. Eventually, the output arrays after the concatenation operation at the conv5_block32 layer of DenseNet201 were 2-D globally average-pooled, Min-Max scaled, and reshaped as a "histomorphological feature (HF) spectrum" of 1,920 variables. The HF spectra of all the tiles were formatted into a hyperspectral data cube like MSI (Figure 1): each tile became a pixel and each (foreground) pixel had an HF spectrum. Image processing were implemented by OpenCV 4.2.0.32 and the pre-trained DCNN was downloaded from Keras 2.4.3.

Spatial registration between MSI and HF data

Registration was a prerequisite to fusing multimodal imaging data sets, where the MSI and HF data cubes were spatially aligned. We used a dimension reduction algorithm Nonnegative Matrix Factorization (NMF) to generate single image representations for the data cubes (as shown in Figure.5): (1) NMF reduced the data cubes into two sets of score maps, (2) we manually selected two score maps whose spatial expression best matched each other (see Figure S3) and used them as the inputs of following intensity-based registration algorithms. DESI-MSI preferred a section thickness of >20 μm to produce intense ion signals, which was above the recommended thickness for H&E staining microscopy (<10 μm). So, we had to use serial sections, one for MSI and one for H&E. This meant that there might be nonlinear deformation between the MSI and HF score maps due to inter-section anatomical variations or inconsistent sample preparation artifacts. Hence, we combined a linear registration algorithm (affine transform) with a nonlinear one (BSpline-based Free Form Deformation) to both align the score maps globally and correct local deformations. Mutual Information was used as the image similarity metric to guide the gradient descent-based optimization of linear and non-linear transforms. Finally, we applied the optimized transforms to all ion maps (*m*/*z* channels). To evaluate registration quality, two representatives of registered ion maps (*m*/*z* 834.4 and 683.3) were upsampled 200 times (spline interpolation of 3 order) and overlaid on top of H&E image in Figure.5(e). Figure.S12 shows a visual assessment of the registration quality: good overlap between the HF-derived and MSI-derived tissue masks, and between the distinct anatomical features observed in both HF and MSI images, indicate good registration results. More about the registration process in *SI*. Scikit-learn 0.24.2, Scikit-image 0.18.1, and SimpleITK 2.1.0 libraries were used to implement the above algorithms.

Segmentation and multimodal fusion-based evaluation

Spectral clustering is a graph-based clustering algorithm reported to outperform traditional algorithms such as k-means in MSI data-based tissue segmentation[42]. So, we used spectral clustering to group foreground pixels based on their HF and mass spectral similarities respectively (more details in *SI*) and obtained two sets of clustering/segmentation results (i.e. two sets of labels for each pixel). Then, CKS[43] was employed to compare the two sets of results: perfect agreement gave a CKS of 1.0 and random labeling gave a CKS close to 0.0. The mathematical definition of CKS is given in the *SI*. By taking into account the possibility of the agreement occurring by chance, CKS is not biased towards small #Clusters and hence a better measure than simple percentage agreement calculation. Note that the labeling of the clusters/regions between the two modalities was aligned by finding the largest CKS of all possible permutations. So, for a given #Clusters, CKS evaluated the biological validity of MSI-segmentation by how much it was reproduced by histology-segmentation. Figure S1 provides a step-by-step illustration of the entire methodology.

Availability of source code and requirements

Source codes from this work are freely accessible at https://github.com/guoang4github/ROIforMSI/ (Licence: GPL-3). Our software tool is also registered in the bio.tools (roiformsi) and SciCrunch (RRID:SCR_023275) databases. Computational workflow is registered in workflowhub.eu (https://doi.org/10.48546/WORKFLOWHUB.WORKFLOW.437.1)

Availability of supporting data and materials

The MSI data have been deposited to the ProteomeXchange Consortium (http://proteomecentral.proteomexchange.org) via the iProX partner repository [44] with the dataset identifier PXD038876.

Declarations

List of abbreviations

MSI: Mass spectrometry imaging; ROI: Region-of-interest; H&E: Haemotoxylin and Eosin; DCNN: Deep convolutional neural network; HF: histomorphological features; PC: Pearson correlation; CKS: Cohen's kappa score; DBI: Davies–Bouldin index; Nonnegative Matrix Factorization (NMF); MALDI: Matrix-Assisted Laser Desorption Ionization; DESI: Desorption electrospray ionization; UMAP: Uniform manifold approximation and projection

Ethical Approval

The animal experiments were approved by the Animal Care and Use Committee of Shenzhen Institute of Advanced Technology, Chinese Academy of Sciences.

Consent for publication

Not applicable

Competing Interests

The author(s) declare that they have no competing interests

Funding

This study was financially supported by the National Natural Science Foundation of China (82127801 and 22076197), the Scientific Instrument Developing Project of the Chinese Academy of Sciences (YJKYYQ20200034), Shenzhen Engineering Laboratory of Single-molecule Detection and Instrument Development (XMHT20190204002), Shenzhen Science and Technology Innovation Commission (JCYJ20200109115405930), Basic and Applied Basic Research Foundation of Guangdong Province (JCYJ20210324115811031, JCYJ20200109115405930, and 2021A1515110096), Shenzhen Institute of Advanced Technology (SIAT) Innovation Program for Excellent Young Researchers (2019), and Guangzhou Life Sciences Facility Center of the Chinese Academy of Sciences Open Research Program (GZQY202004).

Author's Contributions

A.G., F.L., and Q.L. designed research; A.G.and Z.C. performed research; A.G. analyzed data; and A.G. and Q.L. wrote the paper.

Acknowledgements

We thank Dr.Yueguang Lv for his comments that significantly improved this manuscript.

References

1. McDonnell LA, Heeren RMA. Imaging mass spectrometry. Mass Spectrometry Reviews 2007;.
2. Norris JL, Caprioli RM. Imaging mass spectrometry: A new tool for pathology in a molecular age. Proteomics Clinical Applications 2013;7(11-12):733–738.
3. Chaurand P, Schwartz SA, Caprioli RM. Imaging mass spectrometry: a new tool to investigate the spatial organization of peptides and proteins in mammalian tissue sections. Current Opinion in Chemical Biology 2002;6(5):676–681.
4. Seeley EH, Caprioli RM. Molecular Imaging Of Proteins In Tissues By Mass Spectrometry. Proceedings of the National Academy of Sciences of the United States of America 2008;105(47):18126–18131.
5. Cole LM, Clench MR. Mass spectrometry imaging tools in oncology. Biomarkers in medicine 2015;9(9):863–868.
6. Schnackenberg LK, Thorn DA, Barnette D, Jones EE. MALDI imaging mass spectrometry: An emerging tool in neurology. Metabolic Brain Disease 2021;p. 1–17.
7. Watrous JD, Dorrestein PC. Imaging mass spectrometry in microbiology. Nature Reviews Microbiology 2011;9(9):683–694.
8. Nilsson A, Goodwin RJ, Shariatgorji M, Vallianatou T, Webborn PJ, Andrén PE. Mass spectrometry imaging in drug development. Analytical chemistry 2015;87(3):1437–1455.
9. Thomas A, Patterson NH, Marcinkiewicz MM, Lazaris A, Metrakos P, Chaurand P. Histology-driven data mining of lipid signatures from multiple imaging mass spectrometry analyses: application to human colorectal cancer liver metastasis biopsies. Analytical chemistry 2013;85(5):2860–2866.
10. Verbeeck N, Caprioli RM, de Plas RV. Unsupervised machine learning for exploratory data analysis in imaging mass spectrometry. Mass Spectrometry Reviews 2020;.
11. Mccombie G, Staab D, Stoeckli M, Knochenmuss R. Spatial and Spectral Correlations in MALDI Mass Spectrometry Images by Clustering and Multivariate Analysis. Analytical Chemistry 2005;77(19):6118–6124.
12. Deininger S, Ebert M, Futterer A, Gerhard M, Rocken C. MALDI imaging combined with hierarchical clustering as a new tool for the interpretation of complex human cancers. Journal of Proteome Research 2008;7(12):5230–5236.
13. Alexandrov T, Becker M, Deininger S, Ernst G, Wehder L, Grasmair M, et al. Spatial segmentation of imaging mass spectrometry data with edge-preserving image denoising and clustering. Journal of Proteome Research 2010;9(12):6535–6546.
14. Kobarg JH. Efficient spatial segmentation of large imaging mass spectrometry datasets with spatially aware clustering. Bioinformatics 2011;27(13):p.230–238.
15. Inglese P, Mckenzie JS, Mroz A, Kinross J, Veselkov K, Holmes E, et al. Deep learning and 3D-DESI imaging reveal the hidden metabolic heterogeneity of cancer. Chemical Science 2017;8(5):3500–3511.
16. Delcourt V, Franck J, Leblanc E, Narducci F, Robin YM, Gimeno JP, et al. Combined mass spectrometry imaging and top-down microproteomics reveals evidence of a hidden proteome in ovarian cancer. EBioMedicine 2017;21:55–64.
17. Song X, He J, Pang X, Zhang J, Sun C, Huang L, et al. Virtual calibration quantitative mass spectrometry imaging for accurately mapping analytes across heterogenous biotissue. Analytical chemistry 2019;91(4):2838–2846.
18. Jones MA, Cho SH, Patterson NH, Van de Plas R, Spraggins JM, Boothby MR, et al. Discovering new lipidomic features using cell type specific fluorophore expression to provide spatial and biological specificity in a multimodal workflow with MALDI Imaging Mass Spectrometry. Analytical chemistry 2020;92(10):7079–7086.
19. Taylor AJ, Dexter A, Bunch J. Exploring ion suppression in mass spectrometry imaging of a heterogeneous tissue. Analytical chemistry 2018;90(9):5637–5645.
20. Bemis KD, Harry A, Eberlin LS, Ferreira CR, Vitek O. Probabilistic segmentation of mass spectrometry images helps select important ions and characterize confidence in the resulting segments. Molecular Cellular Proteomics Mcp 2016;15(5):mcp.O115.053918.
21. Abdelmoula WM, Balluff B, Englert S, Dijkstra J, Reinders MJT, Walch A, et al. Data-driven identification of prognostic tumor subpopulations using spatially mapped t-SNE of mass spectrometry imaging data. Proceedings of the National Academy of Sciences of the United States of America 2016;113(43):12244–12249.
22. Liu Y, Li Z, Xiong H, Gao X, Wu J. Understanding of internal clustering validation measures. In: 2010 IEEE International Conference on Data Mining IEEE; 2010. p. 911–916.
23. Moulavi D, Jaskowiak PA, Campello RJ, Zimek A, Sander J. Density-based clustering validation. In: Proceedings of the 2014 SIAM international conference on data mining SIAM; 2014. p. 839–847.
24. Neumann EK, Djambazova KV, Caprioli RM, Spraggins JM. Multimodal imaging mass spectrometry: next generation molecular mapping in biology and medicine. Journal of the American Society for Mass Spectrometry 2020;31(12):2401–2415.
25. Van de Plas R, Yang J, Spraggins J, Caprioli RM. Image fusion of mass spectrometry and microscopy: a multimodality paradigm for molecular tissue mapping. Nature methods 2015;12(4):366–372.
26. Rappez L, Stadler M, Triana S, Gathungu RM, Ovchinnikova K, Phapale P, et al. SpaceM reveals metabolic states of single cells.

Nature methods 2021;18(7):799–805.

27. LeCun Y, Bengio Y, Hinton G. Deep learning. nature 2015;521(7553):436–444.
28. Coudray N, Ocampo PS, Sakellaropoulos T, Narula N, Snuderl M, Fenyö D, et al. Classification and mutation prediction from non–small cell lung cancer histopathology images using deep learning. Nature medicine 2018;24(10):1559–1567.
29. Weiss K, Khoshgoftaar TM, Wang DD. A survey of transfer learning. Journal of Big Data 2016;3(1):9.
30. ImageNet Large Scale Visual Recognition Challenge. International Journal of Computer Vision 2015;.
31. Mormont R, Geurts P, Maree R. Comparison of Deep Transfer Learning Strategies for Digital Pathology. In: 2018 IEEE/CVF Conference on Computer Vision and Pattern Recognition Workshops (CVPRW); 2018. .
32. McInnes L, Healy J, Melville J. Umap: Uniform manifold approximation and projection for dimension reduction. arXiv preprint arXiv:180203426 2018;.
33. Smets T, Verbeeck N, Claesen M, Asperger A, Griffioen G, Tousseyn T, et al. Evaluation of distance metrics and spatial autocorrelation in uniform manifold approximation and projection applied to mass spectrometry imaging data. Analytical chemistry 2019;91(9):5706–5714.
34. Zhu Y, Zang Q, Luo Z, He J, Zhang R, Abliz Z. An Organ-Specific Metabolite Annotation Approach for Ambient Mass Spectrometry Imaging Reveals Spatial Metabolic Alterations of a Whole Mouse Body. Analytical Chemistry 2022;94(20):7286–7294.
35. Robert, J, Motzer, Neil, H, Bander, et al. Renal-Cell Carcinoma. New England Journal of Medicine 1996;.
36. Vakkila J, Lotze MT. Inflammation and necrosis promote tumour growth. Nature Reviews Immunology 2004;4(8):641–648.
37. Tuck M, Blanc L, Touti R, Patterson NH, Van Nuffel S, Villette S, et al. Multimodal imaging based on vibrational spectroscopies and mass spectrometry imaging applied to biological tissue: a multiscale and multiomics review. Analytical chemistry 2020;93(1):445–477.
38. Goodwin R, Bunch J, McGinnity D. Mass spectrometry imaging in oncology drug discovery. Advances in cancer research 2017;134:133–171.
39. Kyle, D, Bemis, April, Harry, Livia, et al. Cardinal: an R package for statistical analysis of mass spectrometry-based imaging experiments: Fig. 1. Bioinformatics 2015;.
40. Cooper L, HistomicsTK; 2016. https://github.com/DigitalSlideArchive/HistomicsTK.
41. Huang G, Liu Z, Van Der Maaten L, Weinberger KQ. Densely connected convolutional networks. In: Proceedings of the IEEE conference on computer vision and pattern recognition; 2017. p. 4700–4708.
42. Dexter A, Race AM, Steven RT, Barnes JR, Hulme H, Goodwin RJ, et al. Two-phase and graph-based clustering methods for accurate and efficient segmentation of large mass spectrometry images. Analytical chemistry 2017;89(21):11293–11300.
43. McHugh, Mary, L. Interrater reliability: the kappa statistic. Biochemia Medica 2012;.
44. Ma J, Chen T, Wu S, Yang C, Bai M, Shu K, et al. iProX: an integrated proteome resource. Nucleic acids research 2019;47(D1):D1211–D1217.

Figure 1

Click here to access/download;Figure;(1) overview_revised.jpg

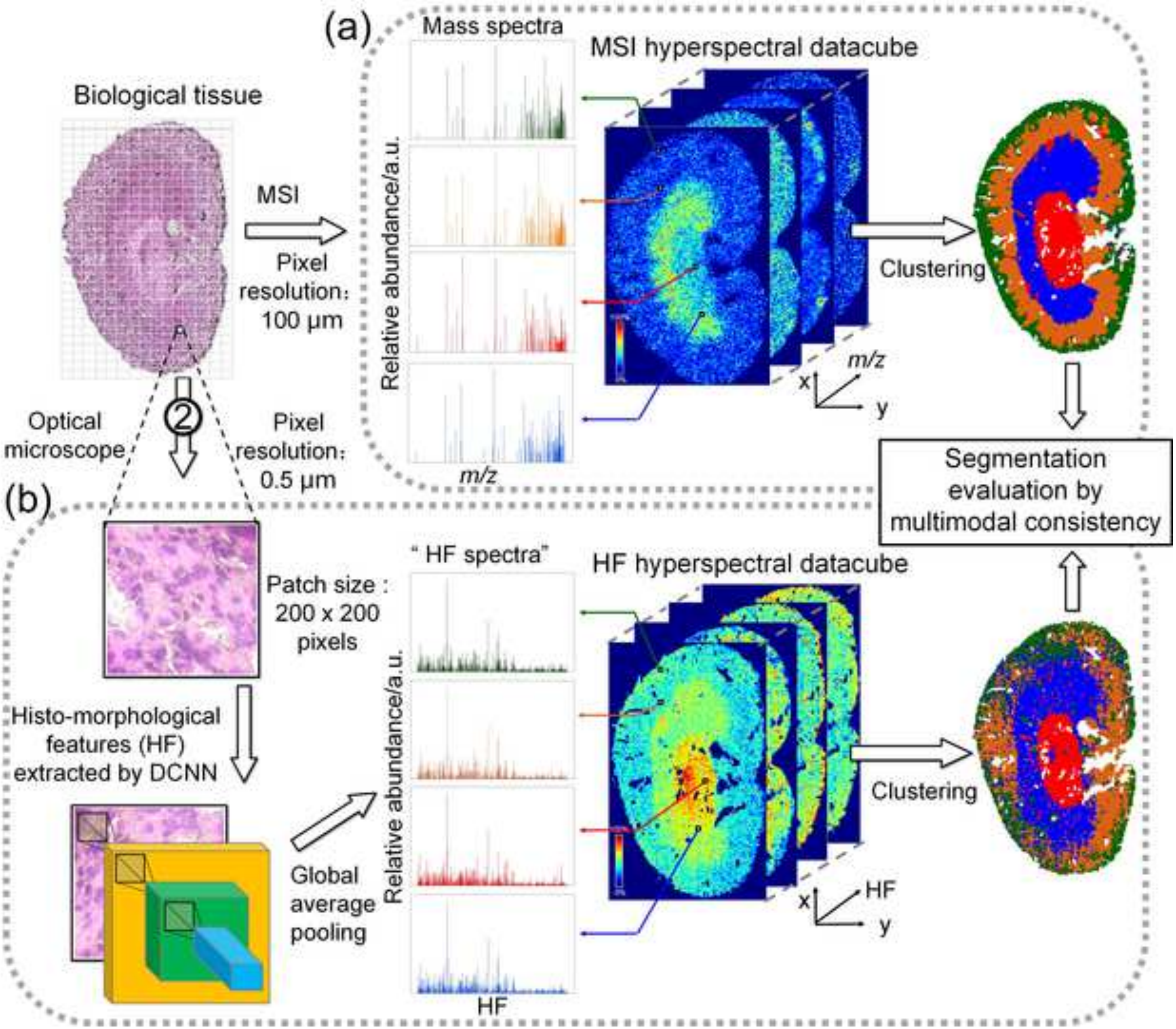

Figure 4

Click here to access/download;Figure;(4) kidney_segments_revised.jpg

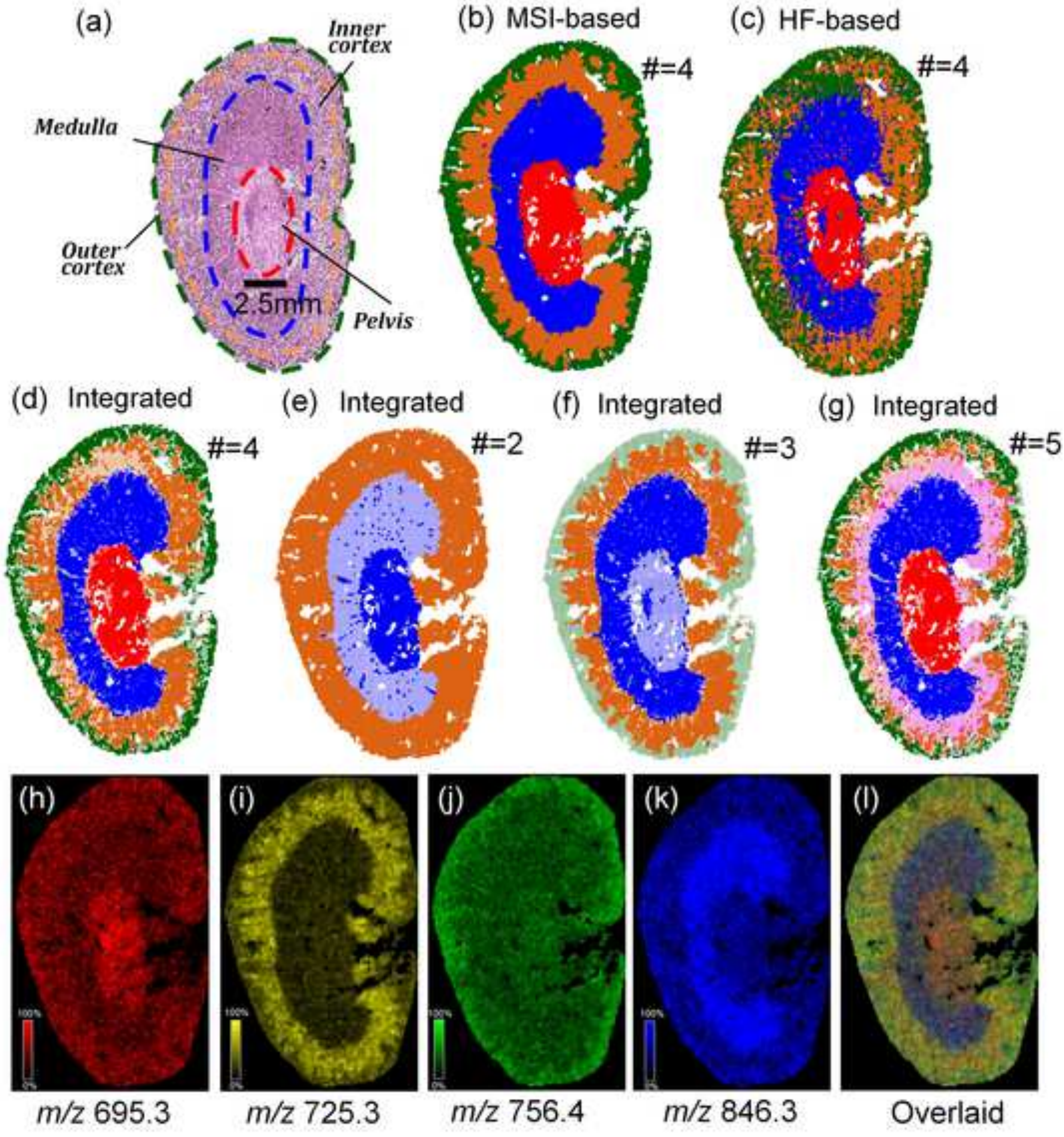

Figure 3

Click here to access/download;Figure;(3) No_of_clusters_plot_revised.jpg

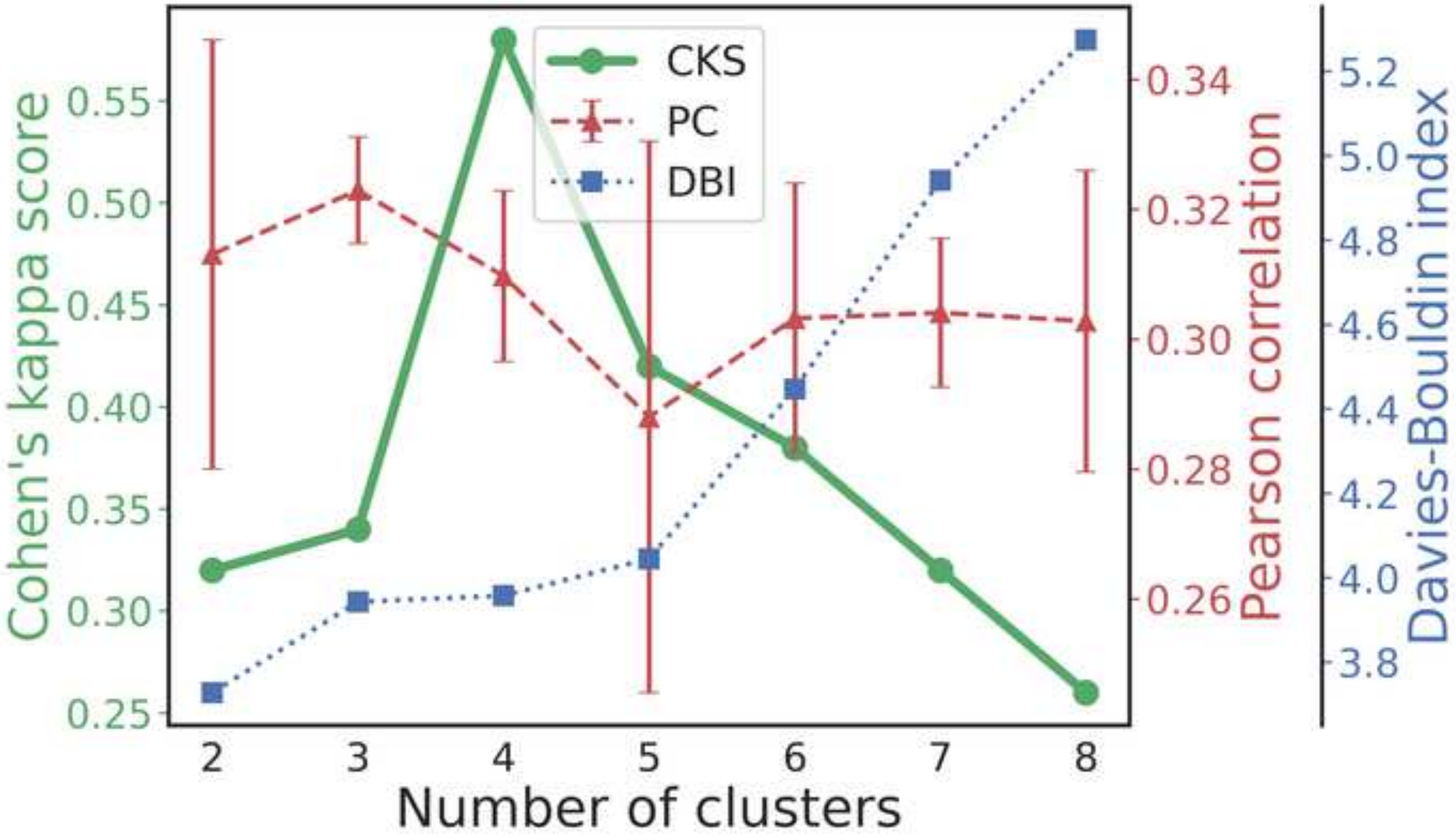

Figure 2

Click here to access/download;Figure;(2) kidney_registration_revised.jpg

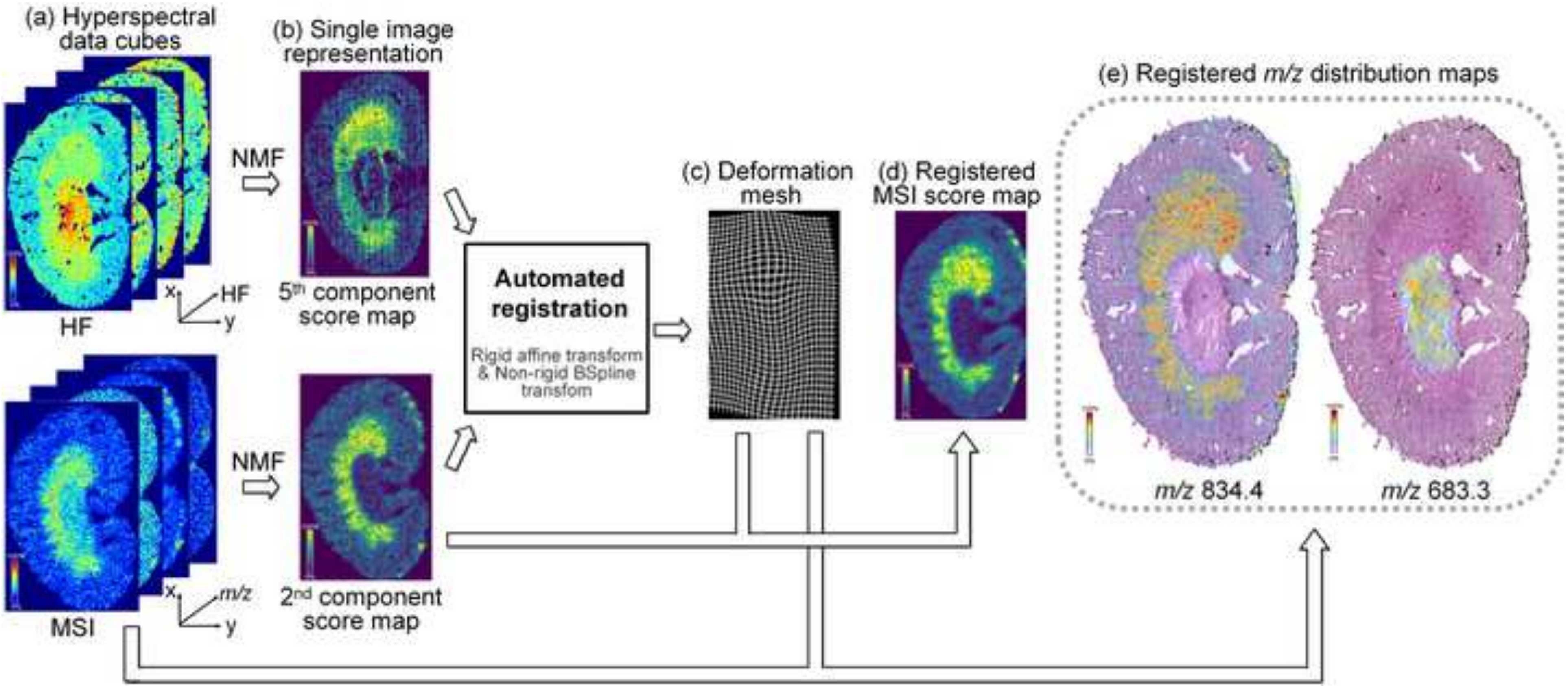

Figure

Click here to access/download;Figure;(5) tumor_segments.jpg

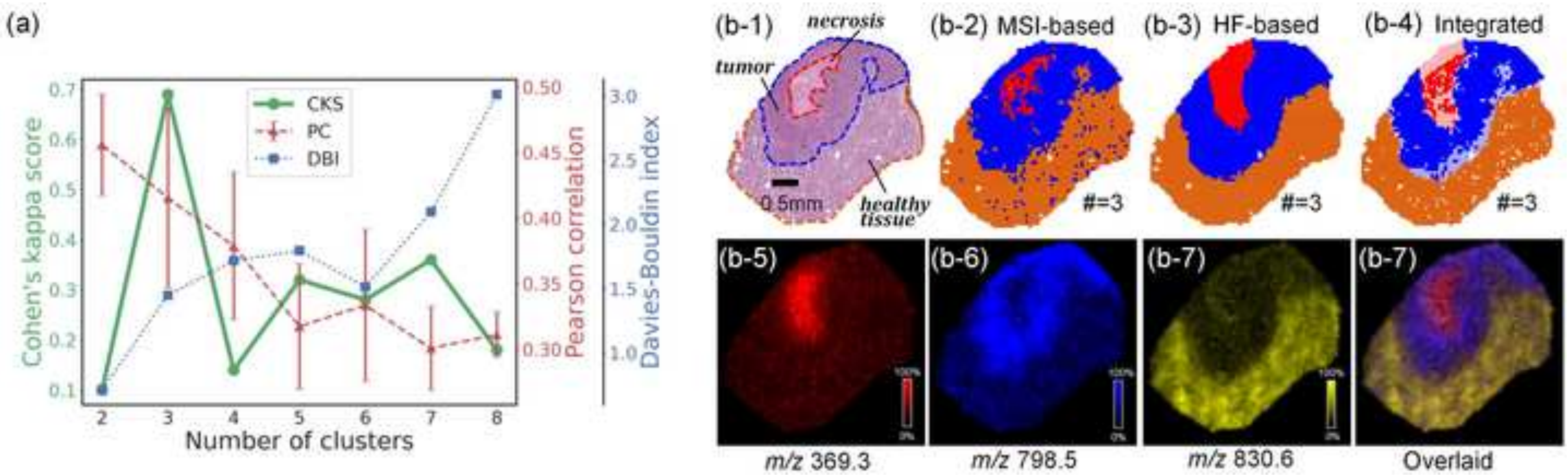

supplementary texts and figures

Click here to access/download

Supplementary Material

GigaScience_Supporting_Information_revised_230209.pdf

Cover letter
Click here to access/download;Personal Cover;cover letter.docx

Dear Editor:

I am writing to submit our manuscript entitled, "**Delineating Regions-of-interest for Mass Spectrometry Imaging by Multimodally Corroborated Spatial Segmentation**" for consideration as a research article for *GigaScience*.

Mass Spectrometry Imaging (MSI) is a tag-free molecular imaging technique for biological samples. It is capable of simultaneous localizing tens to hundreds of biomolecules in a single experiment by acquiring a full mass spectrum in each pixel of a virtual grid. In MSI data analysis, spatial segmentation, obtained by clustering MSI pixels according to their mass spectral similarities, is a popular approach to delineate Regions-of-Interest (ROIs) that correspond to tissue areas of different anatomical or pathological labels. However, there is currently no good strategy to select the number of clusters (#Clusters), which is the most important parameter for clustering algorithms and determines the granularity of the segmentation. Wrong #Clusters may lead to ROIs that are not biologically real. Here, we propose a novel multimodal fusion strategy that uses information from segmentation of a histopathology image to inform the task of segmentation of an MSI image. (i) a Deep Learning-based method is proposed to produce histology-segmentation from H&E images; (ii) the most probable #Clusters is determined by using the consistency between histology- and MSI- segmentations as a quantitative measure of biological validity. Our strategy has the merits of being objective and rigorous and ensures that its produced ROIs are of biological relevance supported by both MSI and histology.

Given that MSI is being widely used in various areas of life sciences and that segmentation-based ROI delineation is an important step of MSI data analysis, we believe that the method presented in our paper will appeal to a broad range of readers who are current or potential MSI users. In addition, more general readers, who are interested in interdisciplinary research that applies Artificial Intelligence in life sciences, may also find our work valuable.

We have published a subset of our findings in a preprint repository BioArxiv in 2020 (doi: https://doi.org/10.1101/2020.07.17.208025). Part of the MSI data has been used in another article ("Multimodal Coregistration and Fusion between Spatial Metabolomics and Biomedical Imaging", in submission) but for entirely different research purposes.

Each named author has approved the contents of this paper, agreed to the *GigaScience*'s submission policies, and substantially contributed to conducting the underlying research and drafting this manuscript. Additionally, to the best of our knowledge, the named authors have no conflict of interest, financial or otherwise.

Sincerely,

Luo Qian
Ph.D. Professor
Institute of Biomedicin and Biotechnology,
Shenzhen Institute of Advanced Technology, Chinese Academy of Science.
E-mail:qian.luo@siat.ac.cn
