# Supplementary material for: Delineating regions of interest for mass spectrometry imaging by multimodally corroborated spatial segmentation
Source: Gigascience. 2023 Apr 11;12:giad021. doi: 10.1093/gigascience/giad021 (PMC10087011; doi:10.1093/gigascience/giad021)
Supplement: giad021_GIGA-D-22-00334_Revision_2 [file giad021_giga-d-22-00334_revision_2.pdf]

GigaScience

Delineating Regions-of-interest for Mass Spectrometry Imaging by Multimodally Corroborated Spatial Segmentation

--Manuscript Draft--

**Manuscript Number:**	GIGA-D-22-00334R2
**Full Title:**	Delineating Regions-of-interest for Mass Spectrometry Imaging by Multimodally Corroborated Spatial Segmentation
**Article Type:**	Research

Funding Information:

Funder	Recipient
the National Natural Science Foundation of China (82127801)	Prof qian luo
the National Natural Science Foundation of China (22076197)	Dr Fang Li
Shenzhen Science and Technology Innovation Commission (JCYJ20200109115405930)	Prof qian luo
Shenzhen Science and Technology Innovation Commission (JCYJ20210324115811031)	Not applicable
Guangdong Basic and Applied Basic Research Foundation (2020B1515120080)	Prof qian luo
Guangdong Basic and Applied Basic Research Foundation (2021A1515110096)	Dr Ang Guo
Shenzhen Institutes of Advanced Technology Innovation Program for Excellent Young Researchers	Dr Ang Guo
Guangzhou Life Sciences Facility Center of the Chinese Academy of Sciences Open Research Program (GZQY202004)	Dr Ang Guo
National Key R&D Program of China (2022YFF0705003)	Prof qian luo

Abstract:

Mass spectrometry imaging, which localizes molecules in a tag-free, spatially-resolved manner, is a powerful tool for the understanding of underlying biochemical mechanisms of biological phenomena. When analyzing MSI data, it is essential to delineate Regions-of-Interest (ROIs) that correspond to tissue areas of different anatomical or pathological labels. Spatial segmentation, obtained by clustering MSI pixels according to their mass spectral similarities, is a popular approach to automate ROI definition. However, how to select the number of clusters (#Clusters), which determines the granularity of segmentation, remains to be resolved, and an inappropriate #Clusters may lead to ROIs not biologically real. Here we report a multimodal fusion strategy to enable an objective and trustworthy selection of #Clusters by utilizing additional information from corresponding histology images. A Deep Learning-based algorithm is proposed to extract "histomorphological feature spectra" across an entire H\&E image. Clustering is then similarly performed to produce Histology-segmentation. Since ROIs originating from instrumental noise or artifacts wouldn't be reproduced cross-modally, the consistency between histology- and MSI-segmentation becomes an effective measure of the biological validity of the results. So, #Clusters that maximizes the consistency is deemed as most probable. We validated our strategy on mouse kidney and renal tumor specimens by producing multimodally corroborated ROIs that agreed excellently with ground truths. Downstream analysis based on the said ROIs revealed lipid molecules highly specific to tissue anatomy or pathology. Our work will greatly facilitate MSI-mediated spatial lipidomics, metabolomics, and proteomics research by providing intelligent software to automatically and reliably generate ROIs.

Corresponding Author:

qian luo, Ph.D.
Shenzhen Institute of Advanced Technology, Chinese Academy of Sciences

Powered by Editorial Manager® and ProduXion Manager® from Aries Systems Corporation

	Shenzhen, CHINA
**Corresponding Author Secondary Information:**	
**Corresponding Author's Institution:**	Shenzhen Institute of Advanced Technology, Chinese Academy of Sciences
**Corresponding Author's Secondary Institution:**	
**First Author:**	Ang Guo
**First Author Secondary Information:**	
**Order of Authors:**	Ang Guo
	Zhiyu Chen
	Fang Li
	qian luo, Ph.D.
**Order of Authors Secondary Information:**	
**Response to Reviewers:**	1. DOIs should be cited in the references and only Reference # cited in the main text.<br><br>response: DOIs have been moved to the references.<br><br>2. Please add the GigaDB DOI citation - I have included exact text on what to add under "Availability of supporting data" and also how to cite the DOI in the references. Please add the Reference number to the Data availability section.<br><br>response: GigaDB DOI citation has been added.
**Additional Information:**	
**Question**	**Response**
Are you submitting this manuscript to a special series or article collection?	No
**Experimental design and statistics**<br><br>Full details of the experimental design and statistical methods used should be given in the Methods section, as detailed in our Minimum Standards Reporting Checklist. Information essential to interpreting the data presented should be made available in the figure legends.<br><br>Have you included all the information requested in your manuscript?	Yes
**Resources**<br><br>A description of all resources used, including antibodies, cell lines, animals and software tools, with enough information to allow them to be uniquely	Yes

identified, should be included in the Methods section. Authors are strongly encouraged to cite Research Resource Identifiers (RRIDs) for antibodies, model organisms and tools, where possible.<br><br>Have you included the information requested as detailed in our Minimum Standards Reporting Checklist?	
**Availability of data and materials**<br><br>All datasets and code on which the conclusions of the paper rely must be either included in your submission or deposited in publicly available repositories (where available and ethically appropriate), referencing such data using a unique identifier in the references and in the "Availability of Data and Materials" section of your manuscript.<br><br>Have you have met the above requirement as detailed in our Minimum Standards Reporting Checklist?	Yes

Click here to access/download;LaTeX - Main Document (TeX file);main.tex

```
This is pdfTeX, Version 3.141592653-2.6-1.40.24 (TeX Live 2022)
(preloaded format=pdflatex 2023.2.6)  12 MAR 2023 23:11
entering extended mode
 restricted \write18 enabled.
 %&-line parsing enabled.
**main.tex
(./main.tex
LaTeX2e <2022-11-01>
L3 programming layer <2023-02-02>

! LaTeX Error: File `oup-contemporary.cls' not found.

Type X to quit or <RETURN> to proceed,
or enter new name. (Default extension: cls)

Enter file name:
! Emergency stop.
<read *>

l.11 ^^M

*** (cannot \read from terminal in nonstop modes)


Here is how much of TeX's memory you used:
 23 strings out of 476067
 503 string characters out of 5794940
 1849326 words of memory out of 5000000
 20528 multiletter control sequences out of 15000+600000
 512287 words of font info for 32 fonts, out of 8000000 for 9000
 1141 hyphenation exceptions out of 8191
 19i,0n,29p,95b,17s stack positions out of
10000i,1000n,20000p,200000b,200000s
!  ==> Fatal error occurred, no output PDF file produced!
```

Manuscript

Click here to access/download;Manuscript;GigaScience_Journal_Manuscrip

GigaScience, 2017, 1–9

doi: xx.xxxx/xxxx
Manuscript in Preparation
Paper

PAPER

Delineating Regions-of-interest for Mass Spectrometry Imaging by Multimodally Corroborated Spatial Segmentation

Ang Guo[1], Zhiyu Chen[1,2], Fang Li[1] and Qian Luo[1,2,*]

[1]Institute of Biomedicine and Biotechnology, Shenzhen Institute of Advanced Technology, Chinese Academy of Sciences, Shenzhen, China. and [2]University of Chinese Academy of Sciences, Beijing, China.

[*]qian.luo@siat.ac.cn

Abstract

Mass spectrometry imaging, which localizes molecules in a tag-free, spatially-resolved manner, is a powerful tool for the understanding of underlying biochemical mechanisms of biological phenomena. When analyzing MSI data, it is essential to delineate Regions-of-Interest (ROIs) that correspond to tissue areas of different anatomical or pathological labels. Spatial segmentation, obtained by clustering MSI pixels according to their mass spectral similarities, is a popular approach to automate ROI definition. However, how to select the number of clusters (#Clusters), which determines the granularity of segmentation, remains to be resolved, and an inappropriate #Clusters may lead to ROIs not biologically real. Here we report a multimodal fusion strategy to enable an objective and trustworthy selection of #Clusters by utilizing additional information from corresponding histology images. A Deep Learning-based algorithm is proposed to extract "histomorphological feature spectra" across an entire H&E image. Clustering is then similarly performed to produce Histology-segmentation. Since ROIs originating from instrumental noise or artifacts wouldn't be reproduced cross-modally, the consistency between histology- and MSI-segmentation becomes an effective measure of the biological validity of the results. So, #Clusters that maximizes the consistency is deemed as most probable. We validated our strategy on mouse kidney and renal tumor specimens by producing multimodally corroborated ROIs that agreed excellently with ground truths. Downstream analysis based on the said ROIs revealed lipid molecules highly specific to tissue anatomy or pathology. Our work will greatly facilitate MSI-mediated spatial lipidomics, metabolomics, and proteomics research by providing intelligent software to automatically and reliably generate ROIs.

Key words: Mass spectrometry imaging; Spatial segmentation; Multimodal data fusion

Introduction

The alterations and interactions of biochemical pathways are often spatially-heterogeneous in a complex biological system, thus localizing molecules in a spatially-resolved manner is crucial for deciphering underlying biochemical mechanisms of biological phenomena. Mass Spectrometry Imaging (MSI) is a tag-free, high-throughput molecular mapping technique, which simultaneously acquires the spatial distributions of tens to hundreds of molecules by collecting a full mass spectrum in each pixel of a virtual grid [1, 2]. MSI can cover a wide variety of biomolecular species (including proteins, peptides, lipids, and metabolites) over biological samples with great sensitivity and chemical specificity [1, 2]. Since its emergence in the early 2000s [3, 4], MSI has enabled new biochemical discoveries in a wide range of life sciences including oncology [5], neurology [6], microbiology [7], and drug development [8]. During the post-acquisition analysis of MSI data, an intact tissue section is often virtually segmented into a number of Regions-of-Interest (ROIs) that correspond to different anatomical or pathological labels [1, 9]. An accurate definition of ROIs allows the extraction of tissue-type specific molecular abundances, which are essential for statistically discovering molecular alterations between different ROIs of the same specimen (for example, tumor versus normal regions

Compiled on: March 13, 2023.
Draft manuscript prepared by the author.

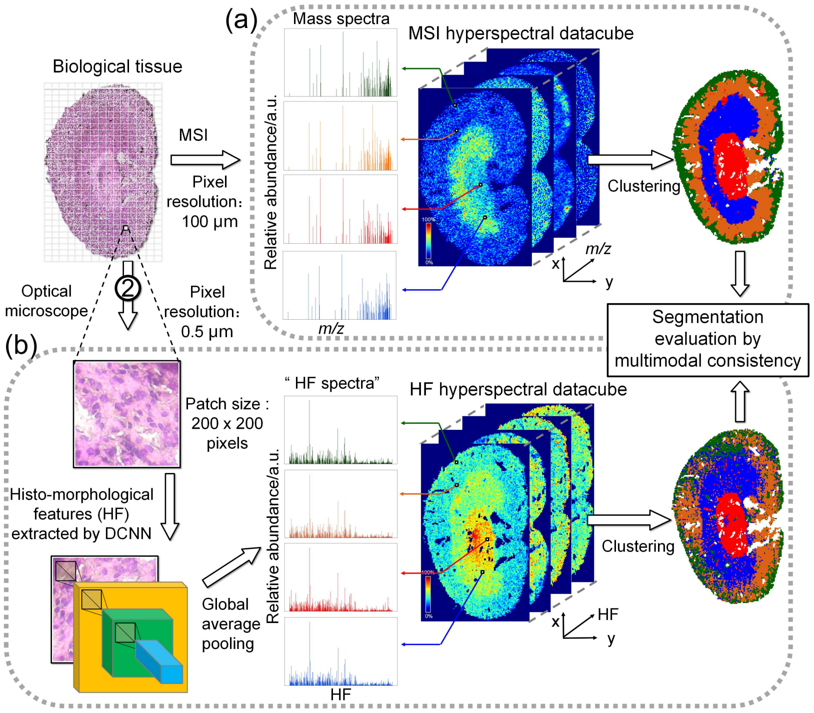

Figure 1. The overview of tissue segmentation based on (a) MSI data and (b) H&E stained histology image. In (a), mass spectra acquired by MSI can be formatted as a hyperspectral data cube. In (b), a high-resolution histology image was divided into an array of small tiles of size 200 $\times$ 200 pixels. A set of quantitative histomorphological features (HF) was then computed from each tile by a DCNN-based feature extractor. So another hyperspectral data cube similar to the MSI data was generated, with the only difference that the depth corresponded to HF rather than *m/z*. Clustering analysis in the spectral domain resulted in segmentation in the spatial domain. Segmentation/ROI validation was achieved by comparing the MSI- and histology-based results.

of a tissue section), or between different specimens at the same ROI (for example, differentially expressed molecules at a specific anatomical structure between diseased versus healthy samples).

There are two commonly-used methods for defining ROIs. The first is manual annotation guided by the staining microscopy of a tissue specimen section (or its consecutive slice) [9]. Manual annotation requires solid expert knowledge of histology and is prone to human bias. It can also be rather time-consuming when the sample size and/or the quantity of ROIs are large. Spatial segmentation [10] is the alternative approach for ROI definition, which is data-driven in nature and substantially automated. For a set of mass spectral profiles collected at different positions (i.e. pixels), a clustering algorithm separates them into several groups (i.e. clusters) in such a way that spectra in the same group are "similar" to each other than to those in other groups. Pixels of identical tissue types are expected to be similar in their chemical content and thus mass spectral profiles. So, by labeling each pixel with the color assigned to its cluster, we can obtain spatial segmentation along the image domain as shown in Figure 1(a). Following the pioneering work of Mccombie et al., the use of clustering analysis to discover geographically separated and molecularly distinct tissue regions from MSI data has been increasingly adopted by MSI community [12, 13, 14, 15, 16, 17, 18]. However, it remains a challenge to properly configure the parameters of clustering algorithms, which have a huge impact on final segmentation results. For instance, the number of clusters (#Clusters) determines the number of regions (i.e. the granularity of spatial segmentation) for algorithms such as K-Means and spectral clustering, but it is arbitrarily predefined by users. As #Clusters gets larger, the clustering algorithm would start to segment the specimen on the basis of instrumental noise or acquisition artifacts, producing ROIs that are not biologically real. Such difficulty in setting parameters is largely owing to the lack of a method for rigorously evaluating segmentation results. So far in the MSI community, segmentation has been evaluated either by subjective judgments [12, 13, 16, 17, 19] or by certain internal clustering validation criteria [20, 21, 15]. The internal clustering validation [22] evaluates the "goodness" of clustering based only on the intrinsic structure of the MSI data, making it still susceptible to the influence of noise or artifacts. Also, different internal validation criteria can be built based on completely different rationales and may give rise to inconsistent outcomes [23], which further reduces their reliability. Consequently, there is an urgent need for an objective and biologically-reliable approach to evaluating MSI-segmentation results and determining the key parameters of clustering algorithms.

Integrating the data acquired by two or more biomedical imaging modalities is termed multimodal fusion, which holds the promise of enhancing the discrimination between modality-specific instrumental noise or artifacts and biologically relevant chemical signals [24, 25]. Specifically, segmentation based on a modality orthogonal to MSI can provide an external reference to evaluate the results generated by MSI. Of all the different imaging modalities, light microscopy is one of the most ideal candidates because of its good accessibility to the MSI community. For example, by integrating MALDI-MSI and microscopy, Rappez et al. [26] have developed a SpaceM method to characterize in situ single cell metabolomics. In addition, the last decade has seen a phenomenal success of Deep Learning [27] in the analysis of histology microscopic images [28]. Based on a well-established idea called "transfer learning" [29], deep convolutional neural networks (DCNN) that pre-trained using source datasets ImageNet [30], appear to be excellent off-the-shelf feature extractors to represent histology images with histomorphologically informative features, which has been used to achieve accurate classification of cell or tissue types [31]. Therefore, it becomes technically feasible to calculate histology-based segmentation according to histomorphological features (HF) extracted from the histology image.

In this article, we report a multimodal fusion strategy between histology microscopy and MSI to enable an objective and trustworthy selection of #Clusters. First, an in-house algorithm was

proposed to generate histology-segmentation from a H&E histology image. As shown in Figure 1, due to different lateral resolutions, each grid-scan pixel of MSI corresponds to a small tile of the whole slide H&E image of the same specimen. By propagating a tile through a DCNN-based feature extractor, we constructed an "HF spectrum" to represent the histomorphology of cellular neighborhoods located within an MSI pixel. Analogous to the MSI-segmentation, histology-segmentation was obtained by clustering the pixels according to their HF spectral similarities. Second, built upon an assumption that more reliable segmentation would be better replicated by orthogonal imaging modalities, we used the multimodal consistency between histology- and MSI- segmentation as a quantitative measure to evaluate validity. So, different #Clusters were compared accordingly and the one that produced maximum consistency was deemed most probable. Last, using the optimal #Clusters, pixels concordantly labeled by the two modalities were returned as ROIs of biological relevance supported by both molecular and histomorphological profiling. In the following sections, we used whole mouse kidney and tumor specimens as proof-of-concept examples to validate our strategy: ROIs produced by our strategy were in excellent agreement with ground truths. Based on the said ROIs, downstream data analysis revealed lipids that were highly specific to tissue anatomy or pathology: for the kidney specimen, DG(42:4), SM(d34:1), PC(34:3), and PC(40:0) appeared to be colocalized with the pelvis, inner cortex, outer cortex, and medulla, respectively; for the tumor specimen, MG(16:0), PC(34:1), and PC(38:5) were colocalized with the necrosis, viable tumor, and normal tissues. We expect to facilitate MSI data analysis by offering an intelligent tool for ROI delineation that is biologically reliable and intrinsically immune to subjective bias.

Results and discussion

Spatial segmentation based on MSI and HF data

The kidney specimen was segmented into different regions along the image domain by clustering the MSI or HF data cubes on the basis of the spectral domain. The results are shown in Figure S4 for different #Clusters. As #Clusters became larger, finer anatomical details were increasingly revealed by both modalities until highly fragmented and scattered regions started to appear at larger #Clusters (>=6), which probably originated from modality-specific and instrumental noise/artifacts. Figure S5(a) shows the 3D embedding of all the 1920D HF spectra extracted from the H&E image tiles (here the embedding was realized by UMAP-based [32] dimensionality reduction). To sanity check whether segmentation based on HF was indeed histomorphology-guided, we set #Clusters to 4 and retrieved 4 representative tiles that corresponded to the 4 data points located at the centers of each cluster (Figure S5(a)). Clearly, the 4 representative tiles had distinct histological appearances, which was in line with our expectations.

Determining #Clusters

#Clusters is the most important parameter for the spectral clustering algorithm, which directly determines the granularity of ROIs used in downstream statistical analysis and may have a major impact on final scientific findings. To select the most probable #Clusters for the kidney specimen, the MSI-segmentation results using different #Clusters had to be compared by certain clustering validation criteria. We first employed two internal validation measures proposed previously in literature [21, 15]. In 21, segmentation was evaluated by how closely it resembled the low-dimension overview of the high-dimensional molecular content of MSI data obtained by nonlinear dimension reduction techniques (here we used UMAP as recommended in [33]). The resemblance between the UMAP image and the segmentation maps was measured by applying a Canny edge detector to both and computing their edge correlation. As shown by the red dashed line in Figure 2, a higher Pearson correlation (PC) suggested higher resemblance and the maximum was at #Clusters=3. It appeared that the PC had relatively large error bars (standard deviation of PC values produced by five runs). This was because the specific choices of false color for each region appeared to considerably affect the Canny edges detected from the segmentation maps and in our experiment RGB color codes were randomly generated for each region in each run. In 15, the Davies–Bouldin index (DBI) was used to find the optimal segmentation for MSI data. DBI measures the ratio of within-cluster distances (the measure of intra-cluster compactness) to between-cluster distances (the measure of inter-cluster separation), so a smaller DBI indicates better-defined clusters and thus supposedly better segmentation. The DBI method suggested #Clusters=2 as the optimal (the blue dotted line in Figure 2). Both PC and DBI methods used internal information provided by the MSI data alone, so they could be vulnerable to instrumental noise or biologically irrelevant variations caused by experimental artifacts. It was also observed that different internal criteria might rank the goodness of the same clustering results in distinct ways. Such inconsistency was not surprising since those two criteria were designed based on very different rationales, but it might arouse concerns about the reproducibility and reliability of these internal criteria.

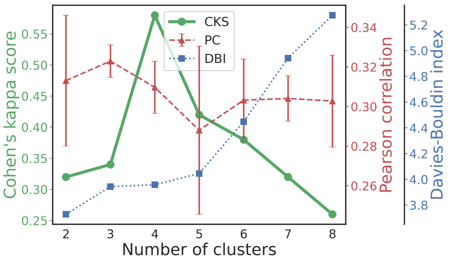

Figure 2. Determining the most probable #Clusters for the mouse kidney MSI data by (red) the PC-based method, (blue) the DBI-based method, and (green) our CKS-based strategy. CKS, PC, and DBI values were calculated for #Clusters=2 to #Clusters=8, and the error bars of PC were the standard deviations of five runs with random colors to label the regions. Both PC and DBI are so-called internal validation criteria, which suggested contradictory #clusters values: #Clusters=3 and 2 respectively. Our CKS is an external validation criterion that used the histology-segmentation as a reference and chose #Clusters=4 for the good multimodal consistency it resulted in.

In our multimodal fusion-based strategy, we generated another sequence (#Clusters=2 to 8) of segmentation results by clustering the HF spectra. Cohen's kappa score (CKS) between each pair of MSI- and histology-segmentation/clusterings were calculated and plotted as the green solid line in Figure 2. More details as to CKS in the Materials and Methods section and SI. The maximum CKS was obtained with #Clusters=4. In other words, setting #Clusters to 4 gave rise to better consistency between the segmentation independently produced by two distinct bio-imaging modalities, indicating better multimodally corroborated biological validity. This can be further supported by Figure S5(b), where four relatively well-segregated groups of data points were observed in the 3D UMAP embedding space of MSI data. Above all, #Clusters=4 was in good accordance with the ground truth of renal anatomy (as shown in Figure 3(a)): the four regions corresponded to the inner cortex, outer cortex, medulla, and pelvis, respectively. Our strategy is essentially an external validation criterion [22]: it integrates the bio-information from both molecular profiles and the histomorphological appearance of a tissue specimen, which makes it immune to the noise and artifacts that are unlikely to exist in both MSI and histology data, and thus provides a more objective and reliable approach to clustering/segmentation validation.

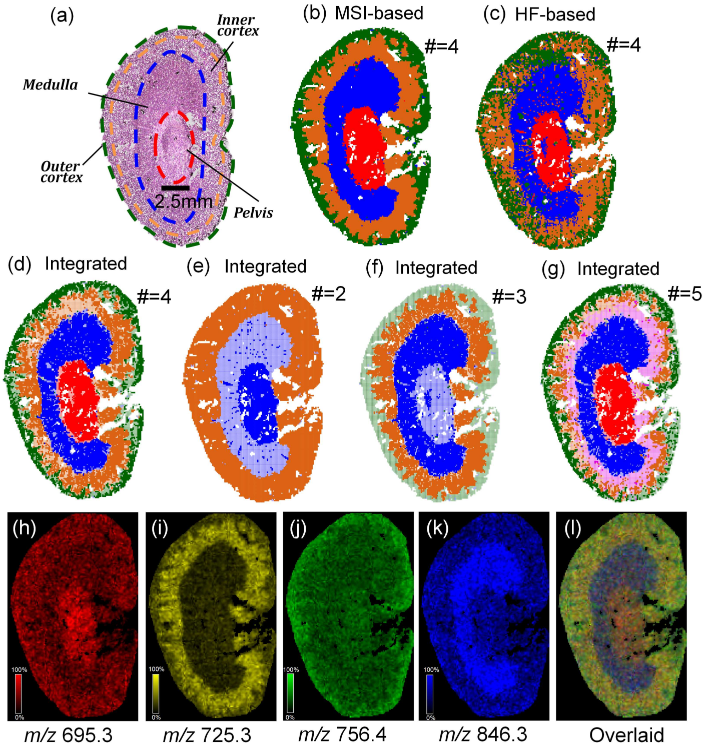

Figure 3. Tissue segmentation maps of a mouse kidney specimen: (a) the H&E image and anatomical structure, (b) MSI-segmentation for #Clusters=4, (c) histology-segmentation for #Clusters=4, and integrated maps for (d) #Clusters=4, (e) #Clusters=2, (f) #Clusters=3, (g) #Clusters=5. In the integrated maps, a pixel had a solid color if it was identically labeled by both the MSI- and histology-segmentation, otherwise, it would be half-transparent. It was clear that when #Clusters = 4, the regions got almost reproduced by the two independent bio-imaging modalities, which strongly supported their validity. (h)-(j) show the characteristic *m/z* maps of each ROI, which are overlaid in (k).

Integrated tissue segmentation

MSI- and histology-segmentation maps of the kidney specimen (Figure 3(a)) are shown in Figure 3(b) and (c) respectively. They were then integrated (Figure 3(d)) to produce a more informative and reliable ROI for the tissue section: (1) each foreground pixel was assigned a false color according to its MSI-based cluster; (2) for any pixel whose MSI- and HF-based cluster labels were inconsistent, its transparency was set to 20%. The holes in the middle right portion of the tissue section were the renal vein and artery. By marking those pixels that were labeled differently by different imaging modalities, we could visualize the confidence levels across the tissue segmentation: solid color indicated that the two modalities had consensus, so we could be more confident about the labeling, whereas transparent pixels indicated that the two modalities had dissensus, so we had to be cautious about the labeling. In Figure 3, integrated segmentation maps are shown for #Clusters equal to (d) 4, (e) 2, (f) 3, and (g) 5. In (b), pixels whose cluster label = 1 were assigned to red, label = 2 to blue, label = 3 to orange, and label = 4 to green. In (g), magenta was added to represent cluster label = 5. In line with Figure.2, #Clusters=4 resulted in the lowest proportion of inconsistently labeled (i.e. unconfident) pixels, suggesting regions best reproduced multimodally. Eventually, the 4 groups of confident pixels in (d) were returned as the ROIs of the whole kidney specimen, which related the spatial heterogeneity of chemical composition (detected by MSI) to that of histological morphology (detected by H&E image).

A closer look at those unconfident pixels in Figure 3(d) revealed that they very often appeared at the boundaries between the ROIs. There are two possible explanations for this: (1) each MSI pixel or H&E image tile had a size of 100×100 μm^2, which made it sort of a "mixture" of cells from two neighboring tissue regions and therefore harder to be segmented correctly; (2) the registration between MSI and HF data might fail to fully correct all global and local differences between the serial tissues, so the one-to-one mapping between MSI pixel and H&E tile was not perfectly accurate (for instance, the pelvis of the H&E staining tissue section might be slightly smaller than that of the MSI section and consequently the peripheral part of the red region was labeled differently by (b) and (c)).

Moreover, as shown in Figure 3(b), a number of red pixels (i.e. pelvis) appeared to be scattered in the blue and orange regions (i.e. medulla and inner cortex). Given the fact that such a layout was anatomically impossible, it was probably due to the so-called "salt and pepper noise" common to MSI data or due to certain artefacts incurred by the post-acquisition data processing and clustering analysis pipeline. Such incorrectly labeled pixels could be difficult to notice for specimens without well-known histoanatomical structures. But in Figure 3(d), the integrated segmentation map labeled the scattered red pixels as unconfident, which suggested that our multi-modal strategy was able to detect such histomorphologically-baseless labeling. Conventionally, MSI users resorted to spatial aware clustering approaches to handle such noise [13, 14]. Our strategy detected those misclassified pixels automatically and eliminated their potential negative influence on the following analysis by excluding them from ROI delineation. Conversely, the spatial aware approaches would force them to be assigned to a seemingly correct ROI and propagate their noise to the following analysis such as the calculation of the mean mass spectrum for a histology entity.

According to the integrated segmentation maps in Figure 3 (d-g), it was the different orders of merging regions that led to the lower CKS at #Clusters = 2 and 3 in Figure 2. For the MSI-segmentation, the medulla was merged with the pelvis at #Clusters = 2 and 3. This was different to the histology-segmentation which instead merged the medulla to the cortex at #Clusters = 2 and defined it as a separate region at #Clusters = 3. At #Clusters = 4, which coincided with the intrinsic number of major anatomical structures of a mouse kidney, both the MSI- and histology-segmentation par-

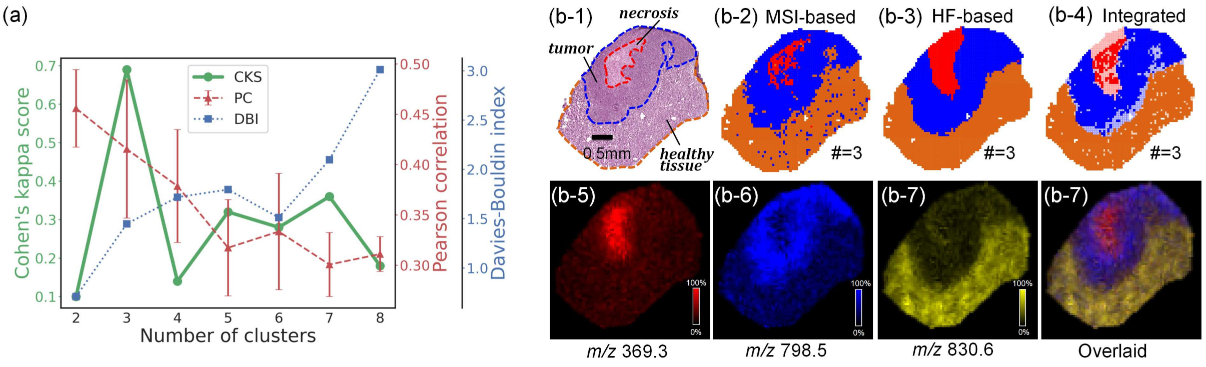

Figure 4. Application of our strategy on a renal adenocarcinoma tissue specimen from an orthotopic murine model: (a) CKS, PC, and PC curves to determine the most probable #Clusters, (b-1) the H&E image and pathological structures, (b-2) MSI-segmentation for #Clusters=3, (b-3) histology-segmentation for #Clusters=3, and (b-4) their integrated maps. Solid pixels in (b-4) were returned as automatically defined ROIs. (b-5)-(b-7) show the characteristic *m/z* maps of each ROI, which are overlaid in (b-8). The tissue section was harvested from the murine model's renal cortex, which was invaded by kidney cancer cells (ACHN) injected below its renal capsule.

titioned the tissue in a way matching up to those anatomical structures and therefore produced the highest CKS. When #Clusters=5, the magenta segment discovered by MSI had no counterpart in the histology-segmentation, suggesting that it could not be validated by histomorphology.

ROIs were defined using only the pixels of solid colors (i.e. confidently labeled pixels). Ions colocalized with each ROI were found by calculating the Pearson correlation between their spatial distributions and the binary mask of an ROI. As shown in Figure 3 (h) to (k) as well as in Figure S6, 695.3 *m/z*, 725.3 *m/z*, 756.4 *m/z*, and 846.3 *m/z* are presented as characteristic ions for each ROI respectively, which were tentatively identified as $[M+NH_4]^+$ of PC(28:0), $[M+Na]^+$ of SM(d34:1), $[M+H]^+$ of PC(34:3), and $[M+K]^+$ of PC(38:5) according to an organ-specific metabolite database built by Zhu et al [34]. Figure 3 (k) shows an overlay of (h) to (j): areas dominated by the four ions were reasonably well separated and could largely account for the segmentation map (d). In Figure S7, our automated ROI delineation strategy was applied to another adjacent kidney tissue slice of the same specimen, which produced similar results and thus confirmed its reproducibility and robustness.

Application on kidney tumor tissue

Tumor specimens are central to MSI-based preclinical researches [1]. To assess the suitability of our strategy to tumor specimens, we applied it to the renal cortex tissue of an orthotopic mouse model invaded by human renal adenocarcinomas [35]. Again, the pre-trained DCNN was used to produce histology-segmentation and the CKS curve was used to select #Clusters (the solid green line in Figure 4 (a) suggests #Clusters=3). Figure 4 (b) sums up the results for the tumor specimen: the MSI- (b-2) and histology- (b-3) segmentation were integrated into (b-4), where consistently and inconsistently labeled pixels were indicated in the same way as Figure 3. In Figure 4 (b-5 to b-7), 369.3 *m/z*, 798.5 *m/z*, and 830.6 *m/z* were selected through Pearson correlation as the characteristic ions for the red, blue, and orange ROIs respectively. It can be seen from Figure 4 (b-8), which was an overlay of (b-5) to (b-7), that area dominated by each characteristic ion coincided well with its corresponding ROI. In order to reveal the pathological nature of the three ROIs, we consulted a histologist: as shown in Figure 4(b-1), the red, blue, and orange areas were recognized as tumor necrotic zone, viable tumor, and healthy tissue, respectively. Tumor necrosis referred to tumor cell death in the core regions of solid tumors due to an accumulation of toxic waste products and a lack of oxygen and nutrient supply [36]. The three discriminant *m/z* discovered above were tentatively assigned as $[M+H-H_2O]^+$ of Cholesterol, $[M+K]^+$ of PC(34:1), and $[M+Na]^+$ of PC(38:5) respectively, which might be associated with possible metabolic/lipidomic alterations due to kidney tumor development.

By comparing the MSI- and histology-segmentation results, we became able to automatically and unbiasedly determine to which extent that molecular variation could be explained by underlying histomorphological variation. So there are two remarkable benefits of adopting our strategy during ROI delineation. (1) It can guide histologists' annotation towards appropriate granularity (i.e. the level of anatomical details). For instance, if the histologist had only partitioned our renal tumor specimen into 'tumor' and 'non-tumor' (i.e. delineated only two ROIs as suggested by PC and DBI in Figure 4(a)), then the tumor necrotic zone would have been labeled as 'tumor'. Following the conventional histology-guided protocol, we would have lost the opportunity to investigate the molecular difference between viable and necrotic tumors. (2) It can help lessen the burden of histologists because they only need to visually inspect a few H&E image excerpts from each HF-based region before confirming its pathological state (as in Figure S10). Otherwise, they would have to examine the large 2D whole slide image (10,000 to 100,000 pixels in each dimension) in an exhaustive manner and draw boundaries carefully around each recognized ROI. The tumor is one of the principal application areas of MSI, so confirming the suitability of our method to tumor specimens supports its practical value in future applications.

Conclusions

The present study is undertaken to develop an intelligent tool for the ROI delineation step of MSI data analysis and demonstrate its practical value with distinct types of tissue specimens. Our multi-modal fusion-based strategy provides an objective way to evaluate the validity of segmentation results. So we can compare the results obtained with different #Clusters and choose an optimal one, which assures that each ROI is orthogonally confirmed by both the molecular imaging modality and histology microscopy modality. Due to the unsupervised nature of the histology-segmentation method we proposed, it requires no more training and is applicable to all sorts of tissues without any specimen-specific adaption (no matter the species, organs, or pathological status). Although it may still be necessary to consult with histology experts when interpreting the biological nature of those ROIs, our method goes a long way towards guiding the histologists' annotation and reducing their workloads.

In addition, we also reported a multimodal registration method between the MSI and microscopy datasets (more in the Materials and Methods section), which implicitly used the HF data as a "virtual intermediate modality". Although integration with microscopy has been a routine part of MSI data analysis, registration between them can be a dilemma because of their disparate image

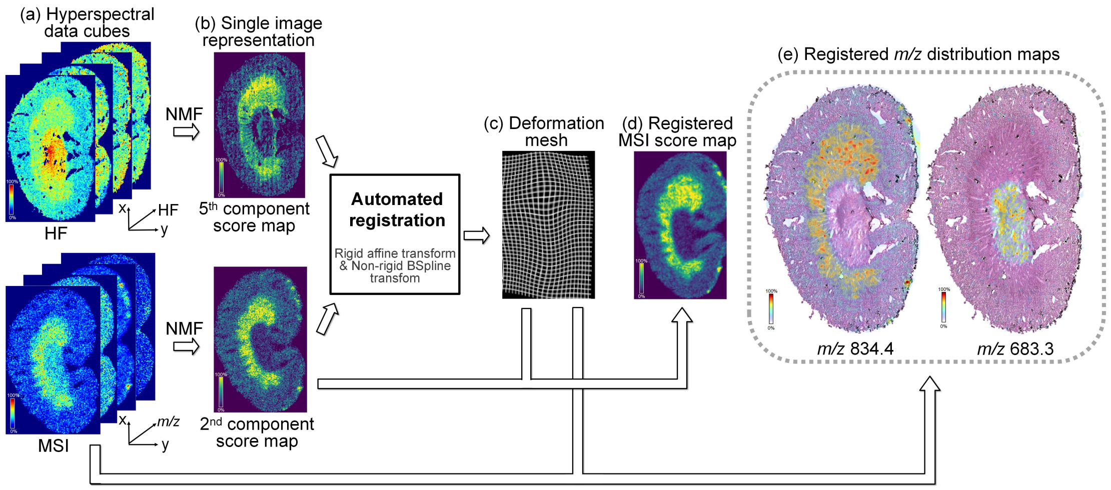

Figure 5. multimodal registration between HF and MSI data. (a) the hyperspectral data cubes of HF and MSI, (b) the score maps of the 5th and 2nd NMF components of the HF and MSI data, which were seen as their respective single image representations and used as inputs to registration algorithms, (c) deformation mesh to visualize the spatial transform output by registration algorithms, (d) the MSI score map was aligned to the HF data cube by the above transform, (e) ion maps of all *m*/*z* were registered, upsampled, and overlapped on top of the H&E image.

characteristics as discussed in [37]: the MSI of the mouse kidney shows narrow, spatially specific images in kidney regions but the H&E shows broad staining across the kidney and a combination of stain color and morphology is used to delineate regions. The HF data solved this problem by (i) encoding the stain color and morphology information of H&E into the HF data cube, (ii) extracting region-specific images from the HF data cube with NMF, and (iii) performing registration between the two manually-matched region-specific images (i.e. single image representations). So, the registration approach introduced in this paper has the potential to become a generic method for MSI and microscopy. But more work needs to be done to explore this idea further.

Our strategy is in principle applicable to other MSI ionization technologies such as Secondary Ion Mass Spectroscopy (SIMS) or MALDI (note that the software tool we provide is coded in Python, so the data format output by MSI instruments must be Python compatible, e.g. imzML), but follow-up studies are required to test its genericity. In particular, MALDI and SIMS can achieve lateral resolutions greater than 10μm, in which case we anticipate that a more powerful spatial registration approach compatible with the single cell scale will be required to establish accurate correspondence between MSI pixel and H&E tile. Combining our method with high spatial resolution MSI data may further improve the granularity and accuracy of the spatial segmentation results. For example, the "unconfident pixels" at the borders between ROIs (Figure.3(d) and Figure.4(b-4)) may be subdivided and "deconvolved", resulting in their assignment to existing clusters or the creation of new clusters.

Finally, in our multimodal fusion strategy, other imaging modalities can be used instead of optical microscopy. As an alternative method to obtain a label-free distribution of molecular species within a sample, vibrational spectroscopic imaging techniques, such as Fourier Transform Infrared (FTIR) and Raman imaging, capture information about chemical bonds, making them complementary to MSI and great candidates for multimodal corroboration of spatial segmentation. Specifically, we can replace the HF spectra extracted from each tile with corresponding FTIR or Raman spectra, and the rest of our strategy remains essentially the same.

Materials and Methods

Sample Preparation and Data Acquisition

The animal experiments were approved by the Animal Care and Use Committee of Shenzhen Institute of Advanced Technology, Chinese Academy of Sciences. A mouse kidney (7-month age female C57) was frozen-preserved at -80° before being cut into two consecutive slices by cryostat sectioning with a thickness of 20 μm and 10 μm respectively. MSI was done on the thicker slice with a 100 $\times$ 100 μm step size (i.e. pixel resolution) using a Desorption Spray Ionization (DESI) ion source (Prosolia, US) coupled to a Synapt G2-Si mass spectrometer (Waters, UK). HPLC grade methanol (Merck, Germany) was used in DESI experiments at a flow rate of 1.5 μl/min. In the positive ion mode, the capillary voltage of DESI was 4.2 kV with a sampling cone of 80 V. The nebulizing nitrogen gas pressure of DESI was 0.5 MPa and the ion transfer capillary was at a temperature of 150 °*C*. A mass range of 200 - 1,000 *m*/*z* was covered with a mass resolving power of 10,000. The thinner slice went though standard Hematoxylin and Eosin (H&E) staining protocol, followed by digital pathology scanning with 20$\times$ magnification (i.e. 0.5 $\times$ 0.5 μm pixel resolution). A murine model of human renal adenocarcinoma was established by orthotopically transplanting the ACHN cell line (Zhuhai BesTest Bio-Tech, China) into NOD/SCID male mice at 4 weeks of age and growing for 5 more weeks before sacrifice. Being capable of mimicking the organ-specific tumor environment in humans, orthotopic models have been routinely used in the MSI community for understanding the biomolecular mechanisms underlying cancers and for investigating therapeutic agents [38]. The same experimental procedures were followed for the tumor specimen, except that the pixel resolution of MSI was set to 50 $\times$ 50 μm^2 in adaptation to a small tumor and surrounding tissue area.

Data processing and analysis

MSI and H&E data preprocessing

Raw MSI data were preprocessed with R Cardinal 2 package [39] following standard data preprocessing protocols, including total-ion-count normalization, spectral smoothing, baseline reduction, peak picking, peak alignment, peak binning, and peak filtering. Pixels were classified as "foreground" or "background" (i.e. located inside or outside the tissue section) by applying thresholding on their sums of intensities of selected tissue-specific ions. We excluded ions that had stronger signals in the background than in the foreground because they appeared to be noisy and not biologically

informative. Raw H&E images were preprocessed using standard protocols including tissue detection and color normalization (HistomicsTK 1.2.3 package [40]). Then, for the kidney experiments, the whole slide image was uniformly split into an array of image tiles and each tile had a size of 200 × 200 pixels. Notably, the pixel size of MSI was 200 times larger than that of the H&E image (100 μm/0.5 μm), so each image tile had the same physical size as an MSI pixel. For the tumor specimen, the size of a H&E image tile was set to 100 × 100 pixels, in line with the smaller MSI pixel size (50 × 50 μm^2). Tiles containing a minimum tissue area no less than 90% were classified as "foreground" and others were "background". For the whole kidney specimen, all foreground MSI pixels and H&E image tiles were used for following analysis; for the tumor specimen, only the tumor area and its surroundings were used for following analysis to reduce computational cost. All data processing and analysis programs were implemented using Python 3.7 unless otherwise specified.

Encoding an H&E image tile into a HF spectrum

"Features" are a series of measurable properties to fully characterize an object in a data set. For MSI data, it is rather straightforward that *m/z* values are the features sufficient to convey molecular information of each spatial pixel. However, how to construct features to encode the histology appearance of a H&E image isn't as self-evident. In 31, Mormont et al. thoroughly investigated the performance of different DCNN architectures as off-the-shelf feature extractors for a group of histology and cytology object classification tasks. All DCNN were pre-trained using the ImageNet database [30]. An inner layer (conv5-block32-concat) of DenseNet 201 [41] empirically achieved the highest average performance in the tasks and thus stood out as our extractor of choice to encode the stain color and morphology of a 2D H&E image tile into a set of informative HF. To stay in keeping with ImageNet, our input tiles had to be resized to [224, 224, 3] through bilinear interpolation and each color channel had to be centered to zero. They were then forward propagated through a sequence of densely connected convolutional layers, where about 8.6 Giga FLOPs of calculation took place. Eventually, the output arrays after the concatenation operation at the conv5_block32 layer of DenseNet201 were 2-D globally average-pooled, Min-Max scaled, and reshaped as a "histomorphological feature (HF) spectrum" of 1,920 variables. The HF spectra of all the tiles were formatted into a hyperspectral data cube like MSI (Figure 1): each tile became a pixel and each (foreground) pixel had an HF spectrum. Image processing were implemented by OpenCV 4.2.0.32 and the pre-trained DCNN was downloaded from Keras 2.4.3.

Spatial registration between MSI and HF data

Registration was a prerequisite to fusing multimodal imaging data sets, where the MSI and HF data cubes were spatially aligned. We used a dimension reduction algorithm Nonnegative Matrix Factorization (NMF) to generate single image representations for the data cubes (as shown in Figure.5): (1) NMF reduced the data cubes into two sets of score maps, (2) we manually selected two score maps whose spatial expression best matched each other (see Figure S3) and used them as the inputs of following intensity-based registration algorithms. DESI-MSI preferred a section thickness of >20 μm to produce intense ion signals, which was above the recommended thickness for H&E staining microscopy (<10 μm). So, we had to use serial sections, one for MSI and one for H&E. This meant that there might be nonlinear deformation between the MSI and HF score maps due to inter-section anatomical variations or inconsistent sample preparation artifacts. Hence, we combined a linear registration algorithm (affine transform) with a nonlinear one (BSpline-based Free Form Deformation) to both align the score maps globally and correct local deformations. Mutual Information was used as the image similarity metric to guide the gradient descent-based optimization of linear and non-linear transforms. Finally, we applied the optimized transforms to all ion maps (*m/z* channels). To evaluate registration quality, two representatives of registered ion maps (*m/z* 834.4 and 683.3) were upsampled 200 times (spline interpolation of 3 order) and overlaid on top of H&E image in Figure.5(e). Figure.S12 shows a visual assessment of the registration quality: good overlap between the HF-derived and MSI-derived tissue masks, and between the distinct anatomical features observed in both HF and MSI images, indicate good registration results. More about the registration process in *SI*. Scikit-learn 0.24.2, Scikit-image 0.18.1, and SimpleITK 2.1.0 libraries were used to implement the above algorithms.

Segmentation and multimodal fusion-based evaluation

Spectral clustering is a graph-based clustering algorithm reported to outperform traditional algorithms such as k-means in MSI data-based tissue segmentation [42]. So, we used spectral clustering to group foreground pixels based on their HF and mass spectral similarities respectively (more details in *SI*) and obtained two sets of clustering/segmentation results (i.e. two sets of labels for each pixel). Then, CKS [43] was employed to compare the two sets of results: perfect agreement gave a CKS of 1.0 and random labeling gave a CKS close to 0.0. The mathematical definition of CKS is given in the *SI*. By taking into account the possibility of the agreement occurring by chance, CKS is not biased towards small #Clusters and hence a better measure than simple percentage agreement calculation. Note that the labeling of the clusters/regions between the two modalities was aligned by finding the largest CKS of all possible permutations. So, for a given #Clusters, CKS evaluated the biological validity of MSI-segmentation by how much it was reproduced by histology-segmentation. Figure S1 provides a step-by-step illustration of the entire methodology.

Availability of source code and requirements

Source codes from this work are freely accessible at https://github.com/guoang4github/ROIforMSI/ (Licence: GPL-3). Our software tool is also registered in the bio.tools (roiformsi) and SciCrunch (RRID:SCR_023275) databases. Computational workflow is registered in workflowhub.eu [44].

Availability of supporting data and materials

The MSI data have been deposited to the ProteomeXchange Consortium (http://proteomecentral.proteomexchange.org) via the iProX partner repository [45] with the dataset identifier PXD038876. Snapshots of our code and other data further supporting this work are openly available in the GigaScience repository, GigaDB [46].

Declarations

List of abbreviations

MSI: Mass spectrometry imaging; ROI: Region-of-interest; H&E: Haemotoxylin and Eosin; DCNN: Deep convolutional neural network; HF: histomorphological features; PC: Pearson correlation; CKS: Cohen's kappa score; DBI: Davies–Bouldin index; Nonnegative Matrix Factorization (NMF); MALDI: Matrix-Assisted Laser Desorption Ionization; DESI: Desorption electrospray ionization; UMAP: Uniform manifold approximation and projection

Ethical Approval

The animal experiments were approved by the Animal Care and Use Committee of Shenzhen Institute of Advanced Technology, Chinese Academy of Sciences.

Consent for publication

Not applicable

Competing Interests

The author(s) declare that they have no competing interests

Funding

This study was financially supported by National Natural Science Foundation of China (82127801 and 22076197), National Key R&D Program of China (2022YFF0705003), Shenzhen Science and Technology Innovation Commission (JCYJ20200109115405930, JCYJ20210324115811031), Guangdong Basic and Applied Basic Research Foundation (2020B1515120080, 2021A1515110096), the Innovation Program for Excellent Young Researchers of Shenzhen Institute of Advanced Technology, the Open Research Program of Guangzhou Life Sciences Facility Center of the Chinese Academy of Sciences (GZQY202004).

Author's Contributions

A.G., F.L., and Q.L. designed research; A.G.and Z.C. performed research; A.G. analyzed data; and A.G. and Q.L. wrote the paper.

Acknowledgements

We thank Dr.Yueguang Lv for his comments that significantly improved this manuscript.

References

1. McDonnell LA, Heeren RMA. Imaging mass spectrometry. Mass Spectrometry Reviews 2007;.
2. Norris JL, Caprioli RM. Imaging mass spectrometry: A new tool for pathology in a molecular age. Proteomics Clinical Applications 2013;7(11-12):733–738.
3. Chaurand P, Schwartz SA, Caprioli RM. Imaging mass spectrometry: a new tool to investigate the spatial organization of peptides and proteins in mammalian tissue sections. Current Opinion in Chemical Biology 2002;6(5):676–681.
4. Seeley EH, Caprioli RM. Molecular Imaging Of Proteins In Tissues By Mass Spectrometry. Proceedings of the National Academy of Sciences of the United States of America 2008;105(47):18126–18131.
5. Cole LM, Clench MR. Mass spectrometry imaging tools in oncology. Biomarkers in medicine 2015;9(9):863–868.
6. Schnackenberg LK, Thorn DA, Barnette D, Jones EE. MALDI imaging mass spectrometry: An emerging tool in neurology. Metabolic Brain Disease 2021;p. 1–17.
7. Watrous JD, Dorrestein PC. Imaging mass spectrometry in microbiology. Nature Reviews Microbiology 2011;9(9):683–694.
8. Nilsson A, Goodwin RJ, Shariatgorji M, Vallianatou T, Webborn PJ, Andrén PE. Mass spectrometry imaging in drug development. Analytical chemistry 2015;87(3):1437–1455.
9. Thomas A, Patterson NH, Marcinkiewicz MM, Lazaris A, Metrakos P, Chaurand P. Histology-driven data mining of lipid signatures from multiple imaging mass spectrometry analyses: application to human colorectal cancer liver metastasis biopsies. Analytical chemistry 2013;85(5):2860–2866.
10. Verbeeck N, Caprioli RM, de Plas RV. Unsupervised machine learning for exploratory data analysis in imaging mass spectrometry. Mass Spectrometry Reviews 2020;.
11. Mccombie G, Staab D, Stoeckli M, Knochenmuss R. Spatial and Spectral Correlations in MALDI Mass Spectrometry Images by Clustering and Multivariate Analysis. Analytical Chemistry 2005;77(19):6118–6124.
12. Deininger S, Ebert M, Futterer A, Gerhard M, Rocken C. MALDI imaging combined with hierarchical clustering as a new tool for the interpretation of complex human cancers. Journal of Proteome Research 2008;7(12):5230–5236.
13. Alexandrov T, Becker M, Deininger S, Ernst G, Wehder L, Grasmair M, et al. Spatial segmentation of imaging mass spectrometry data with edge-preserving image denoising and clustering. Journal of Proteome Research 2010;9(12):6535–6546.
14. Kobarg JH. Efficient spatial segmentation of large imaging mass spectrometry datasets with spatially aware clustering. Bioinformatics 2011;27(13):p.230–238.
15. Inglese P, Mckenzie JS, Mroz A, Kinross J, Veselkov K, Holmes E, et al. Deep learning and 3D-DESI imaging reveal the hidden metabolic heterogeneity of cancer. Chemical Science 2017;8(5):3500–3511.
16. Delcourt V, Franck J, Leblanc E, Narducci F, Robin YM, Gimeno JP, et al. Combined mass spectrometry imaging and top-down microproteomics reveals evidence of a hidden proteome in ovarian cancer. EBioMedicine 2017;21:55–64.
17. Song X, He J, Pang X, Zhang J, Sun C, Huang L, et al. Virtual calibration quantitative mass spectrometry imaging for accurately mapping analytes across heterogenous biotissue. Analytical chemistry 2019;91(4):2838–2846.
18. Jones MA, Cho SH, Patterson NH, Van de Plas R, Spraggins JM, Boothby MR, et al. Discovering new lipidomic features using cell type specific fluorophore expression to provide spatial and biological specificity in a multimodal workflow with MALDI Imaging Mass Spectrometry. Analytical chemistry 2020;92(10):7079–7086.
19. Taylor AJ, Dexter A, Bunch J. Exploring ion suppression in mass spectrometry imaging of a heterogeneous tissue. Analytical chemistry 2018;90(9):5637–5645.
20. Bemis KD, Harry A, Eberlin LS, Ferreira CR, Vitek O. Probabilistic segmentation of mass spectrometry images helps select important ions and characterize confidence in the resulting segments. Molecular Cellular Proteomics Mcp 2016;15(5):mcp.O115.053918.
21. Abdelmoula WM, Balluff B, Englert S, Dijkstra J, Reinders MJT, Walch A, et al. Data-driven identification of prognostic tumor subpopulations using spatially mapped t-SNE of mass spectrometry imaging data. Proceedings of the National Academy of Sciences of the United States of America 2016;113(43):12244–12249.
22. Liu Y, Li Z, Xiong H, Gao X, Wu J. Understanding of internal clustering validation measures. In: 2010 IEEE International Conference on Data Mining IEEE; 2010. p. 911–916.
23. Moulavi D, Jaskowiak PA, Campello RJ, Zimek A, Sander J. Density-based clustering validation. In: Proceedings of the 2014 SIAM international conference on data mining SIAM; 2014. p. 839–847.
24. Neumann EK, Djambazova KV, Caprioli RM, Spraggins JM. Multimodal imaging mass spectrometry: next generation molecular mapping in biology and medicine. Journal of the American Society for Mass Spectrometry 2020;31(12):2401–2415.
25. Van de Plas R, Yang J, Spraggins J, Caprioli RM. Image fusion of mass spectrometry and microscopy: a multimodality paradigm for molecular tissue mapping. Nature methods 2015;12(4):366–372.
26. Rappez L, Stadler M, Triana S, Gathungu RM, Ovchinnikova K, Phapale P, et al. SpaceM reveals metabolic states of single cells. Nature methods 2021;18(7):799–805.
27. LeCun Y, Bengio Y, Hinton G. Deep learning. nature 2015;521(7553):436–444.
28. Coudray N, Ocampo PS, Sakellaropoulos T, Narula N, Snuderl

M, Fenyö D, et al. Classification and mutation prediction from non–small cell lung cancer histopathology images using deep learning. Nature medicine 2018;24(10):1559–1567.
29. Weiss K, Khoshgoftaar TM, Wang DD. A survey of transfer learning. Journal of Big Data 2016;3(1):9.
30. ImageNet Large Scale Visual Recognition Challenge. International Journal of Computer Vision 2015;.
31. Mormont R, Geurts P, Maree R. Comparison of Deep Transfer Learning Strategies for Digital Pathology. In: 2018 IEEE/CVF Conference on Computer Vision and Pattern Recognition Workshops (CVPRW); 2018. .
32. McInnes L, Healy J, Melville J. Umap: Uniform manifold approximation and projection for dimension reduction. arXiv preprint arXiv:180203426 2018;.
33. Smets T, Verbeeck N, Claesen M, Asperger A, Griffioen G, Tousseyn T, et al. Evaluation of distance metrics and spatial autocorrelation in uniform manifold approximation and projection applied to mass spectrometry imaging data. Analytical chemistry 2019;91(9):5706–5714.
34. Zhu Y, Zang Q, Luo Z, He J, Zhang R, Abliz Z. An Organ-Specific Metabolite Annotation Approach for Ambient Mass Spectrometry Imaging Reveals Spatial Metabolic Alterations of a Whole Mouse Body. Analytical Chemistry 2022;94(20):7286–7294.
35. Robert, J, Motzer, Neil, H, Bander, et al. Renal-Cell Carcinoma. New England Journal of Medicine 1996;.
36. Vakkila J, Lotze MT. Inflammation and necrosis promote tumour growth. Nature Reviews Immunology 2004;4(8):641–648.
37. Tuck M, Blanc L, Touti R, Patterson NH, Van Nuffel S, Villette S, et al. Multimodal imaging based on vibrational spectroscopies and mass spectrometry imaging applied to biological tissue: a multiscale and multiomics review. Analytical chemistry 2020;93(1):445–477.
38. Goodwin R, Bunch J, McGinnity D. Mass spectrometry imaging in oncology drug discovery. Advances in cancer research 2017;134:133–171.
39. Kyle, D, Bemis, April, Harry, Livia, et al. Cardinal: an R package for statistical analysis of mass spectrometry-based imaging experiments: Fig. 1. Bioinformatics 2015;.
40. Cooper L. HistomicsTK; 2016, https://github.com/DigitalSlideArchive/HistomicsTK.
41. Huang G, Liu Z, Van Der Maaten L, Weinberger KQ. Densely connected convolutional networks. In: Proceedings of the IEEE conference on computer vision and pattern recognition; 2017. p. 4700–4708.
42. Dexter A, Race AM, Steven RT, Barnes JR, Hulme H, Goodwin RJ, et al. Two-phase and graph-based clustering methods for accurate and efficient segmentation of large mass spectrometry images. Analytical chemistry 2017;89(21):11293–11300.
43. McHugh, Mary, L. Interrater reliability: the kappa statistic. Biochemia Medica 2012;.
44. Guo A, Chen Z, Li F, Luo Q. Delineating Regions-of-interest for Mass Spectrometry Imaging by Multimodally Corroborated Spatial Segmentation. WorkflowHub; 2023, https://doi.org/10.48546/WORKFLOWHUB.WORKFLOW.437.1.
45. Ma J, Chen T, Wu S, Yang C, Bai M, Shu K, et al. iProX: an integrated proteome resource. Nucleic acids research 2019;47(D1):D1211–D1217.
46. Guo A, Chen Z, Li F, Luo Q. Supporting data for "Delineating Regions-of-interest for Mass Spectrometry Imaging by Multimodally Corroborated Spatial Segmentation". GigaScience Database; 2023, http://dx.doi.org/10.5524/102374.

Figure 1

Click here to access/download;Figure;(1) overview_revised.jpg

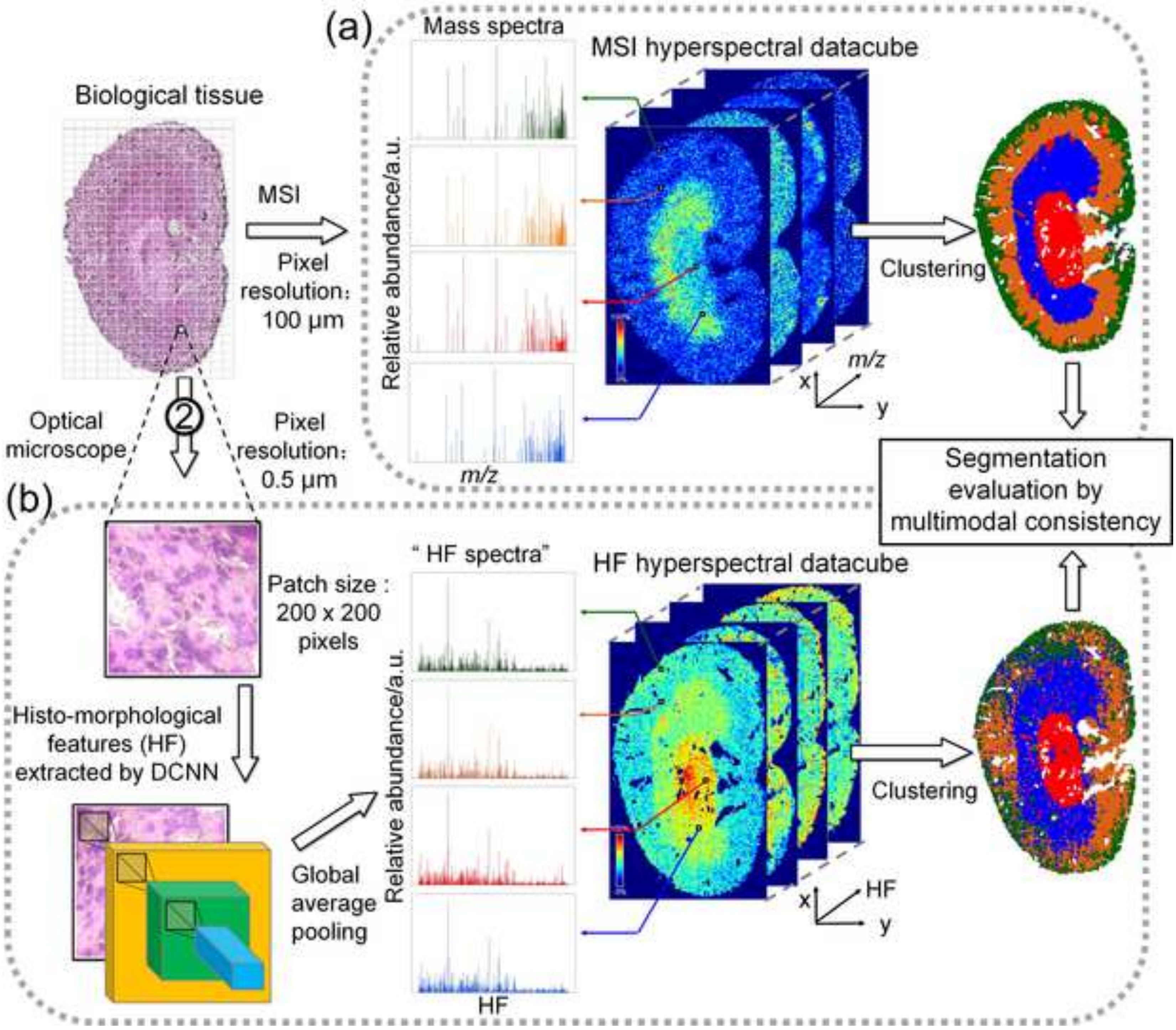

Click here to access/download;Figure;(4)
kidney_segments_revised.jpg

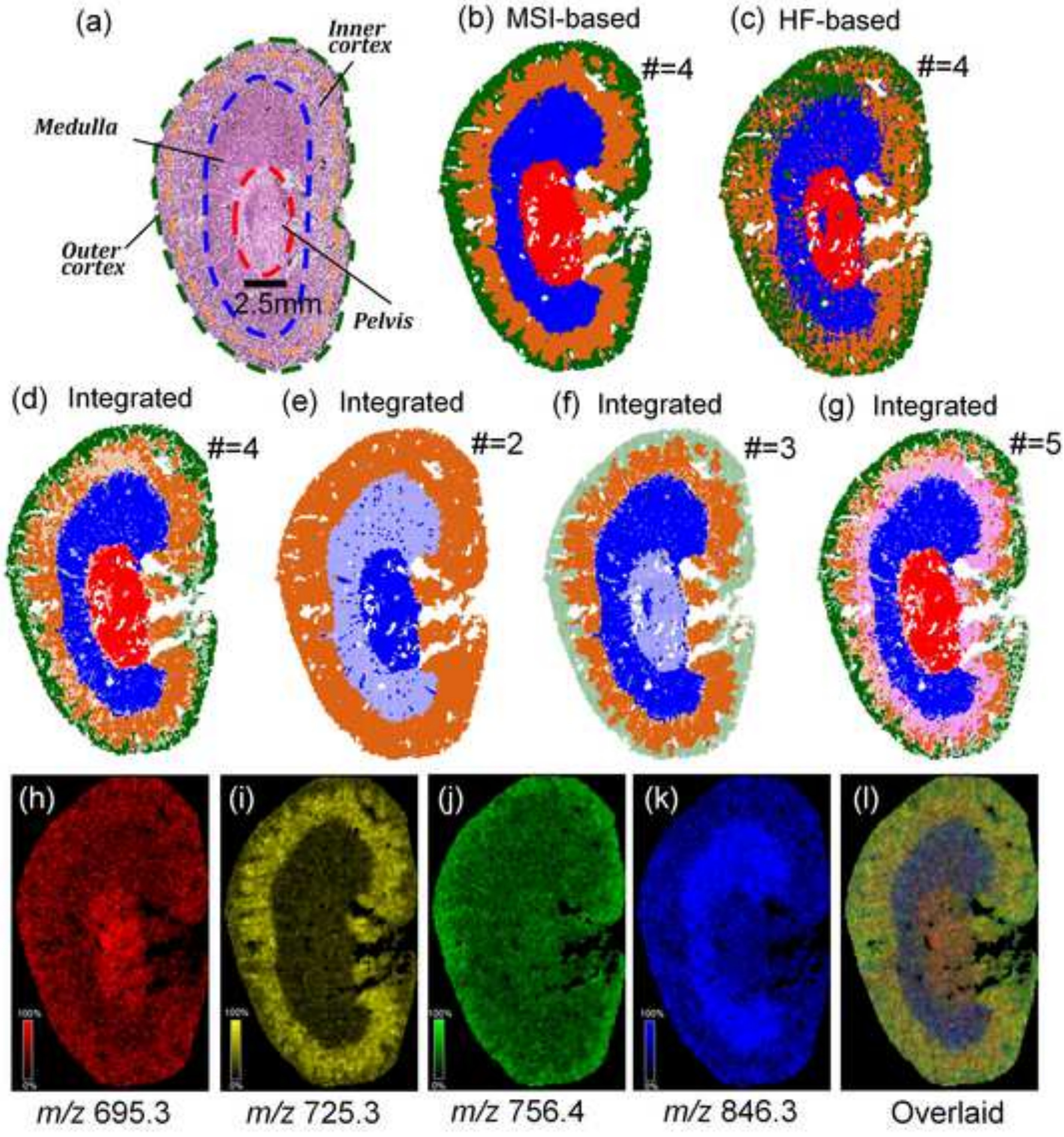

Click here to access/download;Figure;(3) No_of_clusters_plot_revised.jpg

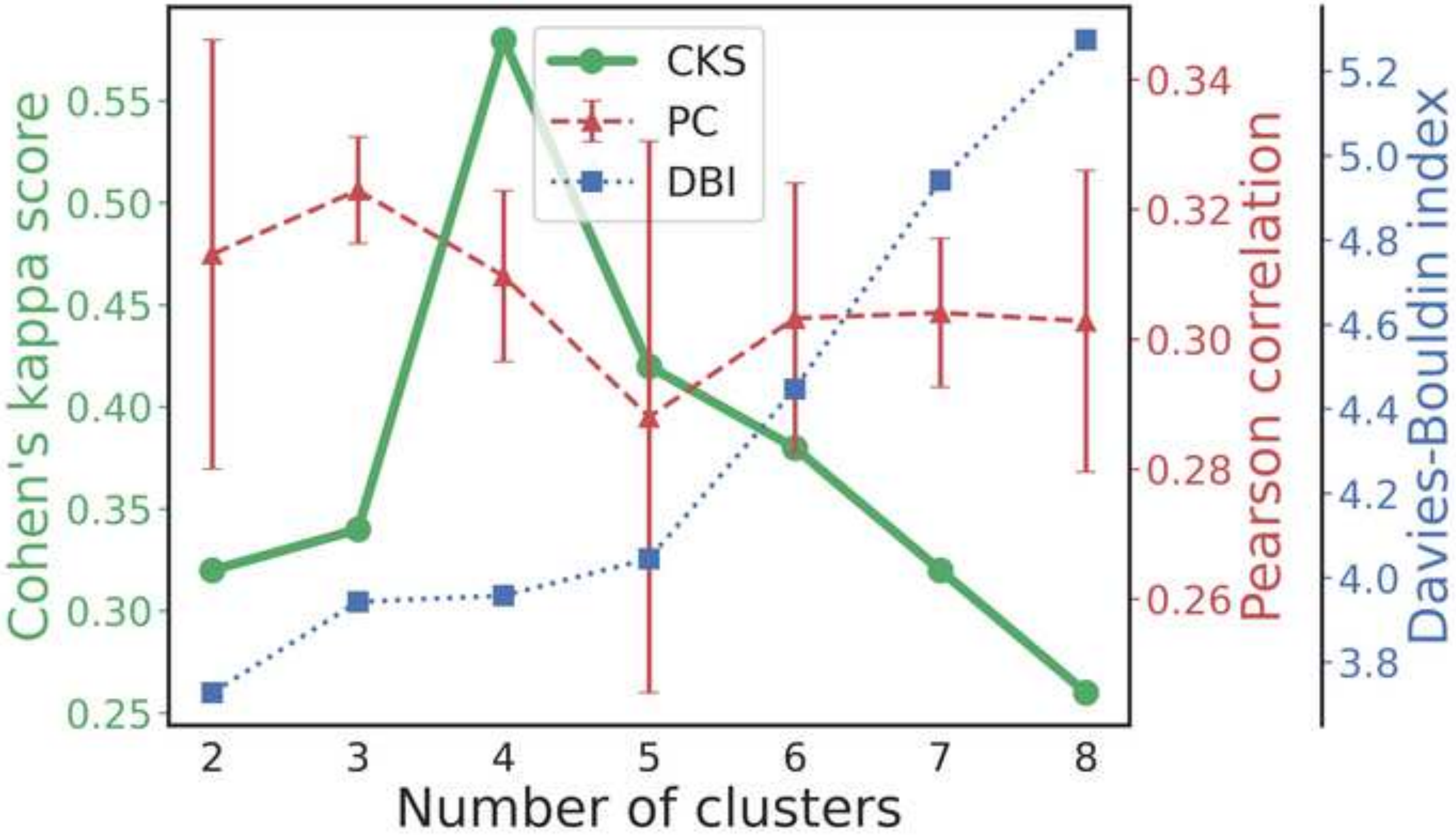

Figure 2

Click here to access/download;Figure;(2) kidney_registration_revised.jpg

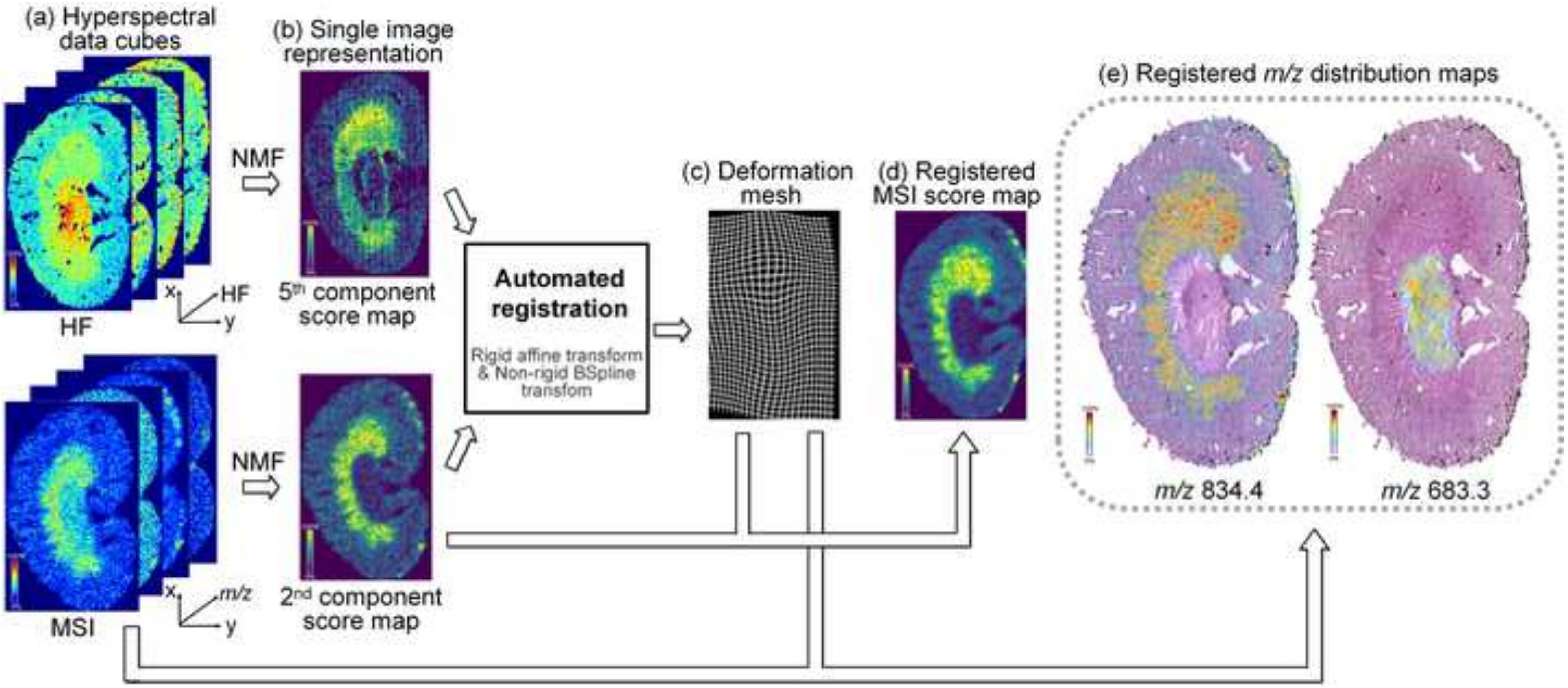

Figure

Click here to access/download;Figure;(5) tumor_segments.jpg

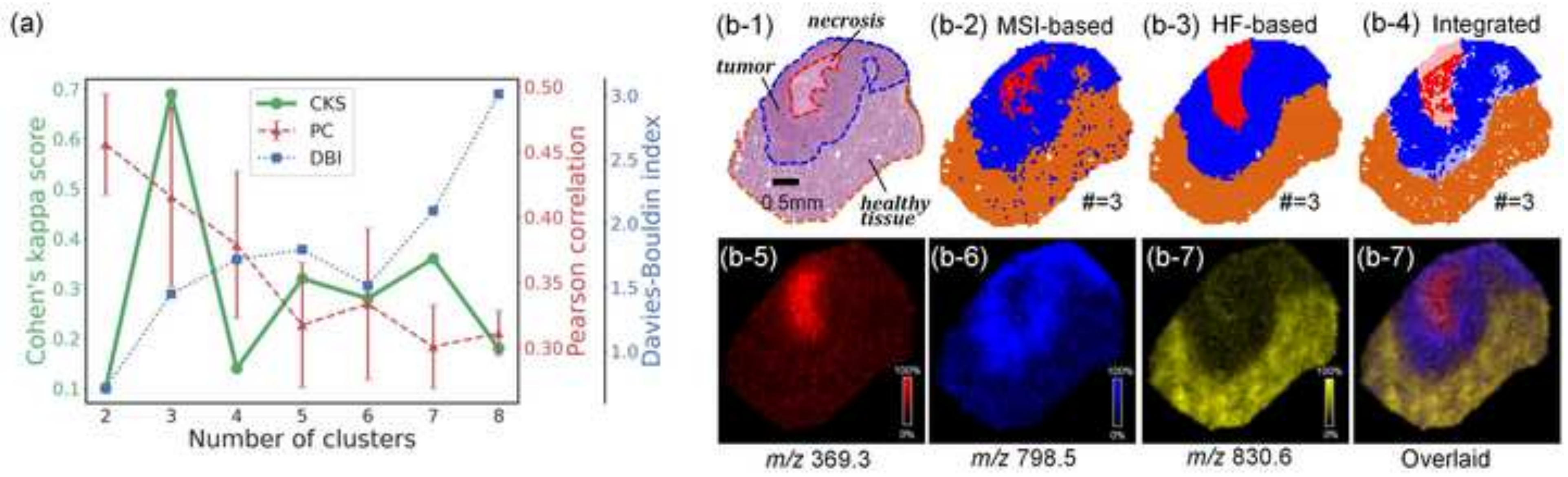

supplementary texts and figures

Click here to access/download
Supplementary Material
GigaScience_Supporting_Information_revised_230209.pdf

Cover letter

Click here to access/download;Personal Cover;cover letter.docx

Dear Editor:

I am writing to submit our manuscript entitled, "**Delineating Regions-of-interest for Mass Spectrometry Imaging by Multimodally Corroborated Spatial Segmentation**" for consideration as a research article for *GigaScience*.

Mass Spectrometry Imaging (MSI) is a tag-free molecular imaging technique for biological samples. It is capable of simultaneous localizing tens to hundreds of biomolecules in a single experiment by acquiring a full mass spectrum in each pixel of a virtual grid. In MSI data analysis, spatial segmentation, obtained by clustering MSI pixels according to their mass spectral similarities, is a popular approach to delineate Regions-of-Interest (ROIs) that correspond to tissue areas of different anatomical or pathological labels. However, there is currently no good strategy to select the number of clusters (#Clusters), which is the most important parameter for clustering algorithms and determines the granularity of the segmentation. Wrong #Clusters may lead to ROIs that are not biologically real. Here, we propose a novel multimodal fusion strategy that uses information from segmentation of a histopathology image to inform the task of segmentation of an MSI image. (i) a Deep Learning-based method is proposed to produce histology-segmentation from H&E images; (ii) the most probable #Clusters is determined by using the consistency between histology- and MSI- segmentations as a quantitative measure of biological validity. Our strategy has the merits of being objective and rigorous and ensures that its produced ROIs are of biological relevance supported by both MSI and histology.

Given that MSI is being widely used in various areas of life sciences and that segmentation-based ROI delineation is an important step of MSI data analysis, we believe that the method presented in our paper will appeal to a broad range of readers who are current or potential MSI users. In addition, more general readers, who are interested in interdisciplinary research that applies Artificial Intelligence in life sciences, may also find our work valuable.

We have published a subset of our findings in a preprint repository BioArxiv in 2020 (doi: https://doi.org/10.1101/2020.07.17.208025). Part of the MSI data has been used in another article ("Multimodal Coregistration and Fusion between Spatial Metabolomics and Biomedical Imaging", in submission) but for entirely different research purposes.

Each named author has approved the contents of this paper, agreed to the *GigaScience*'s submission policies, and substantially contributed to conducting the underlying research and drafting this manuscript. Additionally, to the best of our knowledge, the named authors have no conflict of interest, financial or otherwise.

Sincerely,

Luo Qian
Ph.D. Professor
Institute of Biomedicin and Biotechnology,
Shenzhen Institute of Advanced Technology, Chinese Academy of Science.
E-mail:qian.luo@siat.ac.cn
