# Supplementary material for: Delineating regions of interest for mass spectrometry imaging by multimodally corroborated spatial segmentation
Source: Gigascience. 2023 Apr 11;12:giad021. doi: 10.1093/gigascience/giad021 (PMC10087011; doi:10.1093/gigascience/giad021)
Supplement: giad021_Supplemental_File [file giad021_supplemental_file.pdf]

Supporting Information: Delineating Regions-of-interest for Mass Spectrometry Imaging by Multimodally Corroborated Spatial Segmentation

Ang Guo,† Zhiyu Chen,†,‡ Fang Li,† and Qian Luo*,†,‡

†*Institute of Biomedicine and Biotechnology, Shenzhen Institutes of Advanced Technology, Chinese Academy of Sciences, Shenzhen 518055, China*

‡*University of Chinese Academy of Sciences, Beijing 100049, China*

E-mail: qian.luo@siat.ac.cn

Phone: +86 86392421

Supporting Information Available

Computating Equipments

All the data processing and analysis work, including the computation of DCNN extracted features, were performed using an AMAX workstation with two Intel Xeon Silver 4110 (2.10GHZ) CPUs, 192 GB RAM, and two NVIDIA GeForce RTX 2080Ti (11 GB) GPUs.

MSI Data Preprocessing

Details of raw MSI processing. (1) Total-ion-count (TIC) normalization projects spectra of varying intensity onto a common intensity scale and thus alleviates the experimentally

introduced pixel-to-pixel variation of MSI data. The spectral profile of each pixel is scaled based on an assumption that the TIC collected on every pixel should be identical. (2) Spectral smoothing is obtained with a Gaussian kernel (window = 5 and standard deviation = window/4). (3) Baseline reduction algorithm interpolates a baseline from local minima and subtracts it from the original spectral profile. (4) Peak picking (aka peak detection) identifies meaningful m/z peaks by seeking local maxima above certain predefined signal-to-noise (SNR) threshold (SNR = 6) in a sliding window (window width=5). In our case, the adaptive noise is estimated by local mean absolute deviations (MAD). (5) Peak alignment, in order to eliminate tiny m/z-value shifts due to instabilities of MS instruments, peaks with proximate m/z-values are matched given a tolerance threshold (200 ppm). (6) Peak binning, the intensity of a selected peak is represented with the sum of intensities between the two nearest local minima in both directions around its m/z value. (7) Peak filtering calculates the proportion of pixels where a peak is detected at a given m/z value and only retains peaks with frequencies greater than 1%. Above MSI data processing procedures were done with an R package named Cardinal 2.[?]

H&E Stained Histological Image Data Preprocessing

Tissue detection was performed for the digital H&E image to generate a binary mask that delineated the area occupied by the kidney tissue. Reinhard stain normalization method[S1] was subsequently used to transfer the color characteristics of the tissue area to the desired standard in order to correct any staining or imaging variations. Both the preprocessing procedures described above were realized using HistomicsTK package.[S2]

Mapping Between MSI pixels and H&E Image tiles

(1) The selected NMF score map of MSI data was set as a moving image and that of HF data was set as a fixed image. (2) In the linear global registration stage, an affine transform matrix, which maximized the similarity metric Mattes Mutual Information (number of histogram

bins = 10), was generated by gradient decent algorithm (learning rate = 1e-3, number of iterations = 200). We used a multi-resolution (3 levels) framework to accelerate convergence, where shrink factors per level were [4,2,1] and smoothing sigmas per level were [2,1,0]. (3) In the non-linear local registration stage, the BSpline-based Free Field Deformation algorithm (transform domain mesh size = [1, 1], order=3) was employed in a multi-resolution (4 levels) framework (shrink factors per level = [8,4,2,1] and smoothing sigmas per level = [4,2,1,0]). Similarity metrics were again set as Mattes Mutual Information (number of histogram bins = 50), but the optimizer was LBFGS2 (solution accuracy=1e-4, number of iterations=2000, delta convergence tolerance=1e-3). Spatial registration algorithms were implemented using the SimpleITK library.

Cohen's Kappa Score

Cohen's kappa score (CKS) quantifies the agreement between two independent raters in their way to categorize items into mutually exclusive classes. Its formula is as follows:

$$CKS = \frac{p_0 - p_e}{1 - p_e} = 1 - \frac{1 - p_0}{1 - p_e} \tag{1}$$

where p_0 is the observed agreement among raters, and p_e is the expected agreement by chance. $CKS = 1$ when the two raters are in complete agreement. $CKS = 0$ when agreement among the raters is purely by chance.

Feature Construction For Histology Image

The hierarchical structure of DCNN fully exploits the compositional nature of images, where higher-level features are obtained by combining lower-level ones: local edges assemble into motifs, motifs form parts, and parts form objects.[S3,S4] Therefore, the features extracted by deeper convolutional layer correspond to complex and abstract vision concepts, which are distinct from conventional handcrafted CV features. The reason for why inner layers features

were favored in S5 over final and shallow ones is that those features extracted by the final layer may be too specific to the original object recognition task of ImageNet and thus not quite relevant to our histomorphological context, whereas shallow layer features are perhaps too basic and thus not very informative. In Figure S2, another DCNN-based HF extractor (block8_3_ac layer of Inception Resnet V2) recommended in S5 was used to extract HF spectrum and perform tissue segmentation. Different choices appeared to have negligible influence on final segmentation results.

Clustering Analysis Methods

The spectral clustering algorithm was used to segment the tissue section into a predefined number of regions that either have similar mass profiles or histomorphological appearances. Spectral clustering is a graph theory-inspired technique, where clustering is reformulated as grouping nodes in a graph based on the edges connecting them. By cutting the graph such that the weights of the edges between different groups are low and the weights of the edges within the same group are high, data points in different clusters became dissimilar from each other, and points within a cluster are similar to each other. The clustering analysis was implemented by the Scikit-learn library. The metric to construct the affinity matrix was "cosine similarity" (with one exception that it was "nearest neighbors" for the HF data of the tumor sample), the way to assign labels was 'kmeans', and the number of eigenvector components was different for different datasets (parameters were empirically configured to produce visually less fragmented regions).

UMAP, Pearson Correlation and Davies–Bouldin Index

Uniform Manifold Approximation and Projection (UMAP) was used to project the high dimensional mass spectra and histomorphological feature spectra datasets into two 3D spaces for visualization. UMAP is a state-of-art manifold learning algorithm based on ideas from algebraic topology and Riemannian geometry.[S6] Compared with t-SNE, UMAP has increased

speed (about one magnitude faster) and better preservation of the global structure. There are two important hyperparameters for UMAP: 'n_neighbors', which is the number of approximate nearest neighbors used to construct an initial high-dimensional graph, was set to 10; 'min_dist', which is the minimum distance between points in a low-dimensional projection, was set to 0.0. We implemented UMAP using the package provided in https://umap-learn.readthedocs.io/ and employed "cosine metric" to measure the similarities between spectra as recommended in S7. Coloring MSI pixels according to their coordinates in the 3D UMAP space produced a tissue map in which similar colors represent pixels with similar mass spectra. Analogously, the segmentation image described the clusters to which pixels belong. The assumption behind the PC approach to determine #Clusters was that with the optimal #Clusters, UMAP and segmentation images should be similar. After a low pass filter (filter of size 3×3) to reduce pixilation, edges were detected in these images using a Canny edge detector (implemented by the OpenCV package). The vector forms of the edge representations of the two images were compared by calculating the PC. The #Clusters with the highest correlation was selected. The assumption behind Davies–Bouldin index is that better clustering produces clusters that are farther apart and less dispersed. DBI was implemented by the Scikit Learn package.

Renal Tumor Sample

The MSI data set of the renal tumor sample has been previously used in our previous work[S8] for different research purposes.

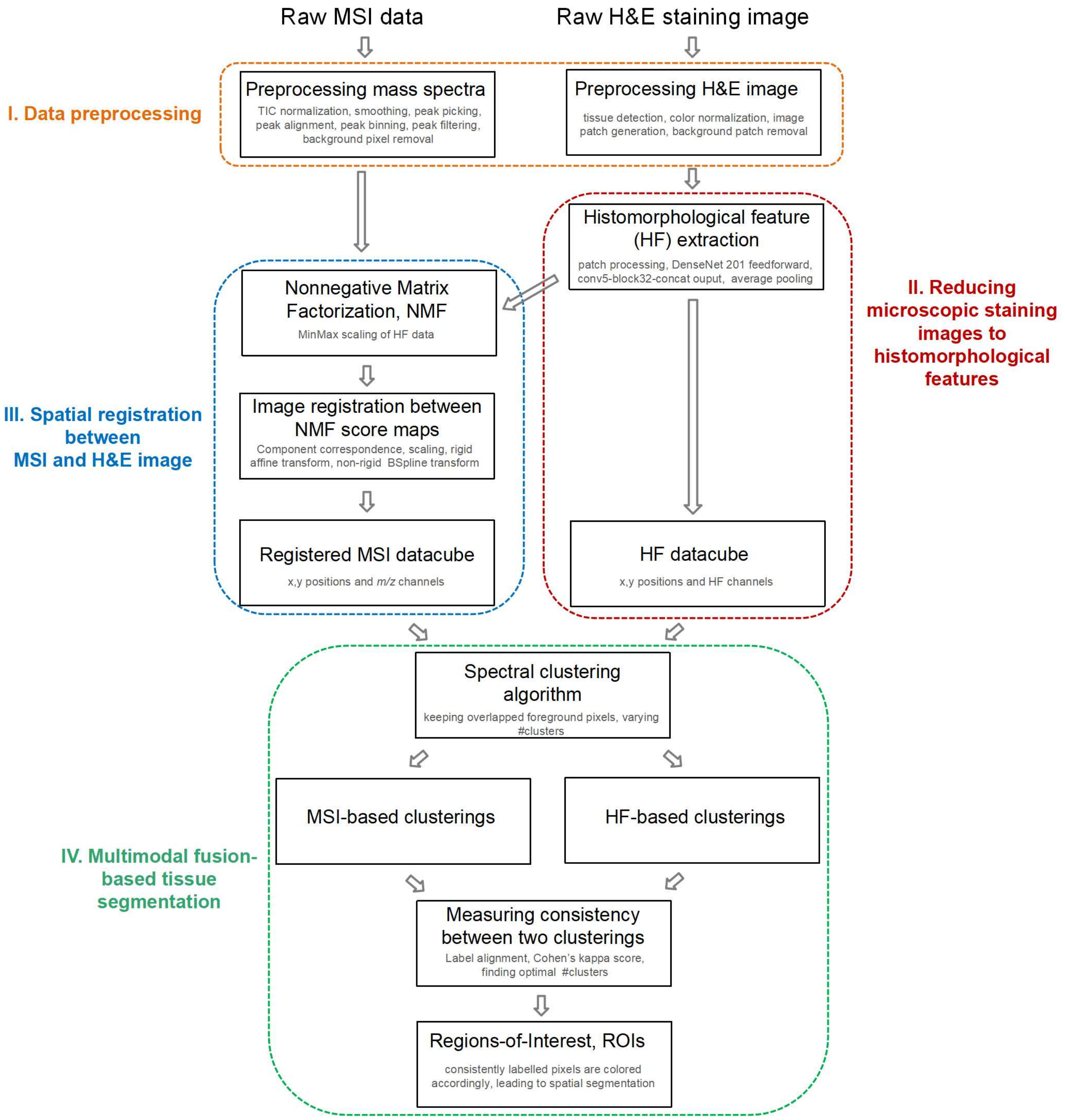
Raw MSI data
Raw H&E staining image
I. Data preprocessing
Preprocessing mass spectra
TIC normalization, smoothing, peak picking, peak alignment, peak binning, peak filtering, background pixel removal
Preprocessing H&E image
tissue detection, color normalization, image patch generation, background patch removal
Histomorphological feature (HF) extraction
patch processing, DenseNet 201 feedforward, conv5-block32-concat ouput, average pooling
II. Reducing microscopic staining images to histomorphological features
Nonnegative Matrix Factorization, NMF
MinMax scaling of HF data
III. Spatial registration between MSI and H&E image
Image registration between NMF score maps
Component correspondence, scaling, rigid affine transform, non-rigid BSpline transform
Registered MSI datacube
x,y positions and m/z channels
HF datacube
x,y positions and HF channels
Spectral clustering algorithm
keeping overlapped foreground pixels, varying #clusters
MSI-based clusterings
HF-based clusterings
IV. Multimodal fusion-based tissue segmentation
Measuring consistency between two clusterings
Label alignment, Cohen's kappa score, finding optimal #clusters
Regions-of-Interest, ROIs
consistently labelled pixels are colored accordingly, leading to spatial segmentation

Figure S1: The workflow of multimodal fusion-based ROI delineation. **(I)** Data preprocessing: raw MSI data were preprocessed by standard protocols for mass spectra, including TIC normalization, smoothing, peak picking, peak alignment, peak binning, peak filtering. A pixel was removed as background if its sum of peak intensities of tissue-specific ions was below a threshold value. Raw H&E image underwent tissue detection and colour normalization before being split into an array of small tiles with a physical size equal to that of a MSI pixel. A tile was removed as background if its percentage of tissue area was less than 90%. **(II)** Encoding H&E image tiles by histomorphological features (HF): H&E tiles were processed and propagated through a DCNN (DenseNet 201)-based HF extractor. The outputs of a middle layer (conv5-block32-concat) of DenseNet 201 were reduced to HF spectra by global average pooling. HF spectra were formatted as a hyperspectral data cube with x,y positions and HF channels. **(III)** Spatial registration between MSI and H&E image: Nonnegative Matrix Factorization (NMF) was used to reduce the dimensions of the MSI and HF data to a number of components. HF data had to be processed by feature-wise Min-Max scaling because NMF required positive data values. Representative NMF components were manually chosen for the MSI and HF data by finding the pair of components whose score maps visually matched each other. After being scaled to a similar intensity range, the representative score map of MSI data was spatially aligned to that of HF data using a combination of linear and non-linear automatic registration algorithms (based on affine and bspline transforms, respectively). We applied the spatial transforms obtained above to every m/z channel of the MSI datacube and produced a registered data cube. **(IV)** Multimodal fusion-based tissue segmentation: spectral clustering algorithm was used to cluster the foreground pixels of the MSI and HF datacubes respectively. Crossmodal consistencies between MSI- and HF-based clusterings were measured for varying #Clusters using Cohen's kappa score (CKS) after labeling alignment. We chose the #Clusters that produced the highest CKS and used consistently labelled pixels as the final regions-of-interest (ROI).

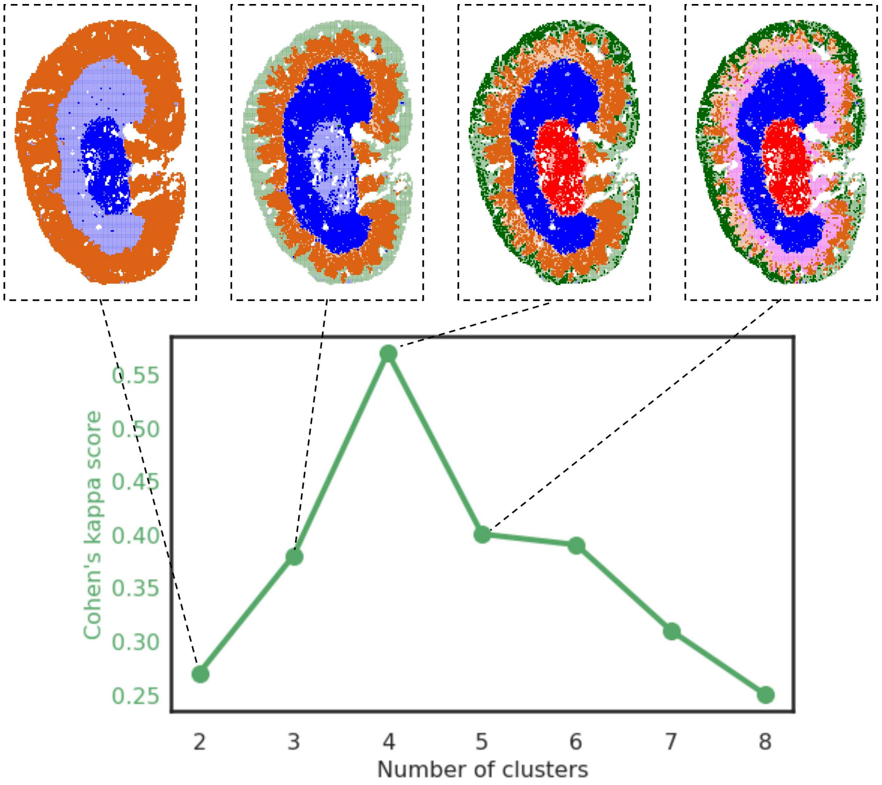

Figure S2: CKS dependence on #Clusters using HF extracted by the block8_3_ac layer of Inception Resnet V2 and integrated segmentation maps at #Clusters = 2 to 5. It appeared that ROI results generated using the Inception Resnet were very similar to the DenseNet and the rank of cross-modal clustering/segmentation consistencies remained almost the same, suggesting that the specific choice of DCNN extractor had negligible influence on the outcome of our strategy.

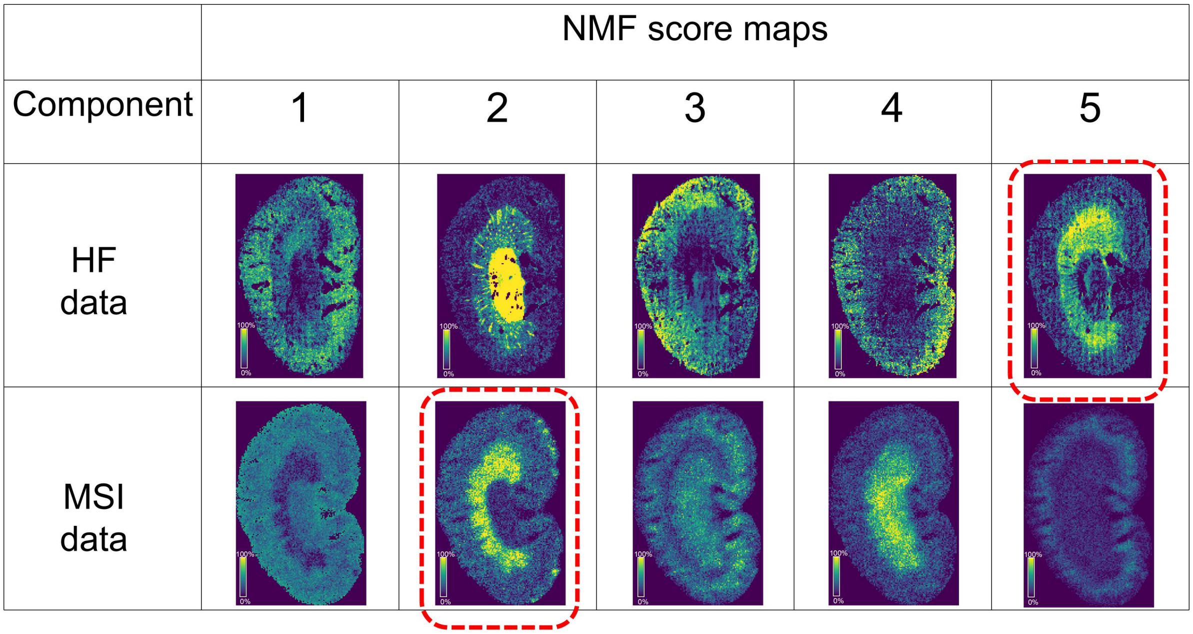

Figure S3: The score maps of different components obtained by decomposing the HF and MSI data of the kidney sample with NMF. The score maps of the 5th and 2nd components (red dashed box) of the HF and MSI data were used as the fixed and moving images for the following registration algorithms.

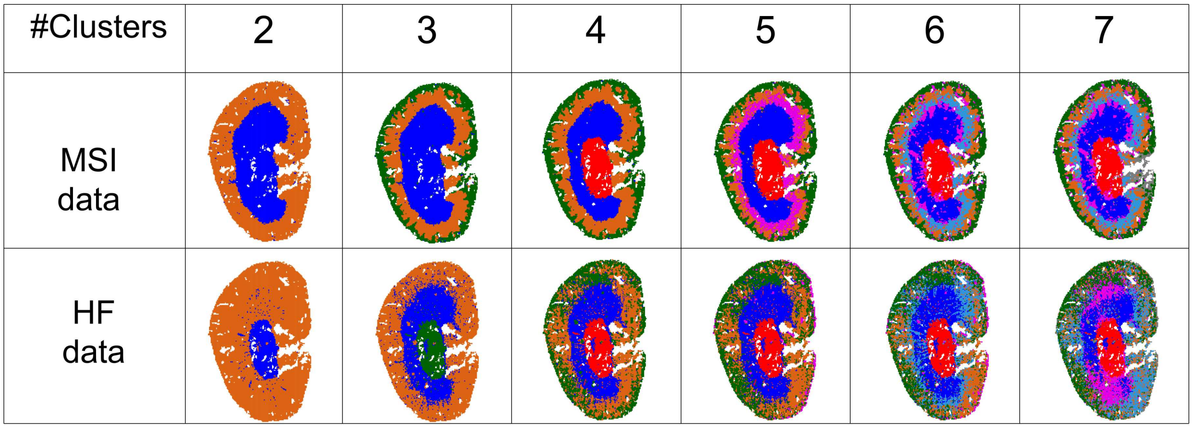

Figure S4: The segmentation maps of HF and MSI data with different #Clusters.

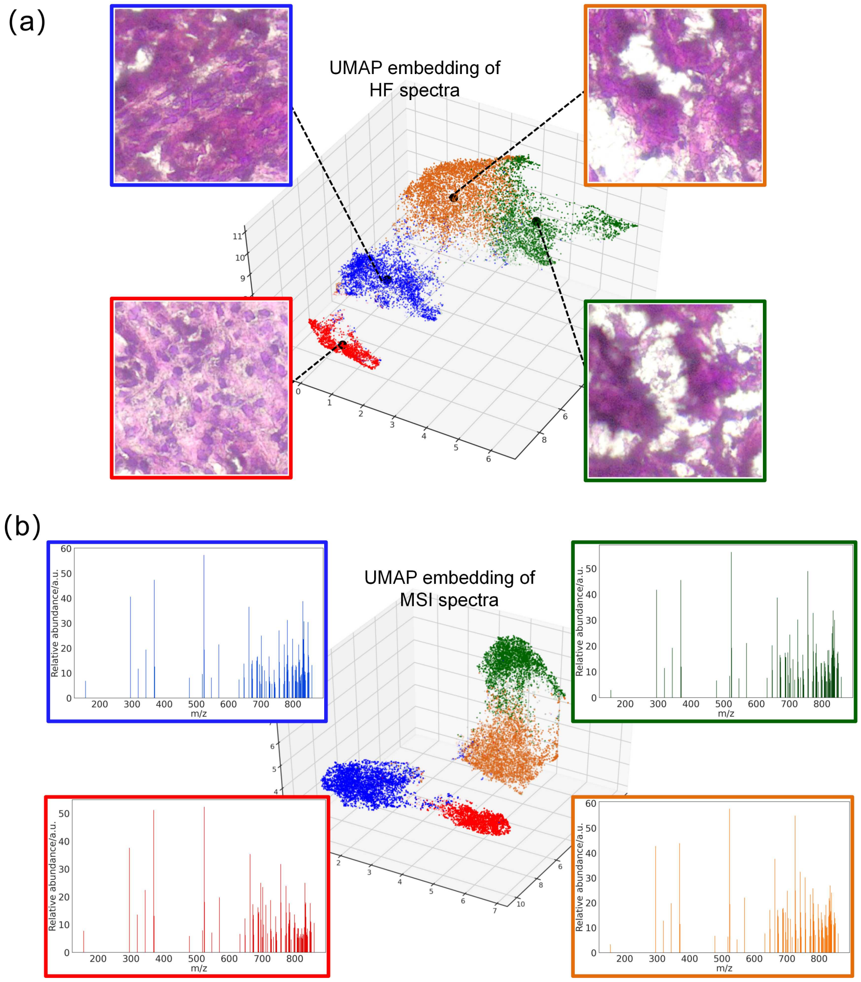

Figure S5: Visualizing the clusterings of the HF spectra and mass spectra of the kidney sample. (a) the high dimensional HF spectra were embedded in a 3D space using the nonlinear dimension reduction method UMAP and colored according to the clusters they belonged to; four characteristic H&E image tiles are displayed, which correspond to the data points at the center of each cluster in the 3D UMAP space. (b) 3D scatter plot of the UMAP embedding of the mass spectra. The mean spectra of each cluster are displayed.

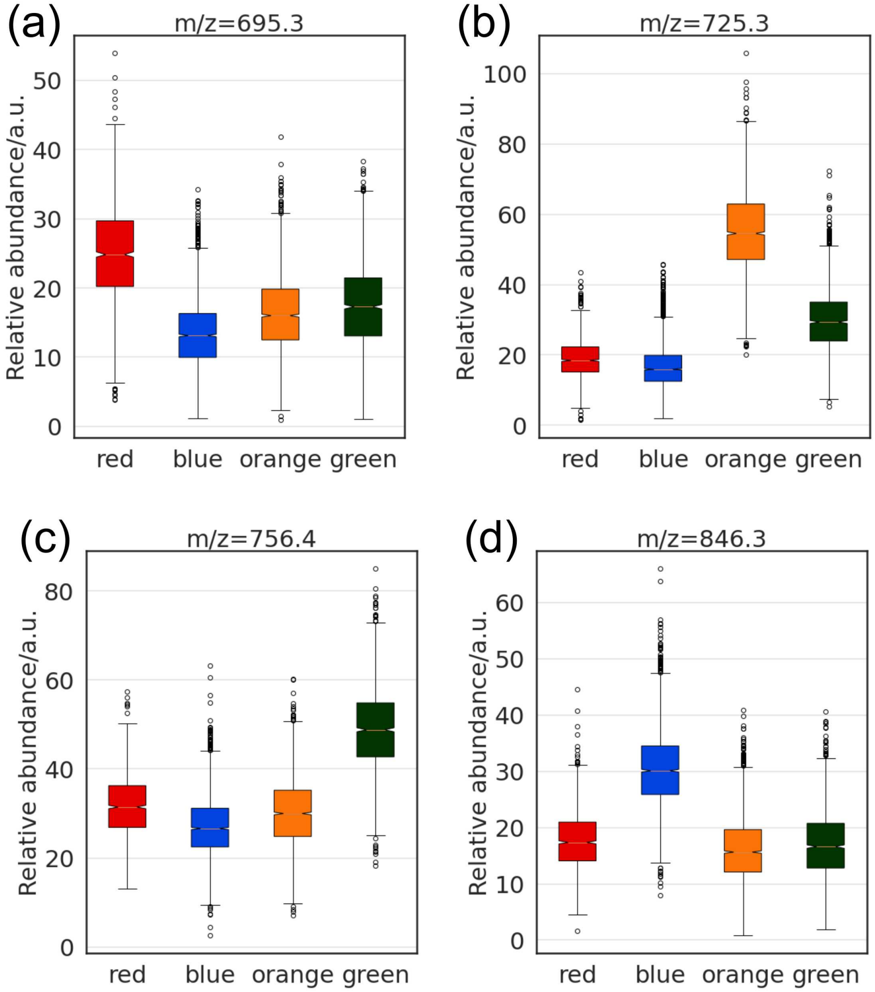

Figure S6: Characteristic m/z variables associated with different kidney regions. (a)-(d) box-plots for the ion abundances of 695.3m/z, 725.3 m/z, 756.4 m/z and 846.3 m/z, which co-localized with the red, orange, green, and blue ROIs respectively.

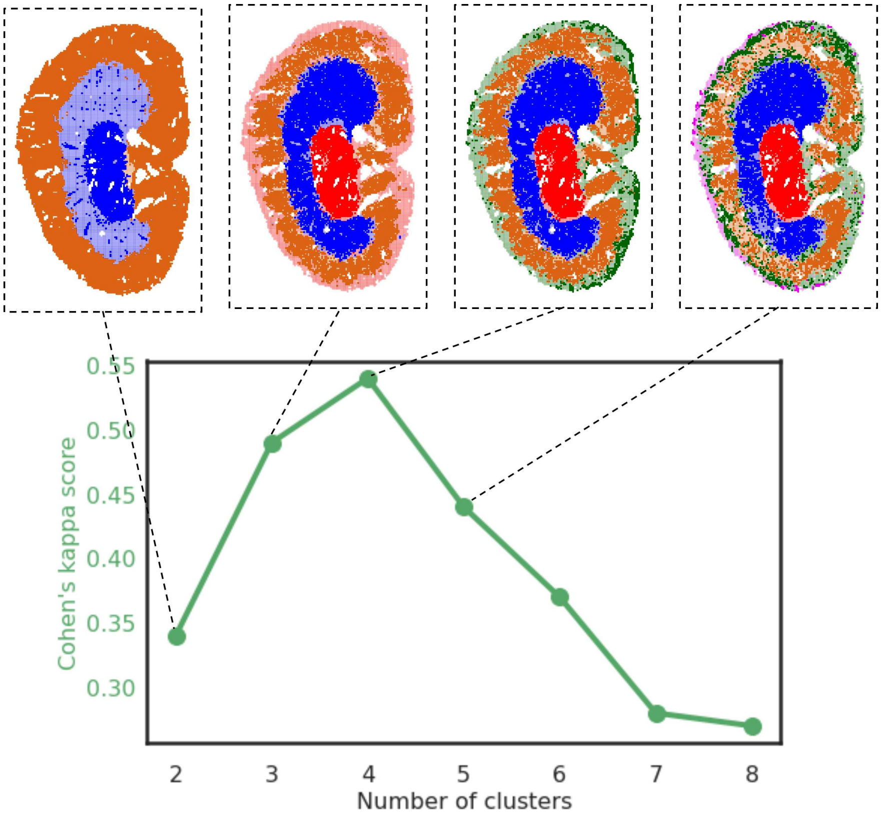

Figure S7: Repeating our ROI delineation method on another adjacent mouse kidney section. CKS dependence on #Clusters suggested that the optimal #Clusters was 4, which was in line with the result described in Figure 3.

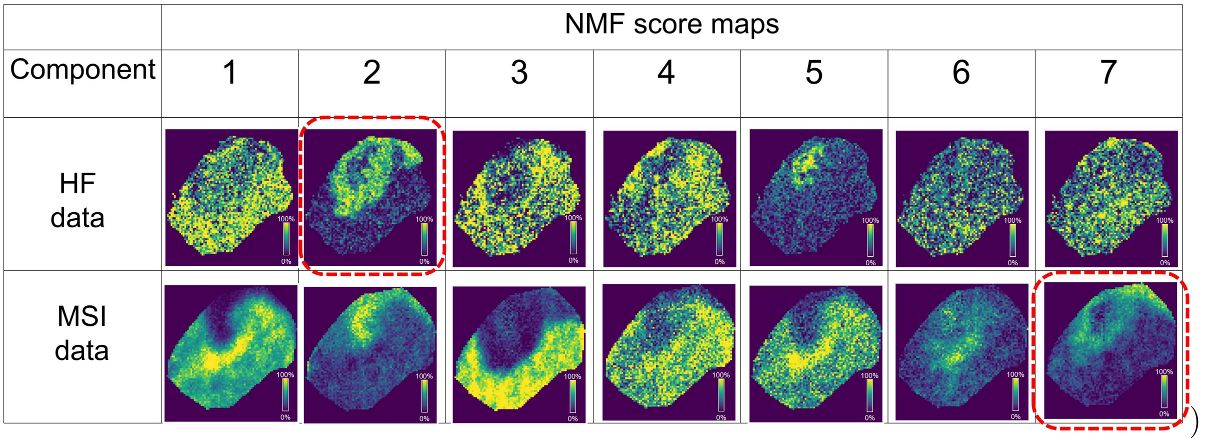

Figure S8: The score maps of different components obtained by decomposing the HF and MSI data of the tumor sample with NMF. The score maps of the 2nd and 7th components (red dashed box) of the HF and MSI data were used as the fixed and moving images for the following registration algorithms.

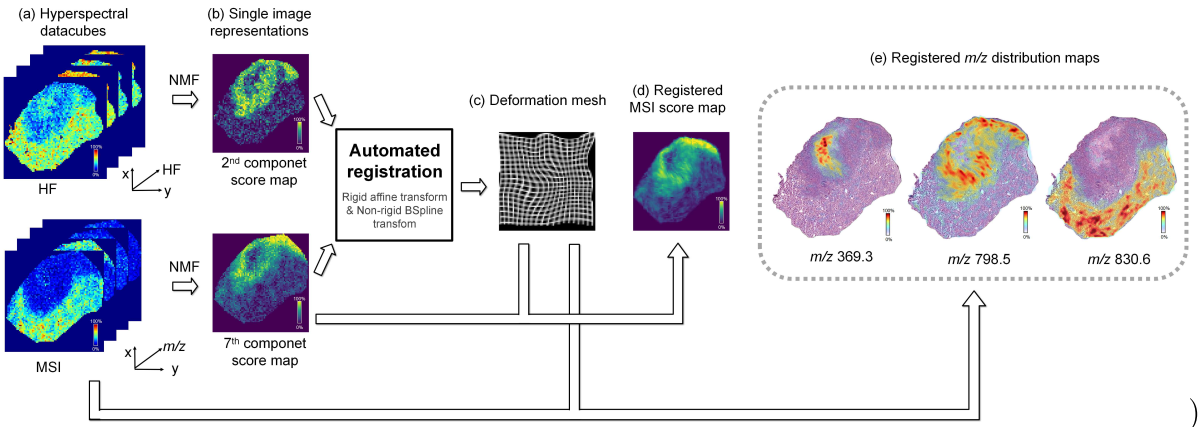

)

Figure S9: Multimodal registration between the HF and MSI data of the renal tumor sample. (a) the hyperspectral datacubes of HF and MSI, (b) the score maps of the 2nd and 7th NMF components of the HF and MSI data, which were seen as their respective single image representations and used as inputs to automated registration algorithms, (c) deformation mesh to visualize the spatial transform output by registration algorithms, (d) the MSI score map was aligned to the HF datacube by the above transform, (e) ion maps of all m/z were registered, upsampled, and overlapped on top of the H&E image.

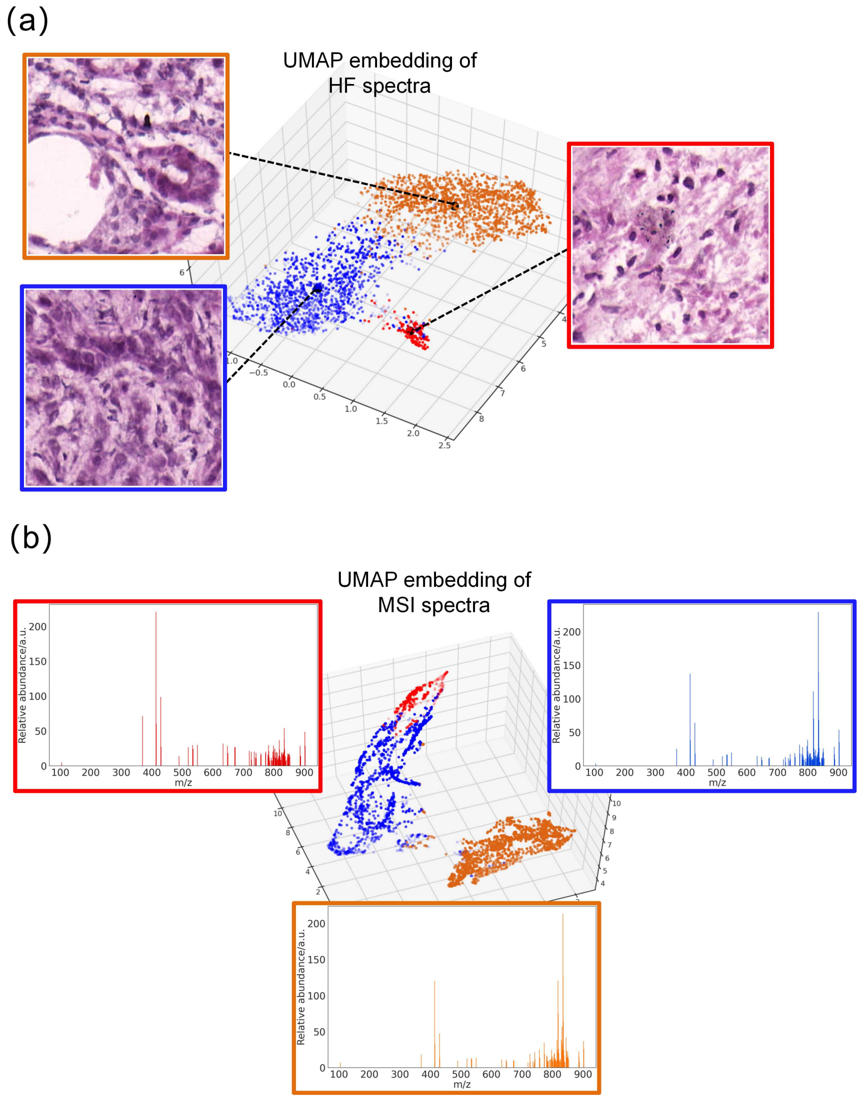

Figure S10: Visualizing the clusterings of the HF spectra and mass spectra of the tumor sample. (a) the high dimensional HF spectra were embedded in a 3D space using nonlinear dimension reduction method UMAP and coloured according to the clusters they belonged to; three characteristic H&E image tiles are displayed, which correspond to the data points at the center of each cluster in the 3D UMAP space. (b) 3D scatter plot of the UMAP embedding of the mass spectra. The mean spectra of each cluster are displayed.

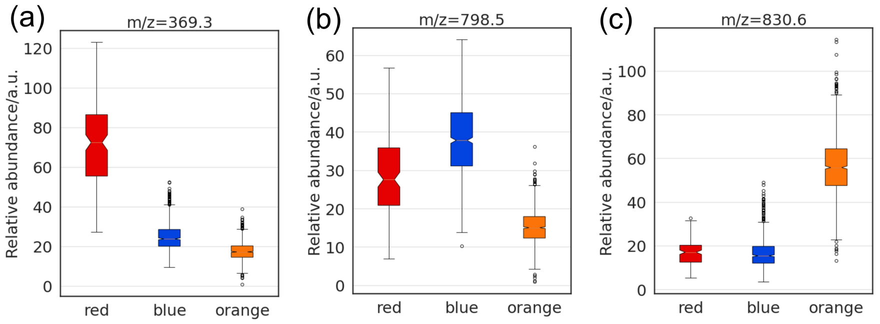

Figure S11: Characteristic m/z variables associated with different tumor regions. (a)-(c) box-plots for the ion abundances of 369.3 m/z, 798.5 m/z, and 830.6 m/z, which co-localized with red, blue, and orange ROIs respectively.

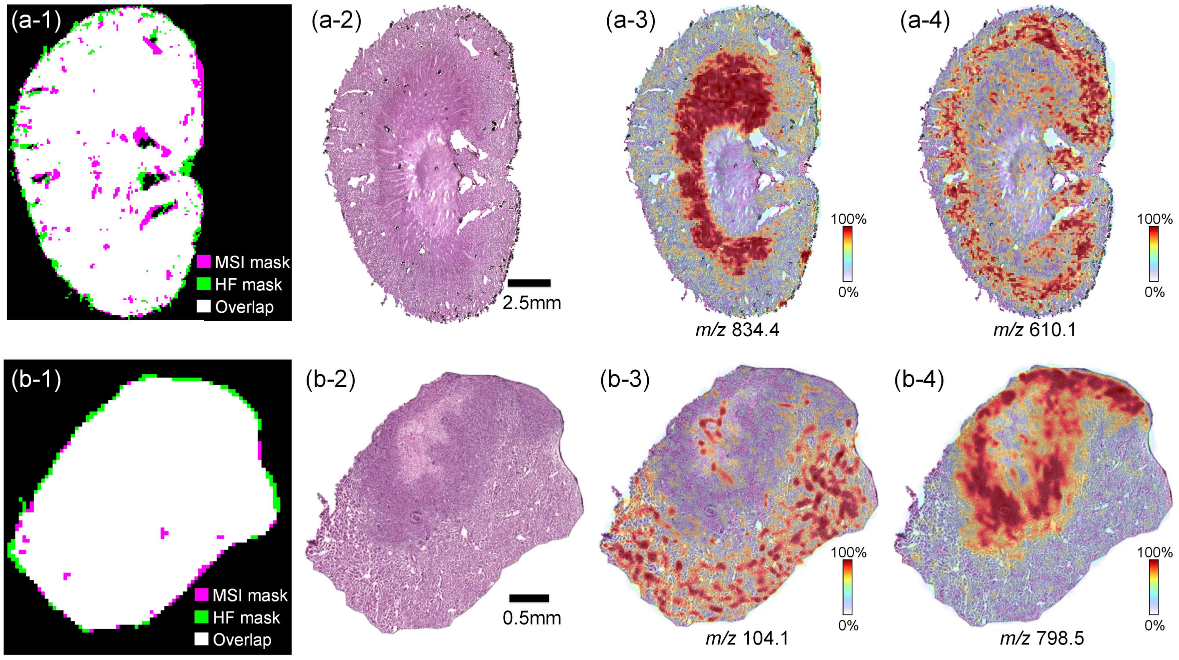

Figure S12: The evaluation of registration quality. (a-1) Good overlap between the HF-derived and MSI-derived kidney tissue masks after registration. (a-2) to (a-4) There is good spatial alignment between the distinct anatomical features observable in both the H&E and MSI images of the kidney tissue. (b-1) to (b-4) the same for the tumor tissue sample.

References

(S1) Reinhard, E.; Adhikhmin, M.; Gooch, B.; Shirley, P. Color transfer between images. *IEEE Computer Graphics and Applications* **2001**, *21*, 34–41.

(S2) Cooper, L. HistomicsTK. 2016; `https://github.com/DigitalSlideArchive/HistomicsTK`.

(S3) LeCun, Y.; Bengio, Y.; Hinton, G. Deep learning. *nature* **2015**, *521*, 436–444.

(S4) Zeiler, M. D.; Fergus, R. Visualizing and understanding convolutional networks. European conference on computer vision. 2014; pp 818–833.

(S5) Mormont, R.; Geurts, P.; Maree, R. Comparison of Deep Transfer Learning Strategies for Digital Pathology. 2018 IEEE/CVF Conference on Computer Vision and Pattern Recognition Workshops (CVPRW). 2018.

(S6) McInnes, L.; Healy, J.; Melville, J. Umap: Uniform manifold approximation and projection for dimension reduction. *arXiv preprint arXiv:1802.03426* **2018**,

(S7) Smets, T.; Verbeeck, N.; Claesen, M.; Asperger, A.; Griffioen, G.; Tousseyn, T.; Waelput, W.; Waelkens, E.; De Moor, B. Evaluation of distance metrics and spatial autocorrelation in uniform manifold approximation and projection applied to mass spectrometry imaging data. *Analytical chemistry* **2019**, *91*, 5706–5714.

(S8) Ang, G.; Zhiyu, C.; Yinzhong, M.; Yueguang, L.; Huanhuan, Y.; Qichan, G.; Xing, Y.; Fang, L.; Qian, L.; Hairong, Z. Multimodal Coregistration and Fusion between Spatial Metabolomics and Biomedical Imaging. *In submission* **2022**,
