# Supplementary material for: Delineating regions of interest for mass spectrometry imaging by multimodally corroborated spatial segmentation
Source: Gigascience. 2023 Apr 11;12:giad021. doi: 10.1093/gigascience/giad021 (PMC10087011; doi:10.1093/gigascience/giad021)
Supplement: giad021_Response_to_Reviewer_Comments_Original_Submission [file giad021_response_to_reviewer_comments_original_submission.pdf]

Author's Response To Reviewer Comments

Close

///In addition, please register any new software application in the bio.tools and SciCrunch.org databases to receive RRID (Research Resource Identification Initiative ID) and biotoolsID identifiers, and include these in your manuscript. Computational workflows should be registered in workflowhub.eu and the DOIs cited in the relevant places in the manuscript. These will facilitate tracking, reproducibility and re-use of your tool.///

Response:
RRID, biotoolsID identifiers, and workflowhub.eu DOI added (revised manuscript, lines 538-541).

///Please include a point-by-point within the 'Response to Reviewers' box in the submission system. Please ensure you describe additional experiments that were carried out and include a detailed rebuttal of any criticisms or requested revisions that you disagreed with. Please also ensure that your revised manuscript conforms to the journal style, which can be found in the Instructions for Authors on the journal homepage.

If the data and code has been modified in the revision process please be sure to update the public versions of this too.///

Response:
The public versions of the data and code have been updated.

///Reviewer reports:
Reviewer #1: In this interesting Research article, a Deep Learning-based algorithm is used to extract histomorphological DESI mass spectrometry imaging (MSI) feature spectra from H&E images. A key aspect of this approach is the use of a deep convolutional neural network (DCNN)-based feature extractor to generate spectra representative of microanatomical histomorphology in MSI pixels. Clustering is then used for spatial segmentation of regions of interest. As use case scenarios, the authors explored mouse kidney, and a mouse model of orthotopic human renal adenocarcinoma. Lipid dysregulation is a known pathological correlate in human renal adenocarcinoma, and the use of DESI mass spectrometry imaging enables spatial lipidomic analysis to be accomplished on H&E sections. The deep convolutional neural network (DCNN) approach utilised in this Research article is an interesting alternative to manual annotation, and the authors are to be commended for exploring automated alternatives to labour-intensive manual annotation.

Source code from this work are freely accessible from GitHub (https://github.com/guoang4github/ROIforMSI/) where they have been ascribed an Open Source Initiative-approved GPL-3.0 Licence.

The MSI data include raw and processed MSI data in imzML format, and are available from the ProteomeXchange with accession identifier PXD03887. The TIF image files of mouse kidney and orthotopic human renal adenocarcinoma are additionally included in PXD03887.

Source code, MSI data, and H&E stained histological images used in this analysis have also been uploaded to GigaDB.

Major comment
In support of this manuscript, I will also require the following:
Regions of Interest (ROIs) generated by spatial segmentation, for example as binary masks associated with the H&E stained histological images. This invaluable output file would allow a researcher to overlay the ROIs, generated by spatial clustering, over the original H&E images. This is especially useful, and it will additionally allow researchers to assess pixelation in ROIs.///

Response:
We appreciate the reviewer's supportive comments, which are very helpful in improving the manuscript. Following the reviewer's suggestion, the ExampleWorkflow.ipyb files of our shared source code (both the files on the github and ftp servers) have been modified: the functionalities to output the ROI/label map generated by our strategy and to overlay it on the H&E image have been added. The ROI maps are stored as a 2D matrix where each element (i.e. pixel) records an integer corresponding to its assigned tissue category. The ROI maps of the kidney and tumour samples are uploaded to the FTP server ("kidneyMapOfROIs.npy" and "tumorMapOfROIs.npy").

///In addition, I have been informed that the mass spectrometry annotations are available from the DOME-ML Registry (https://registry.dome-ml.org/) with the following identifier:
d8887555-ed67-474f-a85d-798712d9ffa9
I cannot find this annotation on DOME-ML. Can you please send a link to the DOME-ML annotation?///

Response:
According to the DOME-ML Registry policy ("Submitted annotations will go through a manual revision process and will eventually be published. Published annotations will be made publicly available and will no longer be editable. Instead, unpublished annotations can only be viewed, edited or deleted by you."), our submission is probably still in the review process. I've tried to contact them to speed up the process, but haven't heard back yet. So I've uploaded a document ("DOME-Registry-Annotations.docx") of the annotations to the FTP server.

///Reviewer #2: The authors have presented in the current manuscript alternative data analysis tools which will benefit the mass spectrometry imaging community. Their focus was on determining the optimal number of regions of biological interest within tissues by multimodal registration and clustering. The authors underline the importance of unbiased segmentation of spectral data which also fits with biologically significant results.

The work carried out in the presented manuscript is highly useful within the context of mass spectrometry imaging data analysis. However, recent (MALDI) MS imaging datasets are nowadays collected with 20, 10 or even 5 um lateral resolutions, resulting in significantly increased number of pixels per tissue, and processing time, respectively. The authors claim that their tool can be used on any type of MSI data, independent of instrument or ionization techniques. This statement would be more convincing if the authors considered adding an example for their tool on one such dataset, or if the authors mentioned what is the required data format for using their tool (I assume imzML?). ///

Response:
We appreciate the reviewer's supportive comments and suggestions, which we found very helpful. We acknowledge that we may have been overly optimistic about the generic nature of our strategy. So we toned down the statement and the required data format (imzML) has been mentioned.

Please see below reworded sentences.
"Our strategy is in principle applicable to other MSI ionization technologies such as Secondary Ion Mass Spectroscopy (SIMS) or MALDI (note that the software tool we provide is coded in Python, so the data format output by MSI instruments must be Python compatible, e.g. imzML), but follow-up studies are required to test its genericity." (revised manuscript lines 373-378)

///Additionally, the authors should consider testing their tool on data collected with higher lateral resolution for MSI (e.g 10 um), because it would increase the possibility of associating "unconfident pixels" which appeared "at the boundaries between ROIs" to either existing clusters, or to generate new clusters. The resolution disparity between imaging modalities is too high in my opinion; H&E images enable distinguishing between cell types, but this information is lost because the image is down-sized to tiles which match the size of the MSI raster, thus containing information from several cells (but with no spatial differentiation) or even from morphological structures as glomeruli (~50um). Similarly, in the example of the tumor tissue, the inconclusive pixels, represented by transparent clusters in Figure 4 (b-4), might describe specific tissue types between healthy and cancer, and necrotic and tumor, respectively. I consider

this a limitation that should be addressed by the authors.///

Response:
We agree with the reviewer that testing our method on higher resolution MSI data would strongly support the versatility of our strategy and may further improve the accuracy and granularity of the spatial segmentation results. However, there are three reasons why we cannot do this:
1.We currently don't have access to an MSI instrument that can achieve the lateral resolution of 10 um. The physical resolution limit of our common commercial DESI MSI instrument is about 35-50 um due to the size of the plume impact region (Analytical and bioanalytical chemistry, 2012, 404: 389-398.).
2.We've searched the common MSI data repositories (ProteomeXchange and PRIDE) and tried to find publicly available datasets with a spatial resolution of at least 10 um. However, it turned out that none of them included a high-resolution (>20X) H&E microscopy image, which is essential for the strategy proposed in our manuscript.
3.We anticipate that if we want to apply our method to MSI datasets with a resolution of 10 um, we would need a more powerful spatial registration approach capable of dealing with such high spatial resolution. Otherwise, the mismatch between the MSI pixel and the H&E tile due to misalignment could reduce the cross-modal consistency of the spatial segmentation, making our method less reliable. Spatial registration at the 10um (single cell) scale is still an active research area in the MSI community (Nature methods 2021, 18, 7 799. Analytical chemistry 2018, 90, 21 12395. Analytical chemistry 2018, 90, 21 12404) and is beyond the scope of this manuscript.

Of all the MSI datasets in the ProteomeXchange repository, more than 70% (17 out of 23) were acquired with a lateral resolution of worse than 50 um. The majority of current MSI studies are therefore dealing with data with a lateral resolution of worse than 50 um. In other words, even in the worst case that our method is not applicable to high lateral resolution MSI data, it is still useful for the majority of MSI studies, justifying its broad interest to the MSI community. Nevertheless, the reviewer's comment is very insightful and should certainly be taken into account in follow-up studies.

Please see below added sentences.
"In particular, MALDI and SIMS can achieve lateral resolutions greater than 10 um, in which case we anticipate that a more powerful spatial registration approach compatible with the single cell scale will be required to establish accurate correspondence between MSI pixel and H&E tile. Combining our strategy with high spatial resolution MSI data may further improve the granularity and accuracy of the spatial segmentation results. For example, the "unconfident pixels" at the borders between ROIs may be subdivided and "deconvolved", resulting in their assignment to existing clusters or the creation of new clusters."(revised manuscript lines 378-387)

///Data presented in the manuscript are clearly described and the authors provide sufficient context for the reader to understand their potential uses. The authors also mention previous work based on the showcased data. I highly appreciate that the authors offer their tool freely, and that they provide the necessary information for all the data pre-processing as well as offer the readers example data for testing. For their tool to be widely used and appreciated by the community, I would suggest creating a user friendly interface for their software tool.///

Response:
Our group is currently working on a project to develop a more general software with non-programmer-friendly interfaces for multimodal data fusion of MSI, and the strategy proposed in this manuscript will be integrated into this tool.

///The authors have presented appropriate references to the literature, but they have omitted an important publication in the field [https://doi.org/10.1038/s41592-021-01198-0], which uses a similar concept for multimodal registration. ///

Response:
We thank the reviewer for the information on the paper, which is indeed very relevant to our study. A brief description of the paper has been added to the Introduction section.

Please see below the added sentence.
“For example, by integrating MALDI-MSI and microscopy, Rappez et al.[26] have developed a SpaceM method to characterize in situ single cell metabolomics.” (revised manuscript, lines 77-80).

///The methods section was well rounded, but one missing element was the information, tool, database or library they used for annotating several m/z values: e.g. lines 275-278, and lines 307-309.///

Response:
In original manuscript, lines 275-278 and lines 310-311 we selected 7 ions and tentatively identified them according to their mass-to-charge ratio (m/z). Searches of general metabolite/lipid databases, such as lipidmap, returned several possible matches. Unfortunately, without additional analytical information such as MS/MS, we couldn't determine which one was correct. To overcome this problem, we found a more specialised metabolite annotation database (Analytical Chemistry, 2022, 94(20): 7286-7294.) and used it as a reference. The database was built for DESI-MSI using mouse models in an organ-specific way. So, once we had chosen a suitable organ (kidney in our case), we could simply find the identified molecules in the database that might produce ions with an m/z comparable to the ions we had selected. We are now more confident about the identification results because the specialized database uses a similar ion source (i.e. DESI), the same animal models (i.e. mice), and the same organ (i.e. kidney), and it very often returns only a single match for an input m/z. The identification results (revised manuscript, lines 278-281 and lines 313-314) have been revised accordingly. A description of the database is added in the revised manuscript, line 282:
“...according to an organ-specific metabolite database built by Zhu et al [34].”.

///The authors have strengthened their tool by using an orthogonal imaging method for reducing instrumental noise or acquisition artifacts, which I found very valuable as many research teams in the field already use staining protocols in their studies. The information obtained from stained tissues (H&E, IHC) is highly valuable for targeted studies, but it is limited for untargeted ones. Have the authors considered testing their tool on vibrational spectroscopy imaging data like FTIR or Raman? In this case the imaging can be done on the same section with increased ease for multimodal registration and minimal deformation between imaging modalities. Also, these techniques are providing additional, and orthogonal, molecular signatures.///

Response:
As the reviewer points out, other imaging modalities can be used instead of optical microscopy. As an alternative method to label-freely achieve the distribution of molecular species within a sample, vibrational spectroscopic imaging techniques provide molecular information (signals due to vibrations from chemical bonds) complementary to MSI, making them great candidates for multimodal corroboration of spatial segmentation results. In principle, we can partition the tissue by clustering the FTIR or Raman spectra collected at each pixel and compare the spatial segmentation results obtained by vibrational spectroscopic imaging and MSI, respectively. The optical number of clusters can then be determined, just as we have done with the histomorphological feature spectra. But again, we do not currently have access to FTIR or Raman imaging instruments and cannot find suitable publicly available datasets. So we cannot test our method on the said datasets. Nevertheless, this comment is very instructive and we include it as a possible future direction in the conclusion section.

Please see below added sentences.
“Finally, in our multimodal fusion strategy, other imaging modalities can be used instead of optical microscopy. As an alternative method to obtain a label-free distribution of molecular species within a sample, vibrational spectroscopic imaging techniques, such as Fourier Transform Infrared (FTIR) and Raman imaging, capture information about chemical bonds, making them complementary to MSI and great candidates for multimodal corroboration of spatial segmentation. Specifically, we can replace the HF spectra extracted from each tile with corresponding FTIR or Raman spectra, and the rest of our strategy remains essentially the same.” (revised manuscript, lines 388-397)

///The writing is clear, easy to follow, with minimal editing required: e.g. lines 182 "section and SI", and line 354 "more in section": there is a missing pointer to the section in each case.///

Response:
Pointers have been added.

///The data are presented in an appropriate manner, figures are representative of the described results, and almost ready to be published: Figure 2 should be prepared with the same font size as the other figures, and Figure 4 has a closed parenthesis ")" in the bottom right corner which should be removed.///

Response:
The font size has been enlarged and closed parenthesis has been removed.

///Some considerations:
I am missing "clean" optical or H&E images before multimodal registration (without the applied deformation mesh) to assess the quality of the tissue sections and the multimodal registration result. ///

Response:
H&E images have been added to Figure S12.

///Figure 5 (b) contains the only representation of the two tissue sections, where the MSI NMF score map seems to showcase a more intact tissue section, compared to the H&E score map, where the holes and tears in the tissue are visible. Could these missing pixels from the MSI data after registration omit some valuable information from the tissue? Figure S4 illustrates that the regions of the holes and tears are present in the MSI imaging data only after multimodal registration. In this context, how do you validate the registration of the two images?///

Response:
The original Figure 3(h-i) and 5(b) may be misleading. In fact, there are holes in the MSI tissue section (as shown in the tissue mask in Figure S12). The holes are not expected to be exactly the same, as serial tissue sections may be slightly different. When performing the spatial segmentation, only pixels/tiles marked as "tissue" by both the MSI and HF masks are used for the subsequent clustering analysis. In other words, the final tissue mask is the Hadamard product of the two binary masks. A large proportion of the holes correspond to the veins present in both tissue sections. The MSI signal in these areas therefore comes from the background glass slide and is therefore not informative. Indeed, there are MSI 'tissue' pixels that are mistakenly tagged as 'background' by the final mask, and the potentially valuable information they carry is omitted. However, compared to the total tissue, the proportion of mislabelled pixels is relatively small, so we don't expect that the information they carry would significantly change the results of the spatial segmentation and its evaluation based on cross-modal consistency.

Figure 3 (h-i) and 5 (b) are changed: the MSI tissue mask is applied to the ion maps.

Figure S12 is added to demonstrate the validation of the registration. The registration quality is visually inspected by the two approaches: (1) the overlap between the HF-derived and MSI-derived tissue masks after registration; (2) the spatial alignment between the distinguishable anatomical features observed in the H\&E and MSI images.

Please see below an added sentence.
“Figure.S12 shows a visual assessment of the registration quality: good overlap between the HF-derived and MSI-derived tissue masks, and between the distinct anatomical features observed in both HF and MSI images, indicate good registration results.” (revised manuscript, lines 509-513)

///On page 5 lines 312-315, the statement "By comparing the MSI- and histology-segmentation results, we became able to automatically and quantitatively determine to which extent that molecular variation could be explained by underlying histomorphological variation." gives the impression that obtaining the #clusters was also done in a quantitative manner, e.g. by multiple adjacent sections' analysis. Please consider rephrasing.///

Response:

“unbiasedly” is used instead of “quantitatively”. (revised manuscript, lines 317)

///Reviewer #3: GigaScience review

This is a well explained and demonstrated article for which I only have minor suggestions for improvement.///

Response:
We really appreciate the reviewer’s positive comments.

///Introduction comments
Authors state "...thus quantifying molecules in a spatially-resolved manner...". I think "localizing" molecules is a better very than quantifying since quantifying implies absolute quantitation in the field of mass spectrometry and quantifying endogenous proteins and metabolites by imaging MS is not routine. The authors are discussing the analysis of data through mathematical methods.///

Response:
“quantifying” is replaced with “localizing” (revised manuscript, first line in abstract and line 4)

///"homebrew algorithm" -> in-house algorithm///

Response:
"homebrew algorithm" is replaced with “in-house” (revised manuscript, first line in abstract and line 93)

Close
