# Supplementary material for: Delineating regions of interest for mass spectrometry imaging by multimodally corroborated spatial segmentation
Source: Gigascience. 2023 Apr 11;12:giad021. doi: 10.1093/gigascience/giad021 (PMC10087011; doi:10.1093/gigascience/giad021)
Supplement: giad021_Response_to_Reviewer_Comments_Revision_1 [file giad021_response_to_reviewer_comments_revision_1.pdf]

Author's Response To Reviewer Comments

Close

1. DOIs should be cited in the references and only Reference # cited in the main text.

response: DOIs have been moved to the references.

2. Please add the GigaDB DOI citation - I have included exact text on what to add under "Availability of supporting data" and also how to cite the DOI in the references. Please add the Reference number to the Data availability section.

response: GigaDB DOI citation has been added.

Close
