# Supplementary material for: Delineating regions of interest for mass spectrometry imaging by multimodally corroborated spatial segmentation
Source: Gigascience. 2023 Apr 11;12:giad021. doi: 10.1093/gigascience/giad021 (PMC10087011; doi:10.1093/gigascience/giad021)
Supplement: giad021_Reviewer_1_Report_Original_Submission — Chris Armit -- 1/20/2023 Reviewed [file giad021_reviewer_1_report_original_submission.pdf]

Reviewer Report

Title: Delineating Regions-of-interest for Mass Spectrometry Imaging by Multimodally Corroborated Spatial Segmentation

Version: Original Submission **Date:** 1/20/2023

Reviewer name: Chris Armit

Reviewer Comments to Author:

In this interesting Research article, a Deep Learning-based algorithm is used to extract histomorphological DESI mass spectrometry imaging (MSI) feature spectra from H&E images. A key aspect of this approach is the use of a deep convolutional neural network (DCNN)-based feature extractor to generate spectra representative of microanatomical histomorphology in MSI pixels. Clustering is then used for spatial segmentation of regions of interest. As use case scenarios, the authors explored mouse kidney, and a mouse model of orthotopic human renal adenocarcinoma. Lipid dysregulation is a known pathological correlate in human renal adenocarcinoma, and the use of DESI mass spectrometry imaging enables spatial lipidomic analysis to be accomplished on H&E sections. The deep convolutional neural network (DCNN) approach utilised in this Research article is an interesting alternative to manual annotation, and the authors are to be commended for exploring automated alternatives to labour-intensive manual annotation.
Source code from this work are freely accessible from GitHub (https://github.com/guoang4github/ROIforMSI/) where they have been ascribed an Open Source Initiative-approved GPL-3.0 Licence.
The MSI data include raw and processed MSI data in imzML format, and are available from the ProteomeXchange with accession identifier PXD03887. The TIF image files of mouse kidney and orthotopic human renal adenocarcinoma are additionally included in PXD03887.
Source code, MSI data, and H&E stained histological images used in this analysis have also been uploaded to GigaDB.
Major comment
In support of this manuscript, I will also require the following:
Regions of Interest (ROIs) generated by spatial segmentation, for example as binary masks associated with the H&E stained histological images. This invaluable output file would allow a researcher to overlay the ROIs, generated by spatial clustering, over the original H&E images. This is especially useful, and it will additionally allow researchers to assess pixelation in ROIs.
In addition, I have been informed that the mass spectrometry annotations are available from the DOME-ML Registry (https://registry.dome-ml.org/) with the following identifier:
d8887555-ed67-474f-a85d-798712d9ffa9
I cannot find this annotation on DOME-ML. Can you please send a link to the DOME-ML annotation?

Methods

Are the methods appropriate to the aims of the study, are they well described, and are necessary controls included? Choose an item.

Conclusions

Are the conclusions adequately supported by the data shown? Choose an item.

Reporting Standards

Does the manuscript adhere to the journal's guidelines on [minimum standards of reporting?] Choose an item.

Choose an item.

Statistics

Are you able to assess all statistics in the manuscript, including the appropriateness of statistical tests used? Choose an item.

Quality of Written English

Please indicate the quality of language in the manuscript: Choose an item.

Declaration of Competing Interests

Please complete a declaration of competing interests, considering the following questions:

- Have you in the past five years received reimbursements, fees, funding, or salary from an organisation that may in any way gain or lose financially from the publication of this manuscript, either now or in the future?
- Do you hold any stocks or shares in an organisation that may in any way gain or lose financially from the publication of this manuscript, either now or in the future?
- Do you hold or are you currently applying for any patents relating to the content of the manuscript?
- Have you received reimbursements, fees, funding, or salary from an organization that holds or has applied for patents relating to the content of the manuscript?
- Do you have any other financial competing interests?
- Do you have any non-financial competing interests in relation to this paper?

If you can answer no to all of the above, write 'I declare that I have no competing interests' below. If your reply is yes to any, please give details below.

I work for GigaScience where I perform checks on the quality of image data and the image analysis approach. My funding is not dependent on the outcome of this review, and I declare that I have no competing interests.

I agree to the open peer review policy of the journal. I understand that my name will be included on my report to the authors and, if the manuscript is accepted for publication, my named report including any attachments I upload will be posted on the website along with the authors' responses. I agree for my report to be made available under an Open Access Creative Commons CC-BY license (http://creativecommons.org/licenses/by/4.0/). I understand that any comments which I do not wish to be included in my named report can be included as confidential comments to the editors, which will not be published.

Choose an item.

To further support our reviewers, we have joined with Publons, where you can gain additional credit to further highlight your hard work (see: https://publons.com/journal/530/gigascience). On publication of this paper, your review will be automatically added to Publons, you can then choose whether or not to claim your Publons credit. I understand this statement.

Yes Choose an item.
