# Supplementary material for: Delineating regions of interest for mass spectrometry imaging by multimodally corroborated spatial segmentation
Source: Gigascience. 2023 Apr 11;12:giad021. doi: 10.1093/gigascience/giad021 (PMC10087011; doi:10.1093/gigascience/giad021)
Supplement: giad021_Reviewer_2_Report_Original_Submission — Stefania Alexandra Iakab -- 2/5/2023 Reviewed [file giad021_reviewer_2_report_original_submission.pdf]

Reviewer Report

Title: Delineating Regions-of-interest for Mass Spectrometry Imaging by Multimodally Corroborated Spatial Segmentation

Version: Original Submission **Date:** 2/5/2023

Reviewer name: Stefania Alexandra Iakab

Reviewer Comments to Author:

The authors have presented in the current manuscript alternative data analysis tools which will benefit the mass spectrometry imaging community. Their focus was on determining the optimal number of regions of biological interest within tissues by multimodal registration and clustering. The authors underline the importance of unbiased segmentation of spectral data which also fits with biologically significant results.
The work carried out in the presented manuscript is highly useful within the context of mass spectrometry imaging data analysis. However, recent (MALDI) MS imaging datasets are nowadays collected with 20, 10 or even 5 um lateral resolutions, resulting in significantly increased number of pixels per tissue, and processing time, respectively. The authors claim that their tool can be used on any type of MSI data, independent of instrument or ionization techniques. This statement would be more convincing if the authors considered adding an example for their tool on one such dataset, or if the authors mentioned what is the required data format for using their tool (I assume imzML?). Additionally, the authors should consider testing their tool on data collected with higher lateral resolution for MSI (e.g 10 um), because it would increase the possibility of associating "unconfident pixels" which appeared "at the boundaries between ROIs" to either existing clusters, or to generate new clusters. The resolution disparity between imaging modalities is too high in my opinion; H&E images enable distinguishing between cell types, but this information is lost because the image is down-sized to tiles which match the size of the MSI raster, thus containing information from several cells (but with no spatial differentiation) or even from morphological structures as glomeruli (~50um). Similarly, in the example of the tumor tissue, the inconclusive pixels, represented by transparent clusters in Figure 4 (b-4), might describe specific tissue types between healthy and cancer, and necrotic and tumor, respectively. I consider this a limitation that should be addressed by the authors.
Data presented in the manuscript are clearly described and the authors provide sufficient context for the reader to understand their potential uses. The authors also mention previous work based on the showcased data. I highly appreciate that the authors offer their tool freely, and that they provide the necessary information for all the data pre-processing as well as offer the readers example data for testing. For their tool to be widely used and appreciated by the community, I would suggest creating a user friendly interface for their software tool.
The authors have presented appropriate references to the literature, but they have omitted an important publication in the field [https://doi.org/10.1038/s41592-021-01198-0], which uses a similar concept for multimodal registration. The methods section was well rounded, but one missing element was the information, tool, database or library they used for annotating several m/z values: e.g. lines

275-278, and lines 307-309.
The authors have strengthened their tool by using an orthogonal imaging method for reducing instrumental noise or acquisition artifacts, which I found very valuable as many research teams in the field already use staining protocols in their studies. The information obtained from stained tissues (H&E, IHC) is highly valuable for targeted studies, but it is limited for untargeted ones. Have the authors considered testing their tool on vibrational spectroscopy imaging data like FTIR or Raman? In this case the imaging can be done on the same section with increased ease for multimodal registration and minimal deformation between imaging modalities. Also, these techniques are providing additional, and orthogonal, molecular signatures.
The writing is clear, easy to follow, with minimal editing required: e.g. lines 182 "section and SI", and line 354 "more in section": there is a missing pointer to the section in each case.
The data are presented in an appropriate manner, figures are representative of the described results, and almost ready to be published: Figure 2 should be prepared with the same font size as the other figures, and Figure 4 has a closed parenthesis ")" in the bottom right corner which should be removed.
Some considerations:
I am missing "clean" optical or H&E images before multimodal registration (without the applied deformation mesh) to assess the quality of the tissue sections and the multimodal registration result. Figure 5 (b) contains the only representation of the two tissue sections, where the MSI NMF score map seems to showcase a more intact tissue section, compared to the H&E score map, where the holes and tears in the tissue are visible. Could these missing pixels from the MSI data after registration omit some valuable information from the tissue? Figure S4 illustrates that the regions of the holes and tears are present in the MSI imaging data only after multimodal registration. In this context, how do you validate the registration of the two images?
On page 5 lines 312-315, the statement "By comparing the MSI- and histology-segmentation results, we became able to automatically and quantitatively determine to which extent that molecular variation could be explained by underlying histomorphological variation." gives the impression that obtaining the #clusters was also done in a quantitative manner, e.g. by multiple adjacent sections' analysis. Please consider rephrasing.

Methods

Are the methods appropriate to the aims of the study, are they well described, and are necessary controls included? Choose an item.

Conclusions

Are the conclusions adequately supported by the data shown? Choose an item.

Reporting Standards

Does the manuscript adhere to the journal’s guidelines on [minimum standards of reporting?] Choose an item.

Choose an item.

Statistics

Are you able to assess all statistics in the manuscript, including the appropriateness of statistical tests used? Choose an item.

Quality of Written English

Please indicate the quality of language in the manuscript: Choose an item.

Declaration of Competing Interests

Please complete a declaration of competing interests, considering the following questions:

- Have you in the past five years received reimbursements, fees, funding, or salary from an organisation that may in any way gain or lose financially from the publication of this manuscript, either now or in the future?
- Do you hold any stocks or shares in an organisation that may in any way gain or lose financially from the publication of this manuscript, either now or in the future?
- Do you hold or are you currently applying for any patents relating to the content of the manuscript?
- Have you received reimbursements, fees, funding, or salary from an organization that holds or has applied for patents relating to the content of the manuscript?
- Do you have any other financial competing interests?
- Do you have any non-financial competing interests in relation to this paper?

If you can answer no to all of the above, write 'I declare that I have no competing interests' below. If your reply is yes to any, please give details below.

I declare that I have no competing interests

I agree to the open peer review policy of the journal. I understand that my name will be included on my report to the authors and, if the manuscript is accepted for publication, my named report including any attachments I upload will be posted on the website along with the authors' responses. I agree for my report to be made available under an Open Access Creative Commons CC-BY license (http://creativecommons.org/licenses/by/4.0/). I understand that any comments which I do not wish to be included in my named report can be included as confidential comments to the editors, which will not be published.

Choose an item.

To further support our reviewers, we have joined with Publons, where you can gain additional credit to further highlight your hard work (see: https://publons.com/journal/530/gigascience). On publication of this paper, your review will be automatically added to Publons, you can then choose whether or not to claim your Publons credit. I understand this statement.

Yes Choose an item.
